# Benthic megafaunal biodiversity of the Charlie-Gibbs fracture zone: spatial variation, potential drivers, and conservation status

**DOI:** 10.1007/s12526-022-01285-1

**Published:** 2022-09-26

**Authors:** Poppy Keogh, Rylan J. Command, Evan Edinger, Aggeliki Georgiopoulou, Katleen Robert

**Affiliations:** 1 Geography Department, Memorial University of Newfoundland and Labrador, St. John’s, Canada; 2 Fisheries and Marine Institute, Memorial University of Newfoundland and Labrador, St. John’s, Canada; 3 School of Environment and Technology, University of Brighton, Brighton, UK

**Keywords:** Sponges, Corals, Deep sea, GAMs, North Atlantic

## Abstract

The Charlie-Gibbs Fracture Zone (CGFZ) is a prominent geological feature offsetting the Mid-Atlantic Ridge (MAR), consisting of two parallel fractures, creating a highly variable seafloor bathymetry. It has been defined as the most important latitudinal biodiversity transitional zone on the MAR. Despite this recognition, the faunal communities living on the fracture zone have not been extensively described. A remotely operated vehicle (ROV) was utilised during the TOSCA (Tectonic Ocean Spreading at the Charlie-Gibbs Fracture Zone) survey. The survey included five ROV video transects at depths between 560 and 2900 m. The objectives of the study were to use this video footage to quantify benthic megafaunal density and biodiversity patterns on the CGFZ and their environmental drivers. Species accumulation curves and generalised additive modelling show that depth and the presence of hard substrates play an important role in explaining species richness and abundance at the CGFZ. Coral taxa showed highest abundance between depths of 1500 and 2000 m, while sponge taxa were more abundant between 1750 and 2250 m. A dense sponge aggregation was identified on a ridge feature at 2250 m depth. The high biodiversity and presence of dense sponge aggregations and coral gardens found in this study highlight the need for detailed surveys to help support decisions made by governing bodies on the protection status of the CGFZ.

**Supplementary Information:**

The online version contains supplementary material available at 10.1007/s12526-022-01285-1.

## Introduction

There has been a significant increase in deep sea exploration and research from the last two decades to enhance our knowledge of diverse marine ecosystems, such as cold water coral reefs (Buhl-Mortensen et al. 2010; Roberts et al. 2006) coral gardens (Bullimore et al. 2013) and sponge aggregations (Hawkes et al. 2019; Howell et al. 2016). Gathering knowledge on the biological composition and geographical distribution of these ecosystems is the first step towards developing coherent management and protection plans (Ardron and Secretariat 2014). However, areas beyond national jurisdiction (ABNJ) remain relatively understudied (Blasiak and Yagi 2016). These environments are especially difficult to study, due to their remoteness leading to the high cost of data collection (Serrano et al. 2017). Recent studies have looked at potential issues with effectively protecting ABNJ, such as how to determine the criteria for identifying ecologically significant areas and emplacing protection regulations in these remote ocean areas (Long and Chaves 2015; Mossop 2018). Solutions to these issues have begun to be discussed in recent years, for example, the expansion of the European Union’s Marine Strategy Framework Directive to include guidelines on managing biodiversity beyond national jurisdictions (Orejas et al. 2020).

**Fig. 1:**
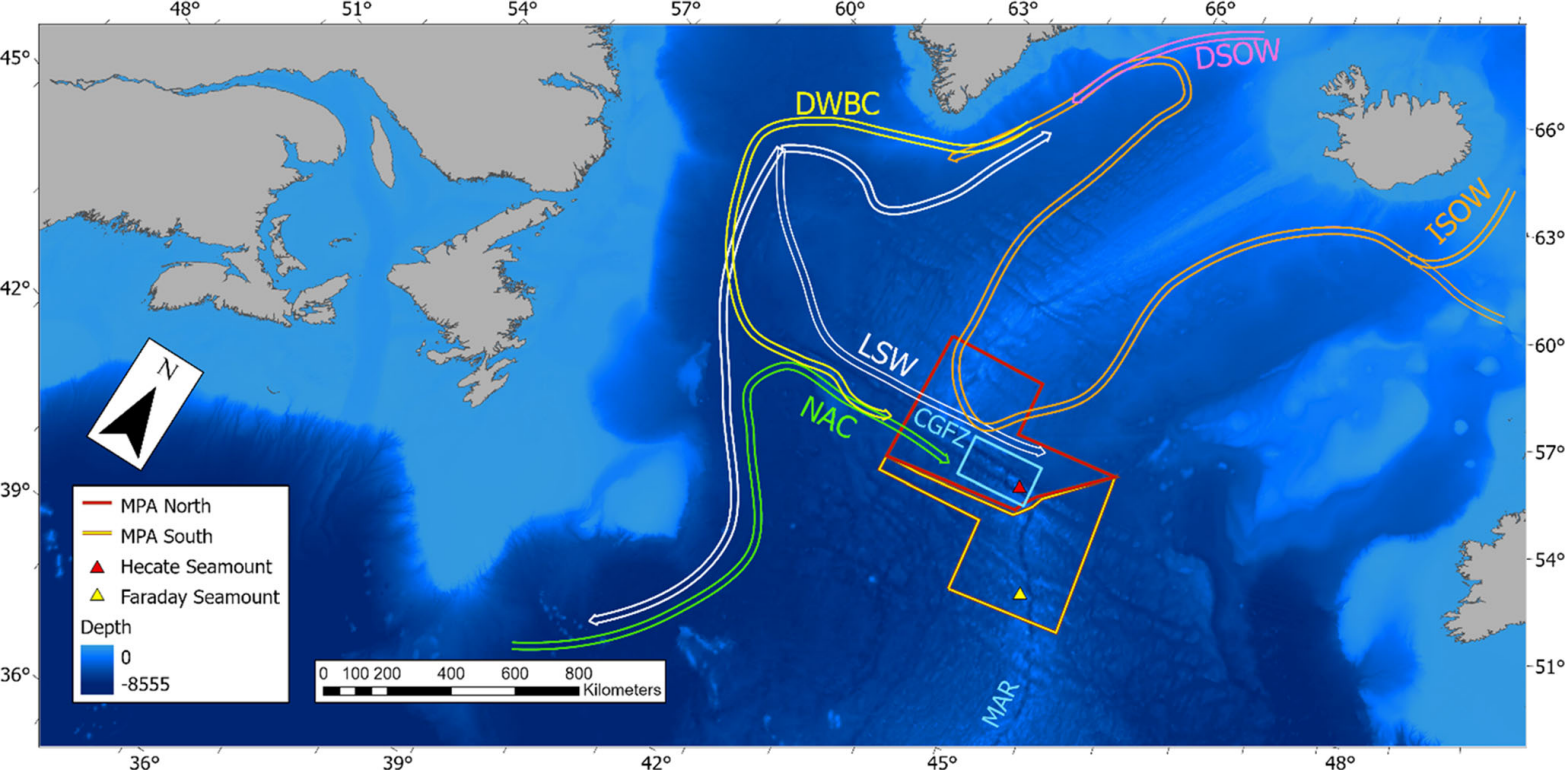
Location of Charlie-Gibbs Fracture Zone (CGFZ), the Hecate and Faraday seamounts and study location for the TOSCA survey (in blue box) in the North Atlantic Ocean, on the Mid-Atlantic Ridge (MAR). The boundaries of the North and South CGFZ marine protected areas (MPA) are shown in red and yellow, respectively. The North Atlantic Current (NAC) is shown in green. The Deep Western Boundary Current (DWBC) is shown in yellow, the Labrador Sea Water (LSW) is in white, the Iceland–Scotland Overflow Water (ISOW) is in orange, and the Denmark Strait Overflow Water (DSOW) in pink (Racapé et al. 2019; Schott et al. 1999). Background bathymetry sourced from the General Bathymetric Chart of the Oceans (GEBCO).

Although slightly better known than other ABNJ (Coro et al. 2016), the North Atlantic still has many knowledge gaps regarding the spatial distribution of deep ecosystems, and lacks uniformity in the collection of deep sea data (Kazanidis et al. 2020). The Mid-Atlantic Ridge (MAR), which divides the North Atlantic into eastern and western ocean basins, creates a biogeographic boundary that has a considerable effect on the biological communities inhabiting this region (Alt et al. 2019; Bell et al. 2016; Gebruk and Krylova 2013; Priede et al. 2013). The Charlie-Gibbs Fracture Zone (CGFZ) is an area of two parallel transform faults (the Charlie and the Gibbs) that offsets the MAR by over 340 km, making these the longest faults in the North Atlantic (Fig. 1), and which has also been recognized as an important biogeographic boundary (Calvert and Whitmarsh 1986; Gebruk et al. 2010). Previous studies have looked at the differences in species composition and abundance on the MAR and found significant differences between the north and south of the CGFZ (Alt et al. 2019; Bell et al. 2016; Gebruk and Krylova 2013). There is still limited information on the biodiversity of the CGFZ region itself as previous studies included little sampling between the two transform faults. The only prior study of the megabenthic species of the CGFZ using video that we are aware of involved the analysis of 13 5-minute transects, collected with a submersible as part of the ‘Census of Marine Life’ project (MAR-ECO) in 2003 (Gebruk and Krylova 2013).

As an oceanic core complex, the CGFZ is characterized by a substantial amount of mid-ocean ridge igneous and metamorphic rocks (Skolotnev et al. 2021), providing the hard substratum crucially needed for attachment by many sessile species, including corals (Baker et al. 2012; Bell et al. 2016; Miles 2018; Mortensen et al. 2008; Robert et al. 2015). This diverse fauna includes reef-forming Scleractinians and Octocorals, as well as Demosponges, Hexactinellids, stalked crinoids and sessile or slow-moving holothurians. Biologically, the CGFZ core complex is especially important in the region of the MAR as it consists of a large bathyal habitat surrounded on either side by abyssal plains, and the presence of hard substratum would be expected to contribute to habitat heterogeneity for the region and likely lead to a heightened biodiversity (Alt et al. 2019; Priede et al. 2013). Previous coral observations recorded on the MAR in the region of the CGFZ described the presence of *Desmophyllum pertusum* (*Lophelia pertusa*), *Madrepora oculata* and *Solenosmilia variabilis*, although not in mounds or reefs (Mortensen et al. 2008). Species of Octocorals and Antipatharians were also recorded but not as coral gardens (Mortensen et al. 2008). One study described the diversity of hexactinellid sponges between depths of 1700 and 2500 m on the northern slope of the fracture zone, but not of a sponge aggregation specifically on the CGFZ (Gebruk and Krylova 2013).

With the potential for highly diverse and dense biological communities on the CGFZ, anthropogenic threats to this region of the North Atlantic need to be carefully addressed to mitigate the risks of long-term damages. One of these risks includes the potential for future mining on the MAR (Cherkashova et al. 2010). In 2015, an area of the MAR was used in a theoretical case study by the International Seabed Authority, to investigate the use of “Areas of Particular Environmental Interest” or APEIs on mid-ocean ridges to mitigate the impacts of mining (Dunn et al. 2018). This same area, described by Dunn et al. (2018), currently has three ongoing seafloor massive sulphides, or polymetallic sulphides exploration contracts (Cherkashova et al. 2010; Murton et al. 2019). In the study looking at the implementation of APEIs on the MAR, it was stated that these should include bathymetric features of ecological importance, one of which being major transform faults that connect the east and west basins of the North Atlantic (Dunn et al. 2018).

One way to protect APEIs would be the implementation and effective regulation of a coherent network of marine protected areas (MPA). As of 2012, the CGFZ was split into two MPAs, the CGFZ South MPA and the CGFZ North MPA (Smith and Jabour 2018). The South MPA is under full protection from anthropogenic activities, including the water column, the seafloor and the subsoil, while the CGFZ North MPA is only partially protected, due to an outstanding submission from Iceland to extend the boundary of their exclusive economic zone (Hübner and von Nordheim 2019; Smith and Jabour 2018). Hence, the seafloor and subsoil, including all benthic communities, of the CGFZ North MPA remain unprotected from anthropogenic activities. In 2022, these activities could include commercial fisheries, as the North East Atlantic Fisheries Organisation (NEAFC) will be reviewing the fisheries closure that has been instated in this region since 2009 (Hübner and von Nordheim 2019). Up until the 1990s, the CGFZ was host to multiple fisheries dominated by a Soviet/Russian fishing effort, targeting populations of demersal deep water fish including the roundnose grenadier, redfish, orange roughy and numerous shark species (WWF 2008). If extended, the closure of this area to bottom trawling would be an integral part of the future preservation of the CGFZ benthic ecosystem.

The objective of this study was to describe megabenthic taxa abundance and diversity of the CGFZ and to determine what environmental factors influenced biodiversity in this area to help inform future sampling efforts and MPA management decision making. These analyses were done using ROV (Remotely Operated Vehicle) video collected aboard the TOSCA (Tectonic Ocean Spreading at the Charlie-Gibbs Fracture Zone) expedition.

## Material and methods

### Study site

The CGFZ is topographically unique as it includes north-south and east-west bathymetric barriers (the fracture zone itself and the MAR axis, respectively) (Gebruk and Krylova 2013). The North Atlantic Current crosses the MAR over the CGFZ at 53°N (Fig. 1), which determines the boundary of the Sub-Polar Front at its northernmost point and creates an oceanographic boundary to the north and south due to differing water masses (Alt et al. 2019; Priede et al. 2013; Read et al. 2010). The Subarctic Intermediate Water makes up the surface layer and is brought in by eastward flow, resulting in the freshest Labrador Sea Water occurring between 1000 and 1500m (Schott et al. 1999; Shor et al. 1980). The deeper water mass is the Iceland–Scotland Overflow Water (Fig. 1), found below 2000m and originating from the Iceland–Scotland Ridge, in the North East Atlantic (Racapé et al. 2019; Schott et al. 1999). The Iceland–Scotland Overflow Water is driven west through the CGFZ by the Deep Western Boundary Current (Racapé et al. 2019; Read et al. 2010; Saunders 1994). This complex oceanography may have positive effects on faunal diversity and distribution, by transporting organic matter to the deeper portions of the fracture zone.

The CGFZ has been examined for its unique geological characteristics, including the two left lateral transform faults which are connected by a 40-km wide gap, also known as the intra-transform spreading centre (Skolotnev et al. 2021). The CGFZ is characterized by multiple large oceanic core complexes, which only form at slow spreading oceanic plate boundaries that have a limited supply of upwelling magma, such as at the MAR (Georgiopoulou et al. 2018; MacLeod et al. 2009; Skolotnev et al. 2021). These geological features create a unique and possibly ecologically important substratum for the sessile benthic megafauna and associated communities of the CGFZ.

### Data collection

**Fig. 2:**
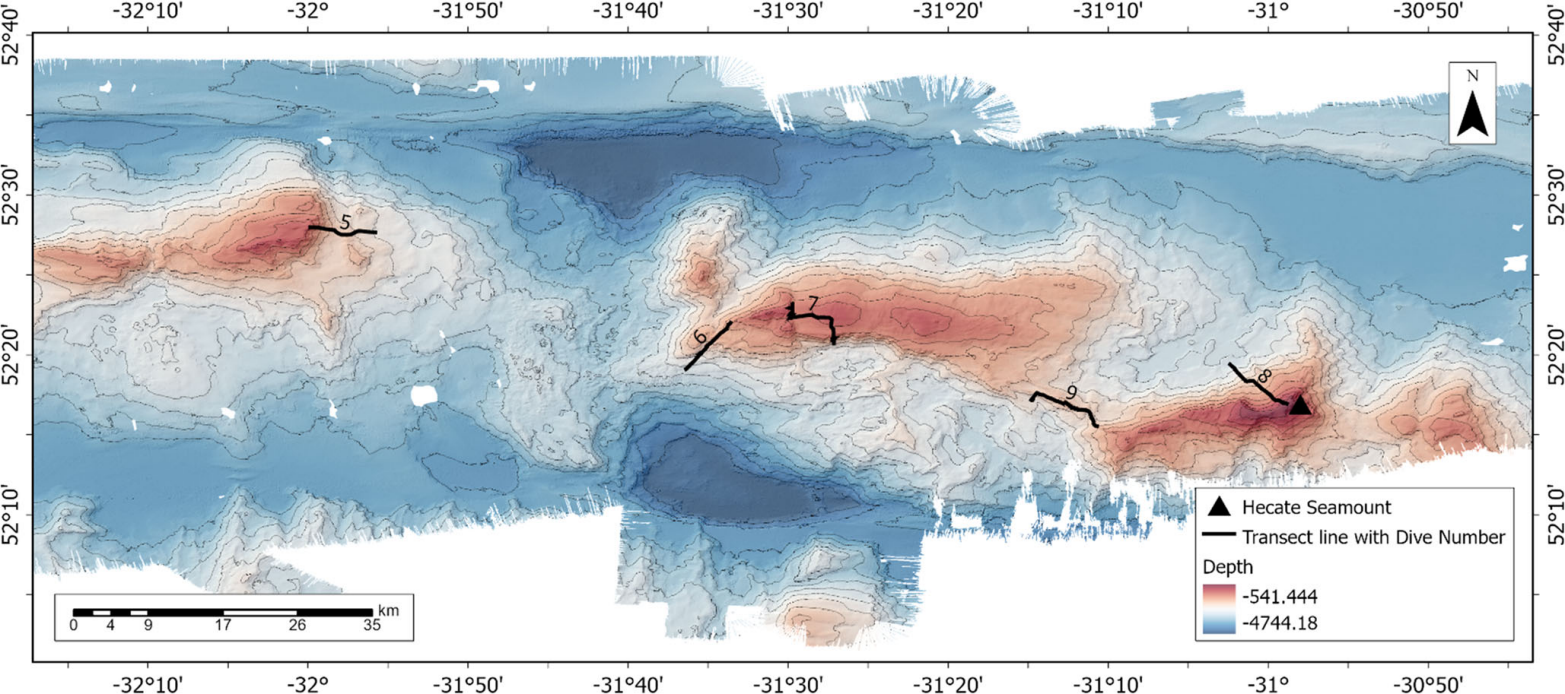
Detailed bathymetry of the Charlie-Gibbs Fracture zone and ROV video transect locations. Map shows TOSCA ROV transect lines as well as the ship-borne bathymetry of the area (30m resolution). Contour lines are at 250-m depth intervals. Location of the Hecate Seamount is represented with a black triangle. For location, see black box in Fig. 1

The ROV *Holland I* was utilised during the TOSCA expedition aboard the *Celtic Explorer* Research Vessel in 2018 (CE18008). The *Holland I* has a maximum depth range of 3000 m. HD videos were recorded along five ROV transects (Table 1 and Fig. 2), with a high-definition oblique-facing camera (Kongsberg Maritime OE14-502a HDTV inspection camera), recording in 1080i resolution, at 25 frames per second with up to 7 phase alternating lines, to Ki-Pro disks in 2-h segments. A 5 mega-pixel, OE 14366 Colour Zoom Camera recorded still images of observed organisms. The position of the ROV was continuously recorded using Ultra Short Baseline (USBL) systems (IXSEA GAPS USBL and Sonardyne Ranger 2 USBL). Shipboard bathymetry data was collected during the survey using the vessel’s Kongsberg EM302 multi-beam echo-sounder (MBES), processed in Caris HIPS & SIPS, and exported to raster (projected as UTM Zone 25, 30 m resolution). A Seabird ROV-mounted CTD (conductivity, temperature, depth) acquired data throughout all dives, logged using SeaSave 7 and converted to ASCII (American Standard Code for Information Interchange) using SBEDataProc. During the last 1150 km of dive 9, the CTD malfunctioned, and subsequently no CTD data was available for this portion. The ROV aimed to fly at 0.5 knots at an altitude of 1.5 m above the seabed, throughout the five dives (Table 1). A total of 67.5 hours of seabed video over 34 km, equaling 3.32 TB of HD video were collected.

**Table 1:** Remotely Operated Vehicle dive information

	ROV05	ROV06	ROV07	ROV08	ROV09
Start date	25/05/2018	30/05/2018	31/05/2018	01/06/2018	02/06/2018
End date	30/05/2018	30/05/2018	31/05/2018	01/06/2018	03/06/2018
Start time (UTC)	14:01:53	6:52:51	0:59:56	0:28:37	11:53:57
End time (UTC)	1:06:06	19:39:27	19:02:13	12:48:18	1:54:31
Start latitude (at the bottom)	52.46132029	52.3178975	52.3449766	52.3236988	52.2761865
Start longitude	−31.92992534	−31.606659	−31.4523683	−31.0403533	−31.1969
End latitude (off the bottom)	52.46610562	52.3668641	52.38680367	52.28240853	52.2586104
End longitude	−31.99858147	−31.5599112	−31.49370983	−30.98067911	−31.1784563
Total length of each transect (m)	~8600	~8100	~8350	~6550	~6200
Total no. of 50-m sections	172	162	167	131	124
Start depth (m)	2533	2870	2199	2965	2412
End depth (m)	2474	1597	1420	561	1908
Average temperature (°C)	3.282	3.216	3.569	3.401	3.311
Average salinity (PSU)	34.932	34.937	34.899	34.861	34.926
Total no. of organisms	12,234	21,207	45,344	24,629	51,096
Total no. of morphospecies	197	185	189	199	210

### Video analysis

The VARS (Video Annotation and Reference System) software developed by the Monterey Bay Aquarium Research Institute was utilised to annotate the ROV video transects (Schlining and Stout 2006). Lasers beams with a 100-mm spacing were present in all videos for scaling purposes. Organisms larger than 20 mm were identified and assigned to a morphospecies when species level identification could not be achieved due to the limitations of relying on video or still imagery alone (Howell et al. 2019). A species catalogue was created from still images collected during the ROV dives and was used as a reference for morphospecies occurrence throughout video analysis (see supplementary material). Resources used for the catalogue included the Catalogue of Atlantic Deep Sea Fauna (Howell et al. 2017), the Benthic Deepwater Animal Identification Guide V3 (NOAA 2015) and species catalogue within PhD thesis (Alt 2012), while the nomenclature employed was based on WoRMS (World Register of Marine Species). Taxonomic experts (see acknowledgements) were contacted for the identification of the sponges and cnidarians, as well as special groups such as Pennatulacea and Pycnogonida. Substrate types were also recorded according to the EUNIS (European Nature Information System) classification system for deep sea seabed categories, which includes bedrock, boulders, mixed substrate (gravels), biogenic gravels and sand (Moss 2008).

Frames were extracted from the ROV videos at a rate of 1 every 5 seconds using Blender, and the two laser points were used to estimate frame width to convert observed abundances into densities.

### Statistical analysis

Species observations, substrate types, CTD data (logging information on temperature and salinity) and laser measurements were georeferenced using the USBL data from the ROV. This was done in the R program (version 3.6.2), using the ‘*eXtensible*’ Time Series package. Based on the depth obtained from the shipboard multi-beam data, slope was derived, at 30 m resolution, using the Benthic Terrain Modeller toolbox in ArcGIS Pro. ROV transects were subdivided into 50-m sections (*n*=756) for the statistical analysis, and the midpoint coordinate of each section was used to extract slope and depth values. All organisms present within a 50-m section were summed while the CTD data (temperature and salinity) and image width derived from the lasers were averaged for each section. Image width was combined with segment length (50 m) to derive areas for each section. The estimated area, based on image width and segment length, was used to convert to the morphospecies abundance to densities. Each 50 m section was allocated a dominant substrate type by determining the lengths of coverage for each substrate within a section.

The 50 m sections were used to derive species accumulation curves using the *specaccum* function from the ‘*vegan’* package in the R program (Oksanen et al. 2020). Species accumulation curves were created for each dive, 250 m depth bands (decided based on the depth range (500 to 3000 m) and to allow enough samples to be included within each band while still retaining enough bands) and each substratum class that was observed. Species accumulation curves allow the examination of the expected number of observed species as a function of sampling effort (Gotelli and Colwell 2001). Diversity indices for each 50 m section (Shannon-Wiener H-index, Species Richness and Pielou’s Evenness) were calculated using the ‘vegan’ package. This was done for all observed morphospecies together as well as cnidarians and sponges separately because of their presumed ecological significance.

To examine further what environmental factors might be influencing biodiversity, generalised additive models (GAM) were employed. GAMs allow for modelling non-linear trends using smooth functions of covariates (Wood 2011). Recent developments allow for the modelling of nested data, as well as spatial and temporal autocorrelation, using random effect smooths. Factors modelled as random effects are assumed to be a random sample of factor levels from a population of possible levels, and the intercept or shape of the modelled relationship is allowed to vary by factor level (Wood et al. 2016). Collectively, GAMs with nested structure are known as hierarchical generalised additive models, or HGAMs (Pedersen et al. 2019).

Here, Phyla abundance, species richness, Shannon-Wiener H-index and Pielou’s evenness were modelled using HGAMs. For abundance and species richness, models were fit using a negative binomial distribution to account for overdispersion, which is common in ecological data (Barry and Welsh 2002), and to preserve the inherent count distribution in these variables. The total area covered by the camera field of view was included as an offset term in the richness and abundance models to preserve the count distribution of the response variables. For Shannon-Wiener H-index and Pielou’s evenness, HGAMs were fit using only the non-zero observations and using the Gaussian distribution for the Shannon-Wiener H-index and the scaled-*t* distribution for Pielou’s evenness. Each response was modelled as smooth functions of depth and slope. A tensor product smooth of latitude and longitude was used, with dive as a random effect, to account for spatial autocorrelation that may be present in the data, and to allow for correlation of observations within each dive. Models were fit using Restricted Maximum Likelihood Estimation to estimate smoothing parameters, since it is generally considered to be the most numerically stable (Wood 2011; Wood et al. 2016).

After fitting HGAMs, model fit was assessed by examining residual plots and checking for concurvity. Concurvity can be thought of as a non-linear extension of multicolinearity (Connolly et al. 2013; Figueiras et al. 2005). Two variables are said to be concurve when a smooth function of one can be reconstructed using a smooth function of another variable. This influences parameter and standard error estimates analogous to the multicollinearity problem.

## Results

### Composition of all taxa

**Fig. 3:**
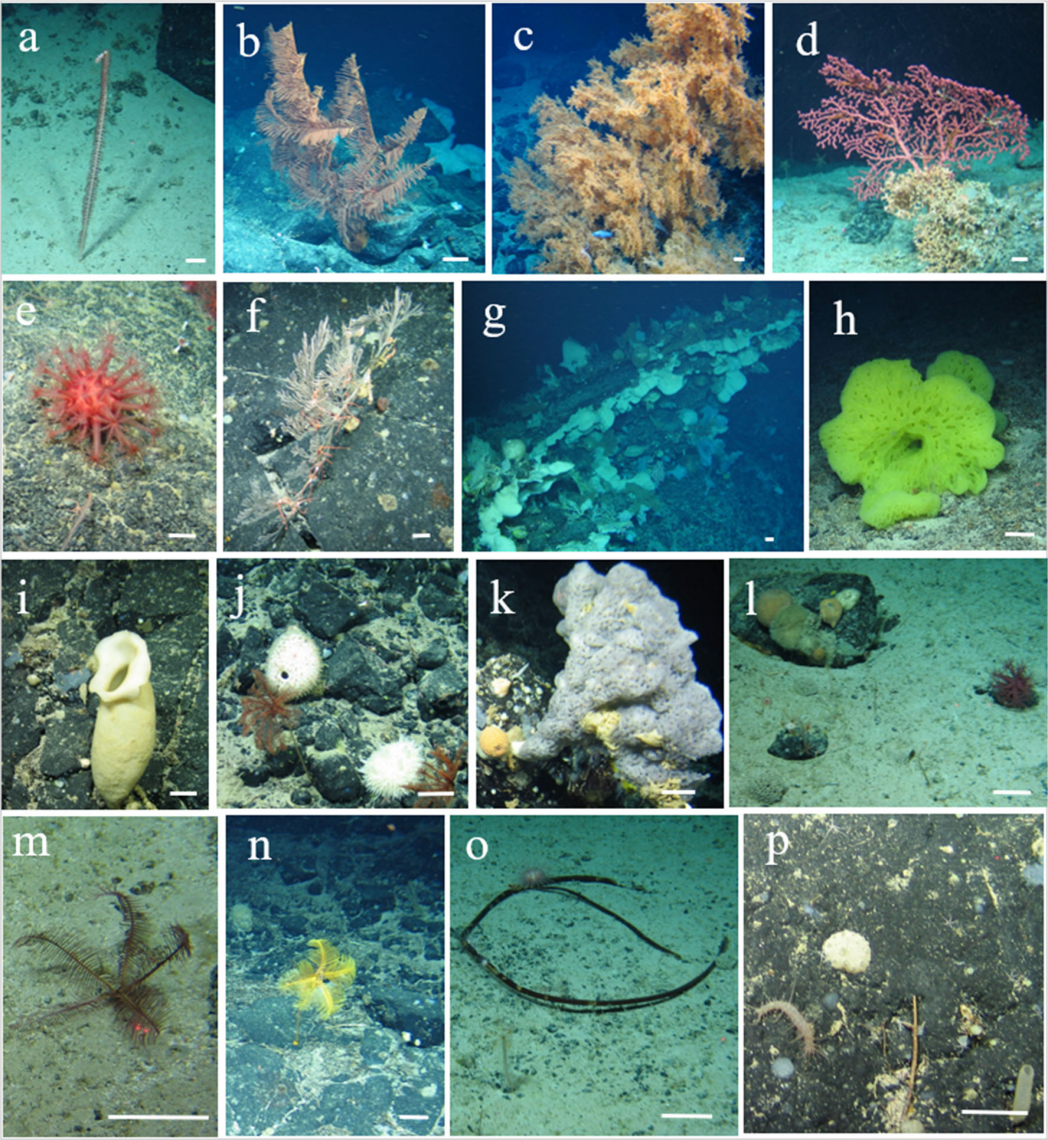
Megabenthic fauna of the Charlie-Gibbs Fracture Zone. White bar for scale is 100 mm. **a** *Balticina* (*Halipteris*) cf. *finmarchica*; **b** Antipatharia sp.; **c** *Leiopathes* sp. with many fish and crustaceans taking refuge; **d** *Paragorgia* sp. appears to be attached to *Solenosmilia variabilis*.; **e** *Anthomastus* sp.; **f** *Calyptrophora* sp. with multiple Ophiuroids attached; **g** dense sponge aggregation observed on Dive 9; **h** *Hertwigia falcifera,* (yellow colour morph); **i** Hexactinellida sp.; **j** *Geodia* sp. (top left, white) and cf. *Polymastia corticata* (bottom right, white); **k** Hexactinellida sp.; **l** three Xenophyophoroidea can be seen in the bottom left with multiple different Demospongiae morphospecies on the boulder; **m** Crinoidea, *Pentametrocrinus atlanticus*; **n** *Anachalypsicrinus nefertini* on bedrock; **o** *Echinus* sp. observed in top right, alongside other Echinoidea morphospecies feeding on what appears to be kelp, Bathycrinidae (Family) sp. in bottom left; **p** Holothurian, cf. *Synallactes* sp. (bottom left) and Euplectella sp. (bottom right)

A total of 154,509 individual organisms belonging to 309 megafaunal morphospecies (metazoan and protistan) were identified from ROV video collected aboard the TOSCA expedition (example morphospecies shown in Fig. 3 and full species catalogue can be found in the supplementary material), throughout five ROV dives (see Table 1). This is likely an underestimation due to the difficulty associated with identifying megafauna from video, and the potential presence of cryptic species. The five most abundant morphospecies (number of individual organisms observed for each morphospecies (*n*)) across all five ROV dives were Xenophyophore spp (*n*= 23,616), a stalked Crinoid, possibly Bathycrinidae (Family) sp. (*n*= 15,952), the Bryozoan *Canda* sp. (*n*= 13,261), an encrusting Demosponge morphospecies (*n*= 11,395) and a Holothurian, *Psolus* sp. (*n*= 11,133). Cnidarians contained the largest number of morphospecies, but they did not represent that many individuals overall, indicating the presence of many rare species. Sponges were the most abundant phylum in terms of number of individuals, with 39% of the individual organisms observed being part of the phylum Porifera, indicating their importance at the CGFZ. Xenophyophores were frequently observed but could only be assigned to a single morphospecies. The Echinoderms were an important group at the CGFZ due to their high abundance (*n*= 40,077).

Almost one-third of all morphospecies observed in this analysis could be considered rare, with 28% of morphospecies (86 of 309) observed fewer than ten times, and 6% (19 morphospecies) seen only once in all five dives. The Phyla that were the most taxonomically rich (number of morphospecies in each group (*z*)) included cnidarians (*z*= 116), sponges (*z*= 77) and Echinodermata (*z*= 65). Echinodermata consisted of 23 Asteroidea morphospecies, 14 Crinoidea morphospecies, 7 Echinoidea morphospecies, 8 Ophiuroidea and 13 Holothuridea. Chordata had a total of 31 morphospecies, which included only one morphospecies of Tunicata and 30 belonging to Gnathostomata, including morphospecies from Actinopterygii (*z*= 25), Elasmobranchii (*z*= 3) and Holocephali (*z*= 2). The Phyla with lower morphospecies richness included Arthropoda (*z*= 10), Bryozoa (*z*= 3), Foraminifera (xenophyophores) (*z*=1) and Other (z=6). “Other” includes Mollusca (Decapodiformes and Bivalvia) and Annelida (*Bonellia* sp., Sabellidae), and one organism that could not be identified to Phylum level.

### Biodiversity and spatial patterns

**Fig. 4:**
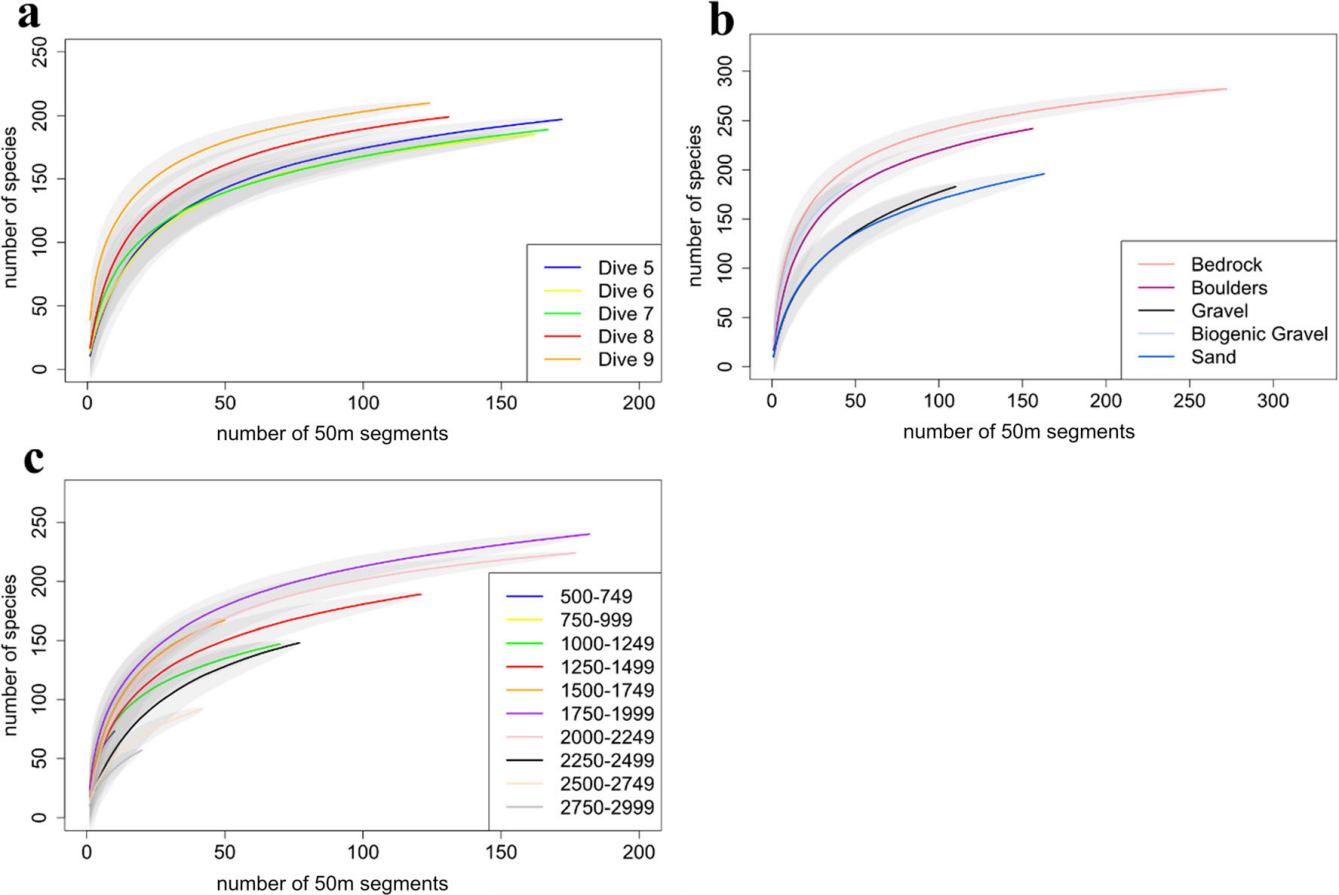
**a** Species accumulation curve for all ROV dives, **b** for each substrate type (biogenic gravel includes coral rubble) and **c** for 250 m depth bands ranging from 500 to 2999 m. *Y*-axes are number of morphospecies observed; *X*-axes are the number of 50 m segments sampled. Shaded polygons represent 95% confidence intervals

**Fig. 5:**
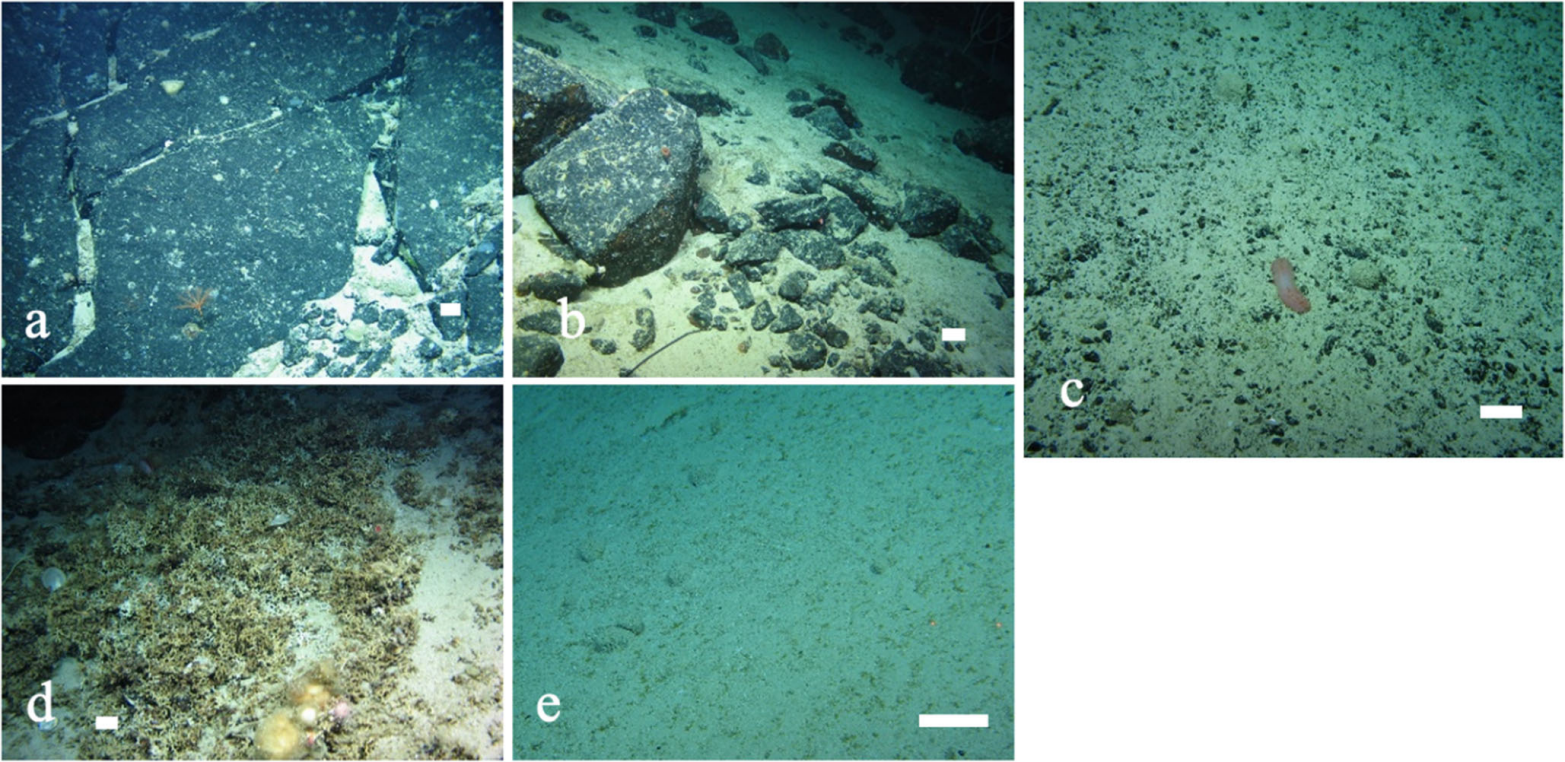
Representative images of substrate types at the Charlie-Gibbs Fracture Zone: **a** Bedrock; Dive 9 at approx. 2200 m; **b** boulders, Dive 8 at approx. 2500 m; **c** gravel, Dive 6 at approx. 2000 m; **d** biogenic gravel (includes coral rubble), Dive 5 at approx. 2500 m; and **e** sand, Dive 6 at approx. 2000 m. White bars for scale are 100 mm

**Fig. 6:**
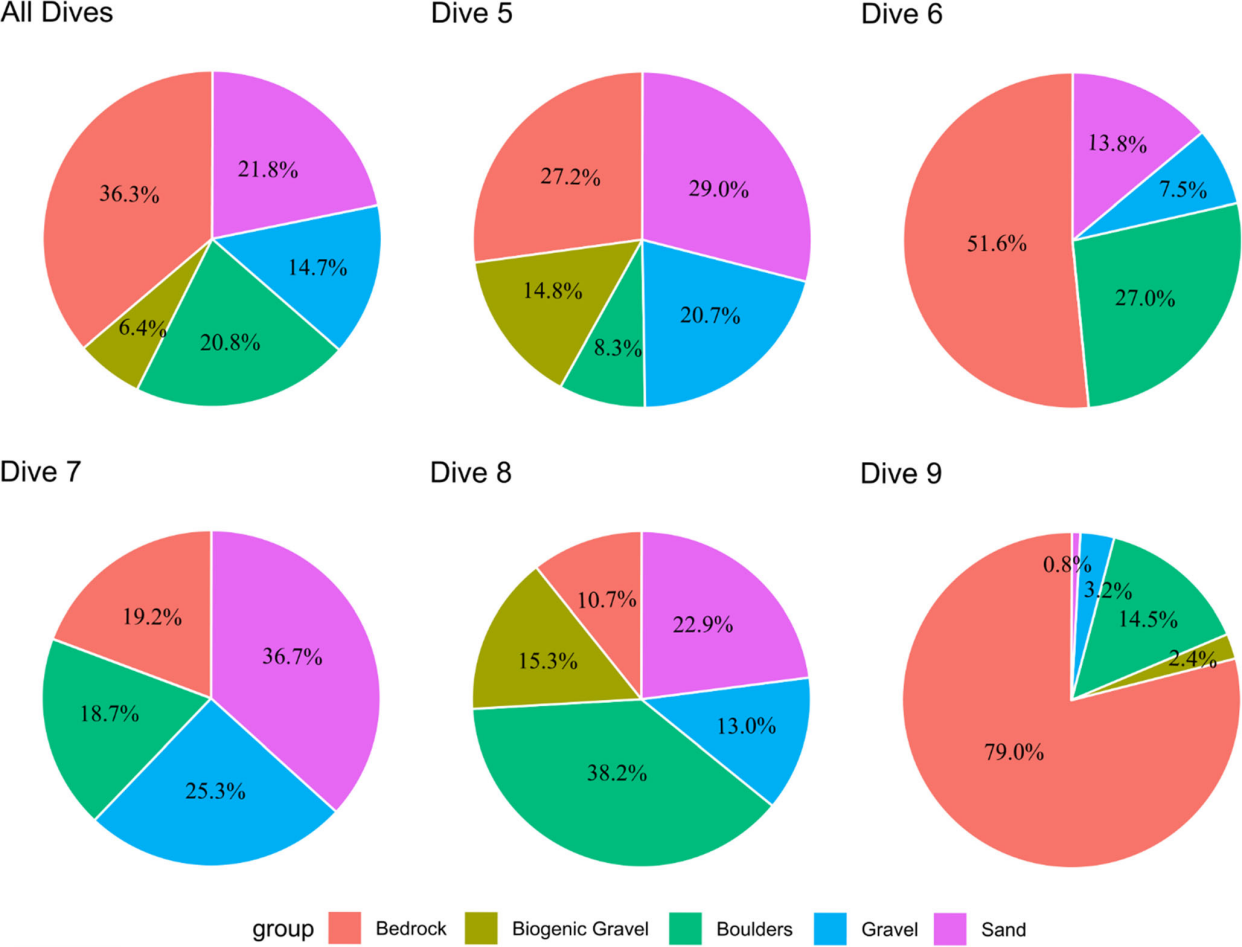
Pie charts showing the proportion of substrate types observed for each dive. Biogenic gravel includes coral rubble

Dive 9 was found to have a slightly higher morphospecies richness, followed by Dive 5 and Dive 8 (Fig. 4a).  A variety of substrate types were observed within the CGFZ (Fig. 5), and the species accumulation curves associated with each of these showed a higher number of morphospecies on bedrock (Fig. 4b). Dive 9, which followed a ridge feature and remained at a relatively constant depth as a result, was found to have a considerably higher ratio of bedrock cover (79%) compared to the other ROV dives (Dive 5 had 27.2%, Dive 6 had 51.6%, Dive 7 had 19.2%, and Dive 8 had 10.7% bedrock) (Fig. 6). Boulders and biogenic gravel had the next highest level of species occurrence after bedrock, although biogenic gravel was not sufficiently sampled, as it only covered approximately 6% of the seafloor throughout all five dives. The species accumulation curve for all morphospecies for each 250 m depth band (Fig. 4c) showed the greatest number of morphospecies were found in three depth bands (1500–1749 m, 1750–1999 m and 2000–2249 m).

### Composition and distribution of cnidarian (corals, sea anemones, cerianthids and hydroids) morphospecies

**Fig. 7:**
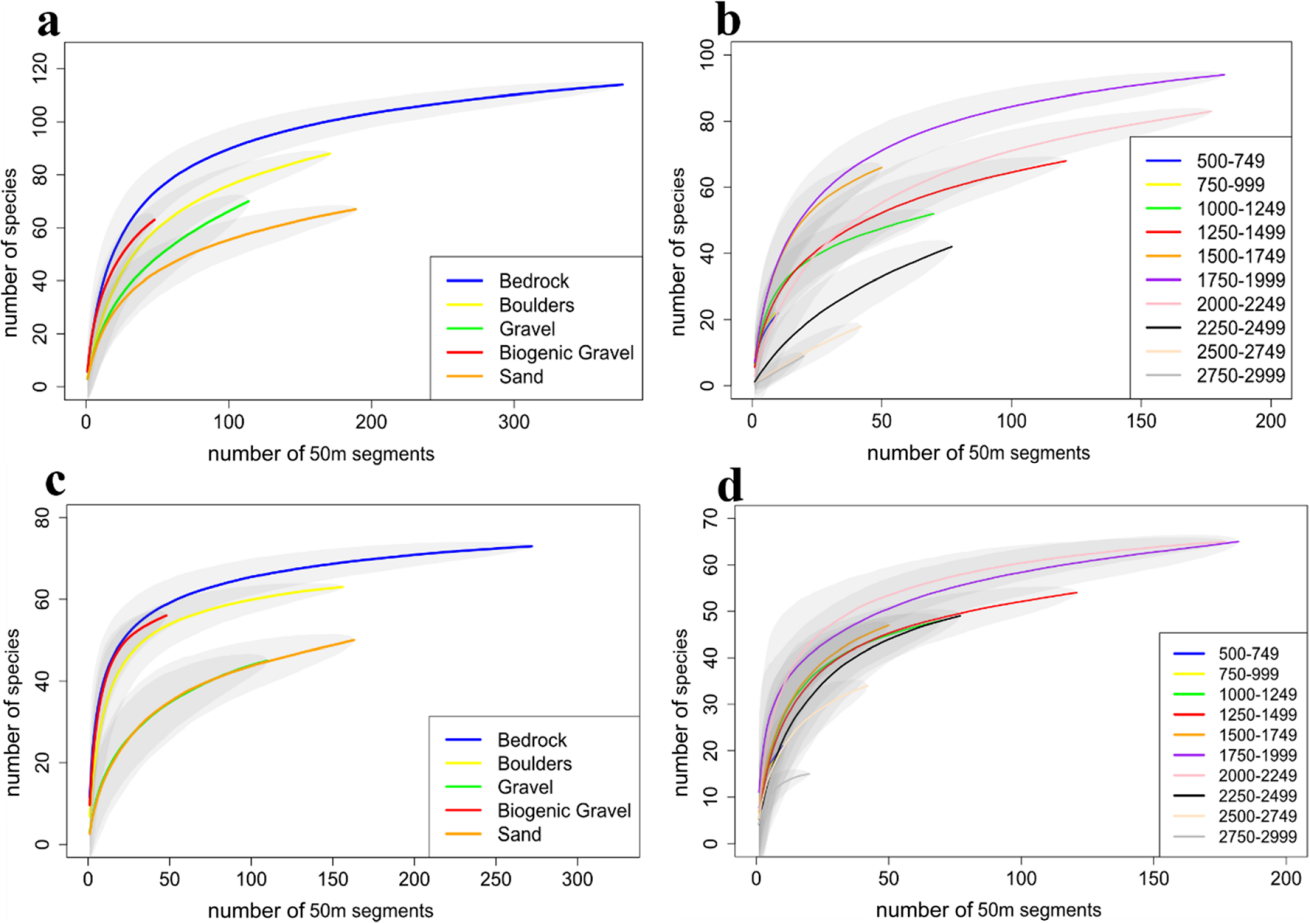
Species accumulation curve for **a** cnidarians (corals, sea anemones, cerianthids and hydroids) by substrate type; **b** 250 m depth band ranging from 500 to 2999 m; **c** sponge morphospecies by each substrate type; and **d** 250 m depth bands ranging from 500 to 2999m. *Y*-axes are the number of species; *X*-axes are the number of 50 m segments. Biogenic gravel includes coral rubble. Shaded polygons represent 95% confidence intervals

**Fig. 8:**
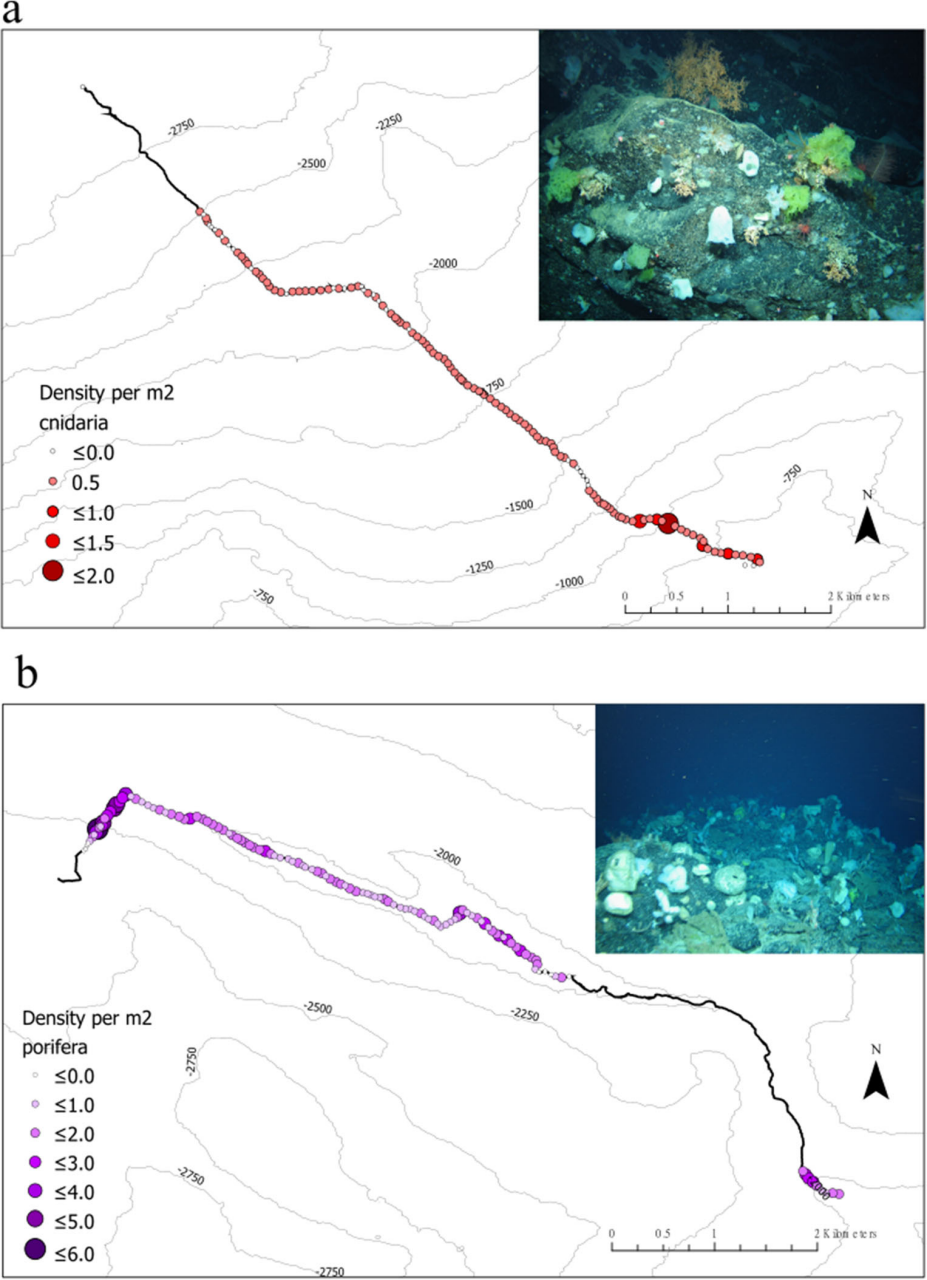
**a** Bubble transect plot showing cnidarian (corals, sea anemones, cerianthids and hydroids) densities on Dive 8 (hecate seamount) and **b** sponge densities on Dive 9 (ridge feature). The image inserts show the areas on each transect with high densities of cnidarians and sponges. Each circle represents cnidarian/sponge observations for a 50 m section of the transect. White circles represent a 50 m section with no observations. No circles present represent the sections of the transect where visibility was too poor to annotate. These sections were removed from the analysis. Density values refer to number of cnidaria/sponges per m^2^

In total, 14,631 individual cnidarian organisms were recorded, belonging to 116 morphospecies from the five ROV dives at depths between 564 and 2884 m (Fig. 3a–f). Octocorallia (Alcyonacea and Pennatulacea) (*z*= 50) were almost equally as rich taxonomically as Hexacorallia (Antipatharia, Actiniaria and Scleractinia) (*z*= 54). Recorded morphospecies of Octocorallia included 16 morphospecies of order Pennatulacea and 31 of order Alcyonacea. Hexacorallia morphospecies were composed of orders Antipatharia with 24 morphospecies, Actiniaria with 21 recorded morphospecies, and Scleractinia with 9 morphospecies, including the reef-building *Solenosmilia variabilis* (Fig. 3d), observed only below 1100 m. In addition to these taxonomically rich groups, one Ceriantharia morphospecies and two Hydrozoa morphospecies were recorded. There were likely multiple cryptic species in these groups, but image quality did not allow for further differentiation. A total of 9 morphospecies were not identified past phylum level. Species accumulation curves for coral morphospecies for each depth band (Fig. 7b) suggested that the greatest number of morphospecies were found at the same depth bands (1500–1749 m, 1750–1999 m, and 2000–2249 m) as those hosting the highest richness for all species combined (Fig. 4). However, the 1500–1749 m band would have benefitted from additional sampling, which can be seen in Fig. 7b and d where the species accumulation curve has not yet reached a plateau. Species accumulation curves for the number of cnidarian morphospecies present per substrate type (Fig. 7a) showed a higher number of morphospecies on bedrock. Dive 8 exhibited a dense cluster of cnidarians, including Scleractinian corals (presumably *Solenosmilia variabilis*), Antipatharians (*Leiopathes* sp., *Bathypathes* sp., and *Stichopathes* sp.) and numerous soft coral morphospecies, near the peak of the seamount, above 1250 m, as can be seen in the species density map (Fig. 8a). The terrain was noticeably steeper here, with some vertical walls present.

### Composition and distribution of sponges

A total of 60,280 individual sponges were recorded. Of the 77 morphospecies, 35 belonged to the Demosponges and 27 morphospecies were within the Hexactinellids (Fig. 3g–k). The remaining Sponges morphospecies could only be identified to phylum level. Sponges made up a total of 39% of the individual organisms observed from this study. Species accumulation curves for sponge morphospecies showed that the greatest number of morphospecies were observed on bedrock, closely followed with biogenic gravels and boulders (Fig. 7c) and higher number of morphospecies were found in two depth bands (1750–1999 m and 2000–2249 m) (Fig. 7d). As could be expected for this taxa, sand and gravel had considerably lower numbers of morphospecies.

A dense sponge aggregation was observed spanning most of Dive 9 (Fig. 8b), between 2400 and 1820 m depth, and supporting many other benthic invertebrates, such as Ophiuroids, Crinoids, Bryozoans and Arthropods. Here, sponge densities were more than 3 sponges per m^2^ for almost 250 m of ROV transect (Fig. 8b).

### Generalised additive model

**Fig. 9:**
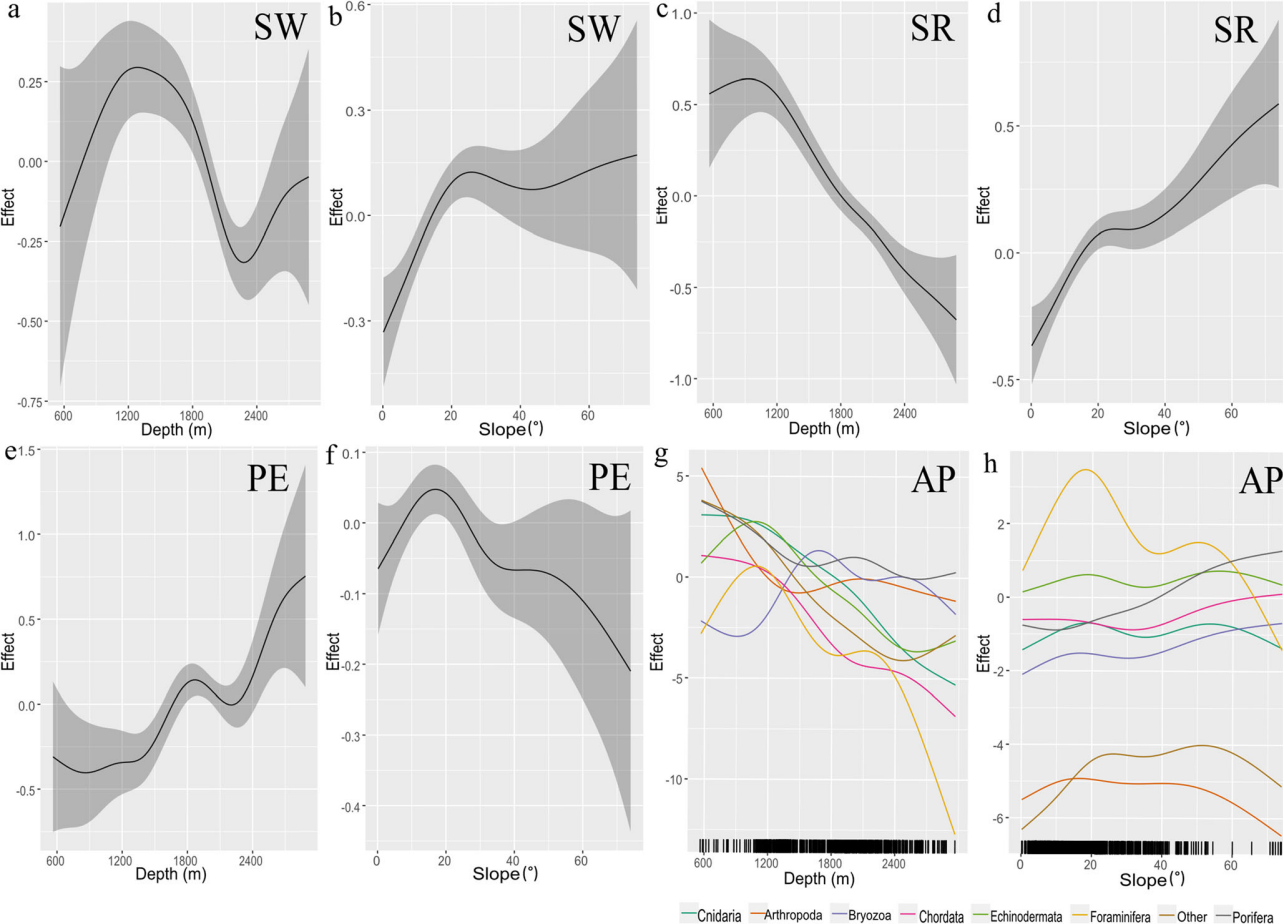
Smooth effects plots from each model. Depth and slope (on *X*-axes) smooth for Shannon-Wiener H-index (SW) (**a**, **b**); species richness (SR) (**c**, **d**); Pielou’s evenness (PE) (**e**, **f**); and abundance by phyla (AP) (**g**, **h**). Hashing on the *x*-axis of panels **g** and **h** refers to number of sample points

Depth and slope had statistically significant effects on the Shannon-Wiener H-index (*p*-value of <0.001). Shannon-Wiener H-index increased with depth down to a maximum at around 1200 m, followed by a decline until 2200 m (Fig. 9a). The Shannon-Wiener H-index increased steadily with increasing slope and then levelled off at a slope value of about 30° (Fig. 9b). The *p*-values for the bedrock, gravel and sand smoothed terms for the Shannon-Wiener H-index were significant (<0.001, Table 2). Depth and slope additionally had significant effects on the species richness (*p*-value of <0.001). Species richness exhibited a continuous decline with depth, below approximately 1100 m, but showed a steady increase as slope values increased (Fig. 9c, d). *P*-values for the bedrock, gravel and sand smoothed terms for species richness were significant, all were <0.001.

**Table 2:** Model summary table for parametric and smooth terms of final models. Intercept term corresponds to the “Bedrock” substrate level

**Shannon-Wiener H-index**				
**Term**	**Estimate**	**Std. error**	***Z*** **value**	***P*-value**
(Intercept) Biogenic gravels Boulders Gravel Sand	1.924 0.02 −0.095 −0.669 −0.598	0.273 0.1 0.061 0.073 0.066	7.055 0.201 −1.553 −9.185 −9.027	<0.001 0.841 0.121 <0.001 <0.001
**Term**	**EDF**	**Ref DF**	**Chi.sq**	***P*-value**
s(depth) s(slope) t2(coords.x1, cords.x2, dive)	5.428 3.564 6.711	6.607 4.414 46	8.185 7.547 6.603	<0.001 <0.001 <0.001
**Species richness**				
**Term**	**Estimate**	**Std. error**	***Z*** **value**	***P*-value**
(Intercept) Biogenic gravels Boulders Gravel Sand	3.105 −0.044 −0.084 −0.413 −0.432	0.266 0.088 0.055 0.069 0.062	11.68 −0.503 −1.542 −6.02 −6.962	<0.001 0.615 0.123 <0.001 <0.001
**Term**	**EDF**	**Ref DF**	**Chi.sq**	***P*-value**
s(depth) s(slope) t2(coords.x1, cords.x2, dive)	4.278 3.884 6.938	5.339 4.809 46	73.28 60.094 399.198	<0.001 <0.001 <0.001
**Taxon abundance**				
**Term**	**Estimate**	**Std. error**	***Z*** **value**	***P*-value**
(Intercept) Biogenic gravels Boulders Gravel Sand	3.986 0.027 -0.353 -0.424 -0.341	3.647 0.133 0.078 0.096 0.087	1.093 0.203 -4.536 -4.418 -3.916	0.274 0.839 <0.001 <0.001 <0.001
**Term**	**EDF**	**Ref DF**	**Chi.sq**	**P-Value**
s(depth) s(slope) t2(coords.x1, cords.x2, dive)	39.502 31.916 6.938	47 47 46	1520.515 292.103 536.547	<0.001 <0.001 <0.001
**Pielou’s evenness**				
**Term**	**Estimate**	**Std. error**	***Z*** **value**	***P*-value**
(Intercept) Biogenic gravels Boulders Gravel Sand	−0.64 −0.018 −0.031 −0.085 −0.157	2.491 0.056 0.034 0.041 0.037	−0.257 −0.318 −0.932 −2.078 −4.26	0.797 0.75 0.351 0.038 <0.001
**Term**	**EDF**	**Ref DF**	**Chi.sq**	***P*-value**
s(depth) s(slope) t2(coords.x1, cords.x2, dive)	7.232 3.951 15.62	8.233 4.882 64	69.513 14.851 383.583	<0.001 0.013 <0.001

Depth and slope significantly affected Pielou’s evenness with a *p*-value of <0.001 and *p*-value of 0.013, respectively (Table 2); Pielou’s evenness started to increase slightly at about 1400 m, then dropped and started to climb again at 2250 m depth (Fig. 9e, f). Only sand was significant for Pielou’s evenness with a *p*-value of <0.0001. When examining the effect of depth on abundance by phyla, all phyla shared a relatively similar trend, where they only slightly decreased as depth increased, except for Foraminifera (xenophyophores) which exhibited a sharp decline at about 2250 m. Bryozoa exhibited a slight increase as depth decreased from about 1000 m (Fig. 9g). Foraminifera exhibited distinct preferences for depth and slope in comparison to the remaining phyla as they decreased more rapidly at depths below 2250 m and beyond slopes of 20 degrees. Boulders, gravel and sand were significant for taxon abundance, with a *p*-value of <0.0001.

## Discussion

Based on ROV video analysis of five transects at the CGFZ, higher levels of morphospecies biodiversity tended to occur at depths between 1500 and 2249 m and in areas characterised by bedrock and steeper slopes. Cnidarians showed the highest richness while sponges showed the highest number of individuals. Three important ecosystem types were encountered, including coral and sponge gardens, and xenophyophore fields. A particularly dense sponge aggregation was observed throughout the ridge feature on Dive 9.

### Biodiversity patterns

Environmental factors, such as water mass transitions and currents present at around 2000 m on the CGFZ may contribute to the increased biodiversity observed at 1500–2249 m. The CGFZ acts as a channel for the transport and western movement of deep water from the eastern North Atlantic (Racapé et al. 2019; Schott et al. 1999; Shor et al. 1980), including the Iceland–Scotland Overflow Water (ISOW) driven west through the CGFZ by the Deep Northern Boundary Current (Read et al. 2010; Saunders 1994). Recent studies have examined the impacts that currents have on the biodiversity of the deep sea benthic ecosystems of the North Atlantic (Johnson et al. 2013; Mohn et al. 2014). These have found that currents play an important role in the lateral transport of food particles at depths below 200 m, especially in areas where primary production may be lacking (Puerta et al. 2020). This has been suggested as leading to the increased occurrence of deep sea filter feeders, such as corals and sponges, in highly hydrodynamic areas (Johnson et al. 2013; Mohn et al. 2014; Puerta et al. 2020). Boundaries between water masses have also been described as important regions for the redistribution of food particles to the deeper layers of the water column when internal waves at the interface of the two water masses mix and move particles down below the boundary layer (Puerta et al. 2020; White et al. 2005), or become trapped at the pycnocline and get moved around by internal waves (Dullo et al. 2008). The location of nepheloid layers, defined as elevated concentrations of suspended particulate matter in the water column, can also be affected by local hydrodynamics and the dominant current systems (Wilson et al. 2015). Past studies show that below the 2000 m mark, the CGFZ is filled mainly with ISOW which carries a substantial load of suspended sediment and is said to create a mid-water nepheloid layer at about 2200 m depth (Schott et al. 1999; Shor et al. 1980). Water mass properties are important factors when considering the occurrence of coral gardens and sponge aggregations and have been suggested as a likely environmental driver in many recent studies (Amaro et al. 2016; Howell et al. 2016; Lacharité and Metaxas 2018; Mohn et al. 2014; Puerta et al. 2020).

There were higher levels of species diversity found on areas of hard substrates (bedrock and boulders) and steep slopes, which is a well documented pattern in the deep sea (Bell et al. 2016; Edinger et al. 2011; Orejas et al. 2009; Robert et al. 2014; Ross and Quattrini 2007). Hard substrates are favourable for sessile suspension and filter feeders such as sponges, corals and stalked crinoids, as it provides a stable attachment surface for optimal food capture (Bell et al. 2016; Mortensen et al. 2008). A high slope value, or areas with steeper slopes, have been found to positively affect the abundance and richness of benthic species due to its association with increased speeds of local currents (Jones et al. 2013). Enhanced local currents increase food particle density and can have positive effects on recruitment (Jones et al. 2013; Palardy and Witman 2011). Dive 9, which exhibited the highest species richness, followed a ridge feature with steep slopes and so the depth remained relatively constant, and was dominated by bedrock. The expanse of hard substratum combined with the likely local-scale topographic interaction with bottom currents (Grigg 1997; Mortensen et al. 2008) appeared to create an ideal environment for an extensive sponge aggregation (Fig. 8b) and coral gardens (gorgonian and black corals, see supplementary material). Comparable levels of biodiversity were also observed in areas of biogenic gravels. Many studies have previously highlighted the association of naturally occurring coral rubble with high levels of biodiversity (Appah et al. 2020; Henry and Roberts 2007; Jonsson et al. 2004). A recent study found the percentage cover of colonial benthic megafauna in the Porcupine Bank Canyon to be four times higher for coral reef and rubble compared to non-reef habitat (Appah et al. 2020). Accumulations of scleractinian coral rubble were observed at several locations on the CGFZ. Judging from the depths at which they were found (Dive 7 shallowest point was 1420 m), it could be *Solenosmilia variabilis* rubble, possibly destroyed by a slope collapse in the region. However, a recent geological expedition to the CGFZ (*R/V A. N. Strakhov* Expedition S50) discovered an abundance of fragments of fossil corals by dredging at 1000 m depths and identified them as solitary *Desmophyllum dianthus* (Skolotnev et al. 2021).

### Cnidarians (corals, sea anemones, cerianthids and hydroids) of the CGFZ

Reports published following the MAR-ECO project have provided details on the coral occurrences for certain regions of the Mid-Atlantic Ridge, mainly between the southern part of the Reykjanes Ridge and the Azores (Mortensen et al. 2008). This MAR-ECO survey collected ROV video as well as trawl samples from sites northwest and southeast of the CGFZ. Only two morphospecies of Antipatharia were reported from the MAR-ECO data compared to 26 morphospecies observed in the current study, including five genera well known to the deep sea ecosystems of the North Atlantic, *Stauropathes*, *Stichopathes*, *Leiopathes*, *Parantipathes* and *Bathypathes*. The only reef-forming scleractinian coral recorded during the TOSCA expedition was *Solenosmilia variabilis*, observed below 1100 m, which aligns with previous studies detailing the depth ranges of *Desmophyllum pertusum* (found no deeper than 1100 m) and *Solenosmilia variabilis* (found no shallower than 1100m) (Henry and Roberts 2014; Howell et al. 2014). With 37 morphospecies from order Alcyonacea and 16 from Pennatulacea, these groups were again found to be much more diverse than previously reported for the region, with only 27 morphospecies within Octocorallia previously reported (Mortensen et al. 2008). ROV video quality has improved since the MAR-ECO expedition (Mortensen et al. 2008), and there is now a larger wealth of online species catalogues to aid in identification. The MAR-ECO surveys also had limited sampling of corals, and more than half (24 out of 41) of the morphospecies observations were made from bycatch on longlines and trawls (Bergstad and Gebruk 2008; Mortensen et al. 2008). However, taking this into account alongside the current regime and the topographical complexity (including a seamount and ridge feature) of the CGFZ, it is possible that the benthic ecosystems between the parallel transform faults of the CGFZ contain a heightened level of biodiversity, specifically species richness, compared to the sites north and south of the CGFZ that were sampled on the MAR-ECO expeditions.

A higher level of coral morphospecies occurrence was found on bedrock, which supports previous studies that found higher species richness for cnidarians on hard substratum on the MAR (Mortensen et al. 2008; Watanabe et al. 2009). Mortensen et al. (2008) found that the number of coral taxa present in their study was strongly correlated with the percentage cover of hard substrates. A more recent study, focussing on similar areas on either side of the CGFZ, found that the species richness of corals increased with the amount of hard substrates, but that the abundance of corals was not correlated with the availability of hard substrata (Bell et al. 2016). Bell et al. (2016) found large areas of bedrock uninhabited by megafauna, a pattern which was observed during Dive 7 of the TOSCA survey. From visual observations, much of Dive 7 appeared to have less marine snow (suspended organic detritus) compared to other dives in the region. There was an obvious lack of life on many of the exposed bedrock and boulder fields, in comparison to the other dives with similar bedrock and boulder substratum. We hypothesise that Dive 7 had less marine snow due to its orientation with respect to the currents in this region, leading to a lack of benthic megafauna which rely on this as their primary food source.

### Sponges of the CGFZ

The dense sponge aggregation of Dive 9 (Figs 3g and 8b) may be an important ecosystem engineer for the CGFZ. Even though deep sea sponge aggregations are not as well known as their shallow water counter parts, certain species have been found to provide important functional roles for other benthic fauna, which includes acting as complex three-dimensional habitats (Beazley et al. 2015; Howell et al. 2016; Maldonado et al. 2017). The sponge aggregation observed on the CGFZ was dominated by demosponges and may be referred to as an ‘ostur’ or ‘cheese-bottom’, which is a term coined by Klitgaard and Tendal (2004) and defined as “a restricted area where large-sized sponges are strikingly common” (Klitgaard and Tendal 2004). They described what is known as a “boreal ostur”, which occurs in areas including the Faroe Islands, Norway, Sweden, parts of the western Barents Sea and south of Iceland. Similar sponge ground compositions have been recorded on the Flemish Cap and the Grand Banks of Newfoundland in the Northwest Atlantic, including multiple species of *Geodia* sp. with encrusting Demosponge epibionts (Murillo et al. 2012, 2016). These are comparable, in terms of species composition and temperature range, to the osturs in the Northeast Atlantic (Murillo et al. 2016). Considering the geographic location of the TOSCA survey, just south of the Reykjanes Ridge, this sponge aggregation can also be considered as a boreal ostur. The presence of this type of sponge aggregation may also be driving the high levels of biodiversity observed in Dive 9, as the ostur may be acting as an ecosystem engineer, providing a complex three-dimensional habitat. No studies have previously examined the presence of this kind of sponge ground on the CGFZ. The CGFZ has also been marked as a biogeographic transition zone for demosponges of the North Atlantic as numerous species were found to have morphological differences due to limited gene flow between populations north and south of the fracture zone (Cárdenas and Rapp 2015). One study described the variation in demosponge density at sites northeast, northwest, southeast and southwest of the CGFZ and found that the highest densities lay to the north of the fracture, and slightly higher densities, highest being 0.47 sponges per m^2^, again at the Northwest site (Bell et al. 2016). These demosponge densities were, however, much lower than what we recorded, with highest densities of over 6 sponges per square metre in this study. This could have an impact on the level of protection this region of the CGFZ may be granted in the future, as osturs are recognised as ecosystem engineers (Beazley et al. 2015).

### Xenophyophores

The presence of large aggregations of the giant protists, Xenophyophores, on gently sloping sandy areas was notable as previous studies have found them to provide for refuge, feeding and mating sites for other deep sea species (Gooday et al. 1992; Levin et al. 1991; Levin and Rouse 2020). Their morphology is adapted specifically to trap particles from the water column to form their tests or to feed on (Levin et al. 1991). This in turn makes them attractive dwellings for small invertebrates and they have been described as biodiversity hotspots when found in large aggregations on sediments (Gooday 1986; Levin et al. 1991). A recent study has even revealed that Xenophyophores can act as fish nurseries (Levin and Rouse 2020). Xenophyophores were numerous in individual abundances (with densities of up to 6 individuals per m^2^), despite only one morphospecies being identified, and they are expected to play a functional role in habitat provisions for the sandy regions of the CGFZ.

Based on results from the HGAM and species accumulation curves, the lowest diversity and richness was recorded in sandy, gently sloping regions of the CGFZ, but Xenophyophores likely harbour high levels of diversity within their structures (Gooday 1986; Levin et al. 1991) that is difficult to observe from ROV video alone. Therefore, future research in this region should sample Xenophyophores and macrofauna to better understand the taxonomic diversity and their role in structuring the biodiversity at the CGFZ.

### Possible anthropogenic stressors on the benthic environment of the CGFZ

Evidence of anthropogenic stress was observed in the CGFZ and was especially evident during the Hecate seamount dive (Dive 8) which spanned a depth range of 2340 m from the start point of 2900 m to its peak at 560 m. A large fishing net was observed on the seamount at 800 m depth on a relatively steeply sloping bedrock, overlain with sand in some regions (see supplementary material for images). The net appeared relatively new, with little to no biofouling, and it was observed close to large patches of dead hexactinellid sponges. It is uncertain whether the dead sponges were due to the impacts of previous trawl fishing, slope collapses on the seamount or a combination of both factors. In addition to this, five glass bottles and a plastic bag were observed on the seamount, in comparison to only one bottle observed on Dive 5, a cable on Dive 6, and one cable observed on Dive 9. At the seamount’s peak, the endangered species of fish, *Hoplostethus atlanticus* (also known as Orange Roughy, see species catalogue in supplementary material) was observed. In the past, extensive Orange Roughy fisheries were conducted on the MAR, but these have since declined due to overexploitation and subsequent management by NEAFC and the EU (Bergstad 2016). There is still a small fishery for Orange Roughy being conducted on Faraday Seamount by the Faroe Islands as per the OSPAR (Convention for the Protection of the Marine Environment of the Northeast Atlantic) report on Seamounts (Kutti et al. 2019), but none are reported for the Hecate Seamount. Seamounts in the OSPAR maritime area are presumed to function as nurseries, feeding and spawning areas for a number of commercially and ecologically important deep sea fish species (Hareide and Garnes 2001; Kutti et al. 2019). Regulating fishing activity where VMEs are present such as this site is important to preserve these ecosystem functions.

## Conclusions

The CGFZ is highly biologically diverse, as well as having morphologically complex bathymetry. The biodiversity and spatial distribution of ecologically important megafaunal groups on the CGFZ are potentially driven by multiple environmental factors including substrate type slope and depth gradients. Higher levels of biodiversity were found in areas dominated by bedrock and within three 250 m depth bands (between 1500 and 2549 m). Dive 9, which ran along a ridge feature, proved to be an important sponge habitat. This study will help to direct ecologically driven sampling efforts on the CGFZ in the future for a better understanding of the rare and vulnerable species that are present. The confirmed presence of a boreal ostur, coral gardens, xenophyophore aggregations and other seamount species (Orange Roughy as an example) are important observations as the protection status of this region of the CGFZ will come into debate in the coming years. This study provides a detailed insight into the megafaunal biodiversity, its spatial variation, and their potential environmental drivers within the CGFZ North MPA, which is still only partially protected, leaving the seafloor vulnerable to exploitation. In conjunction with this study, morphospecies observations derived from video analysis were submitted to the ICES VME data call 2021 to be added to a database on the deep sea ecosystems of the North Atlantic. We suggest this species data and biodiversity descriptions should be used in the future decisions made when reviewing the protection of this remote and topographically unique region of the North Atlantic.

## Supplementary information

ESM 1 (DOCX 24205 kb)

## Declarations

### Sampling and field studies

All necessary permits for sampling and observational field studies have been obtained by the authors from the competent authorities and are mentioned in the acknowledgements. The study is compliant with CBD and Nagoya protocols.

### Data availability

The datasets generated during and/or analysed during the current study are available from the corresponding author on reasonable request and will shortly be openly available on the Pangea, Data Publisher for Earth & Environmental Science 10.1594/PANGAEA.946727.
