# Supplementary material for: Benthic megafaunal biodiversity of the Charlie-Gibbs fracture zone: spatial variation, potential drivers, and conservation status
Source: Mar Biodivers. 2022 Sep 26;52(5):55. doi: 10.1007/s12526-022-01285-1 (PMC9512888; doi:10.1007/s12526-022-01285-1)
Supplement: Supplementary file 1 — (DOCX 24205 kb) [file 12526_2022_1285_MOESM1_ESM.docx]

**Benthic megafaunal biodiversity of the Charlie-Gibbs Fracture Zone: spatial variation, potential drivers, and conservation status.**

Poppy Keogh^1,2^, Rylan J. Command^2^, Evan Edinger^1^, Aggeliki Georgiopoulou^3^, Katleen Robert^2^.

^1^Geography Department, Memorial University of Newfoundland and Labrador.

^2^Fisheries and Marine Institute, Memorial University of Newfoundland and Labrador.

^3^School of Environment and Technology, University of Brighton.

**Corresponding author:** Poppy Keogh; pkeogh@mun.ca

Supplementary material


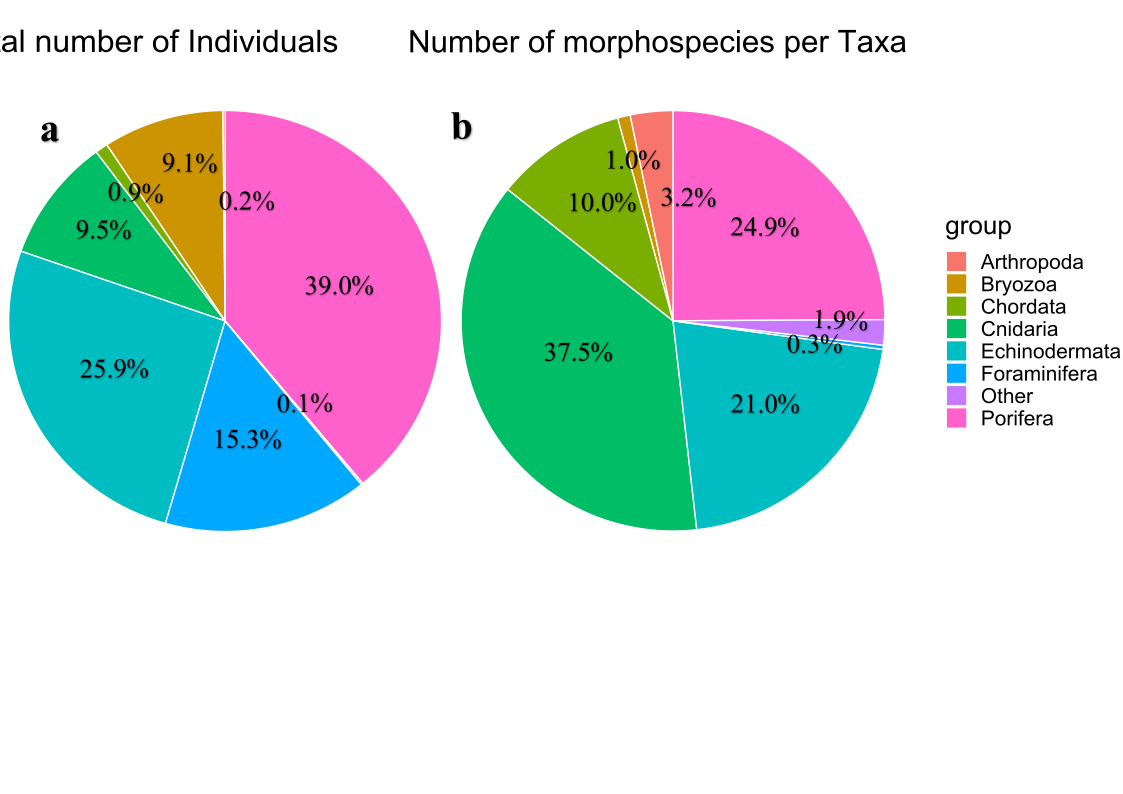


Figure 1. Pie charts showing (a) the proportions of individual organisms observed per Phylum and (b) the number of morphospecies per Phylum present in all ROV dives from the TOSCA survey. “Foraminifera” includes one morphospecies of Xenophyophore. “Other” includes Mollusca (Decapodiformes and Bivalvia) and Annelida (Bonellia sp., Sabellidae). Total number of individuals observed (n=154,509) and total number of morphospecies recorded (z=309).


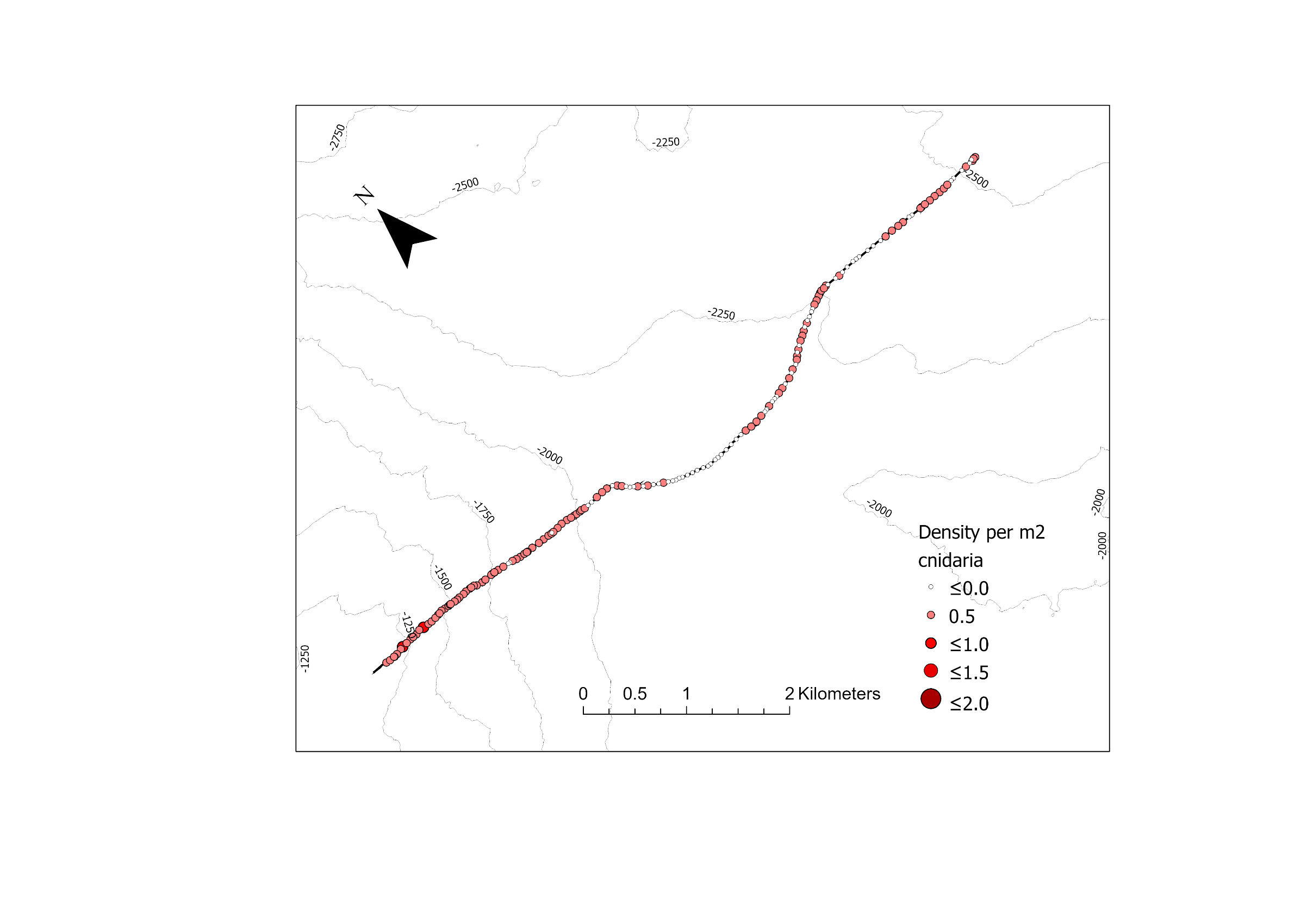


Figure 2. Bubble transect plot showing coral densities on Dive 5. Each circle represents coral observations for a 50 m section of the transect. White circles represent a 50 m section with no observations. No circles present represent the sections of the transect where visibility was too poor to annotate. These sections were removed from the analysis. Density values refer to number of corals per m2. Same details apply for all transect plots.


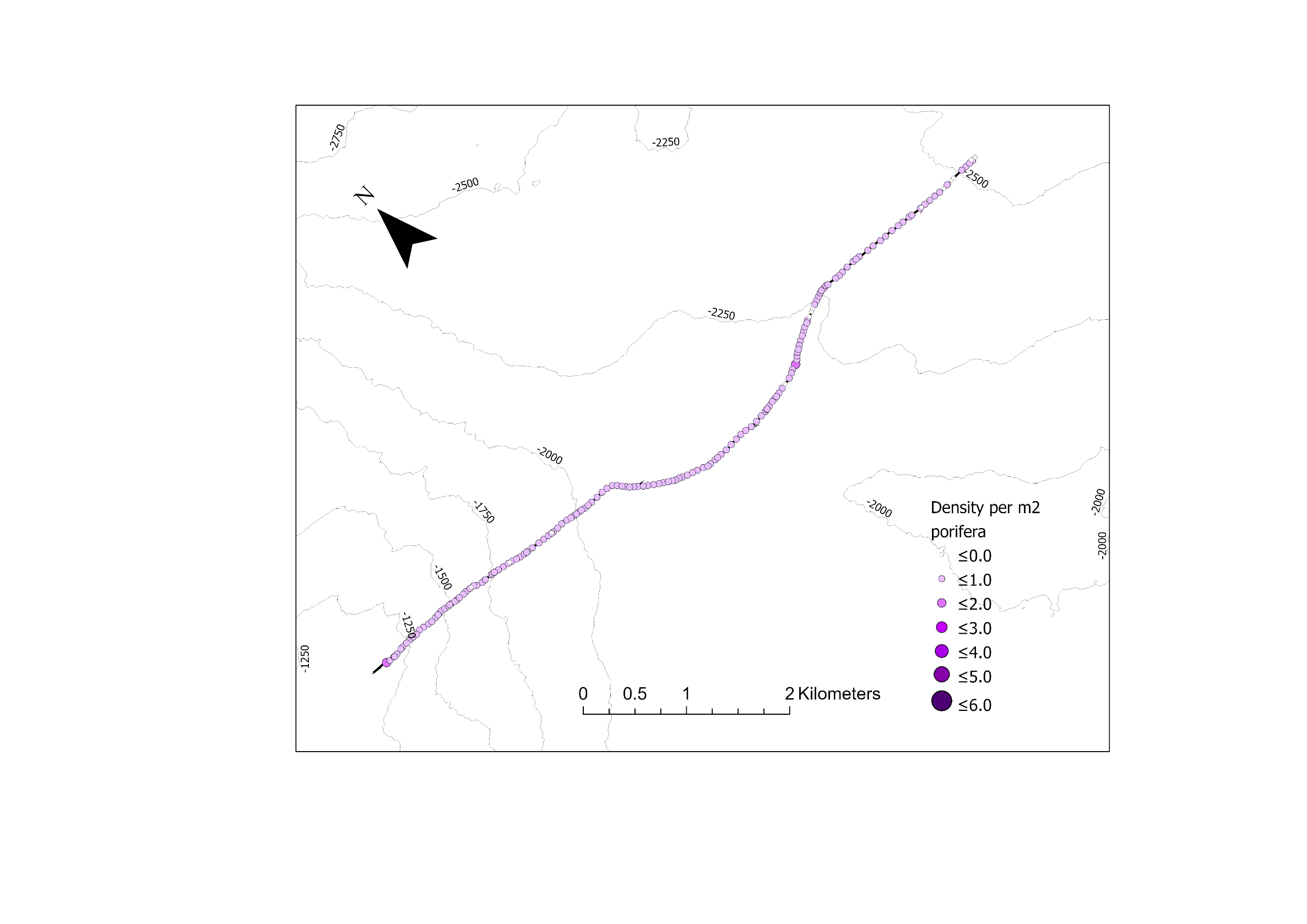


Figure 3. Dive 5 Porifera density.


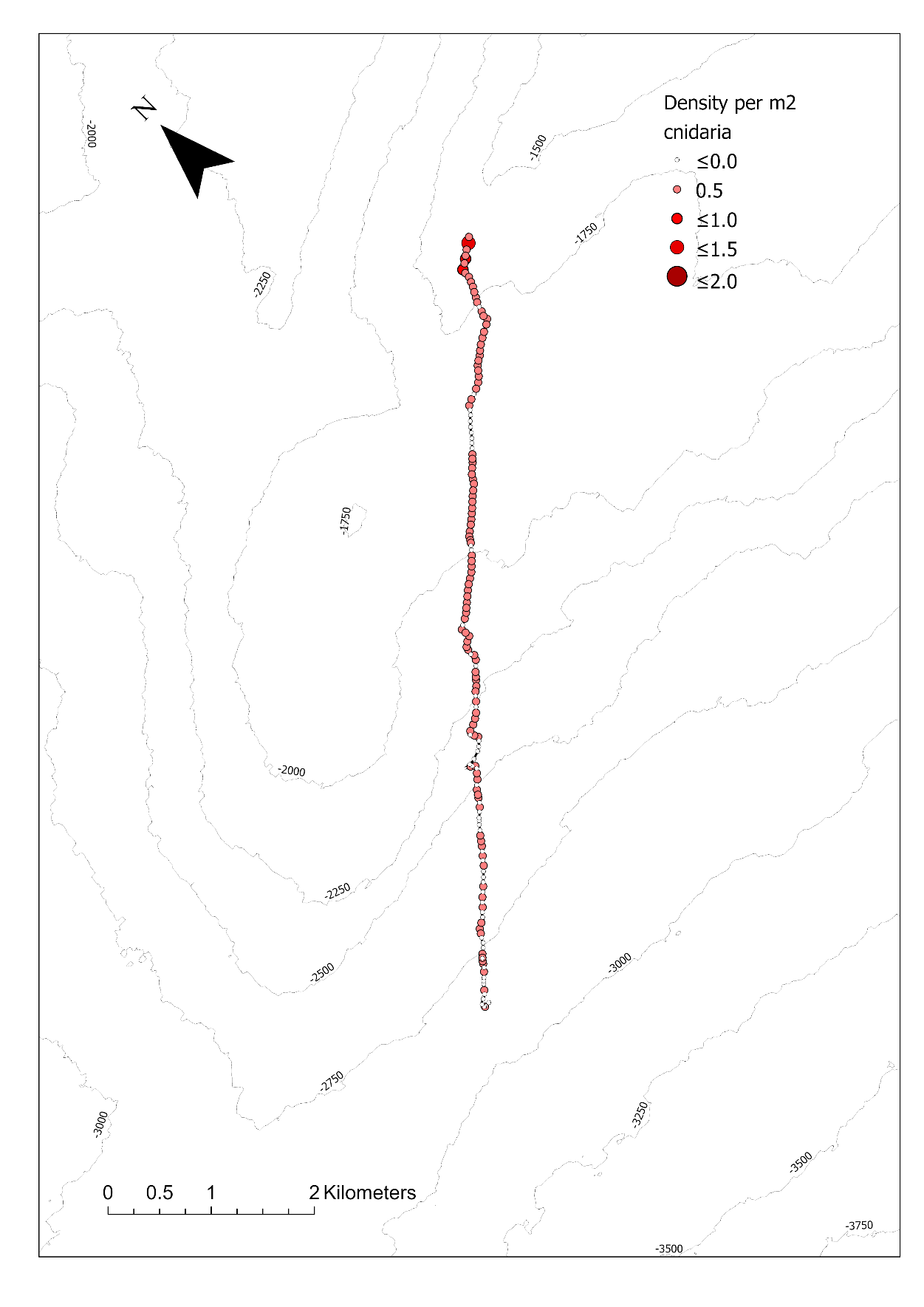


Figure 4. Dive 6 Cnidaria density.


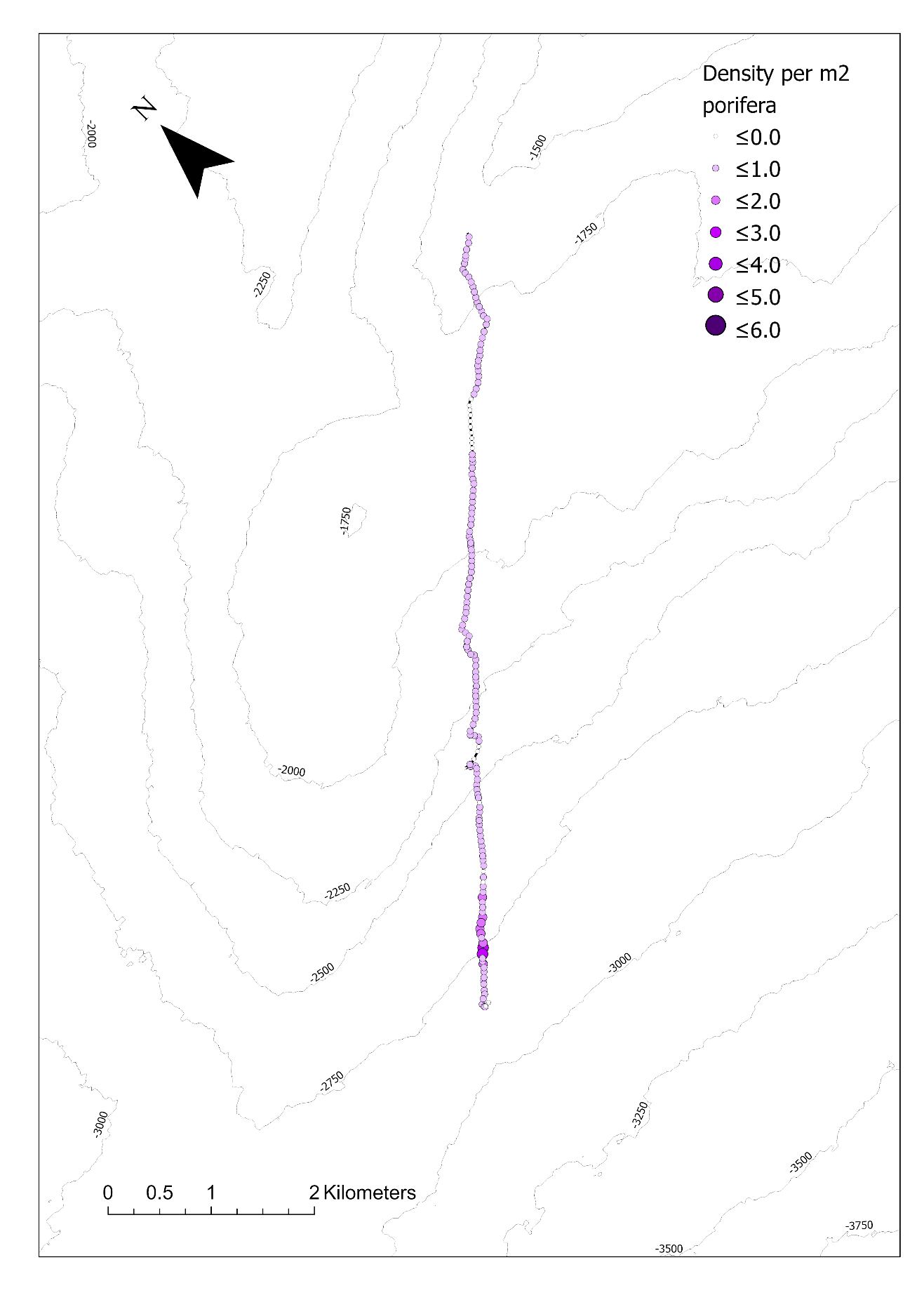


Figure 5. Dive 6 Porifera density.


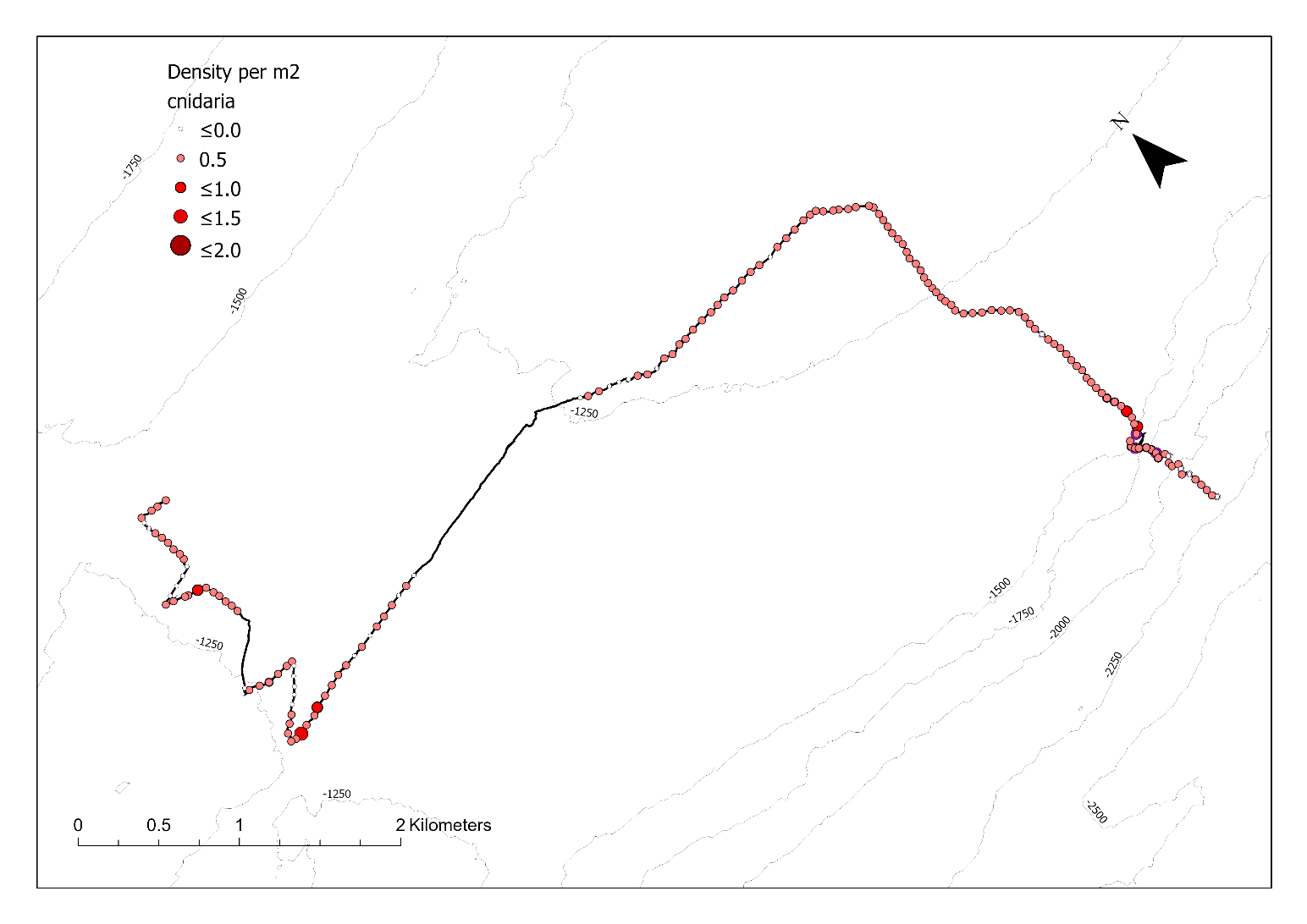


Figure 6. Dive 7 Cnidaria density.


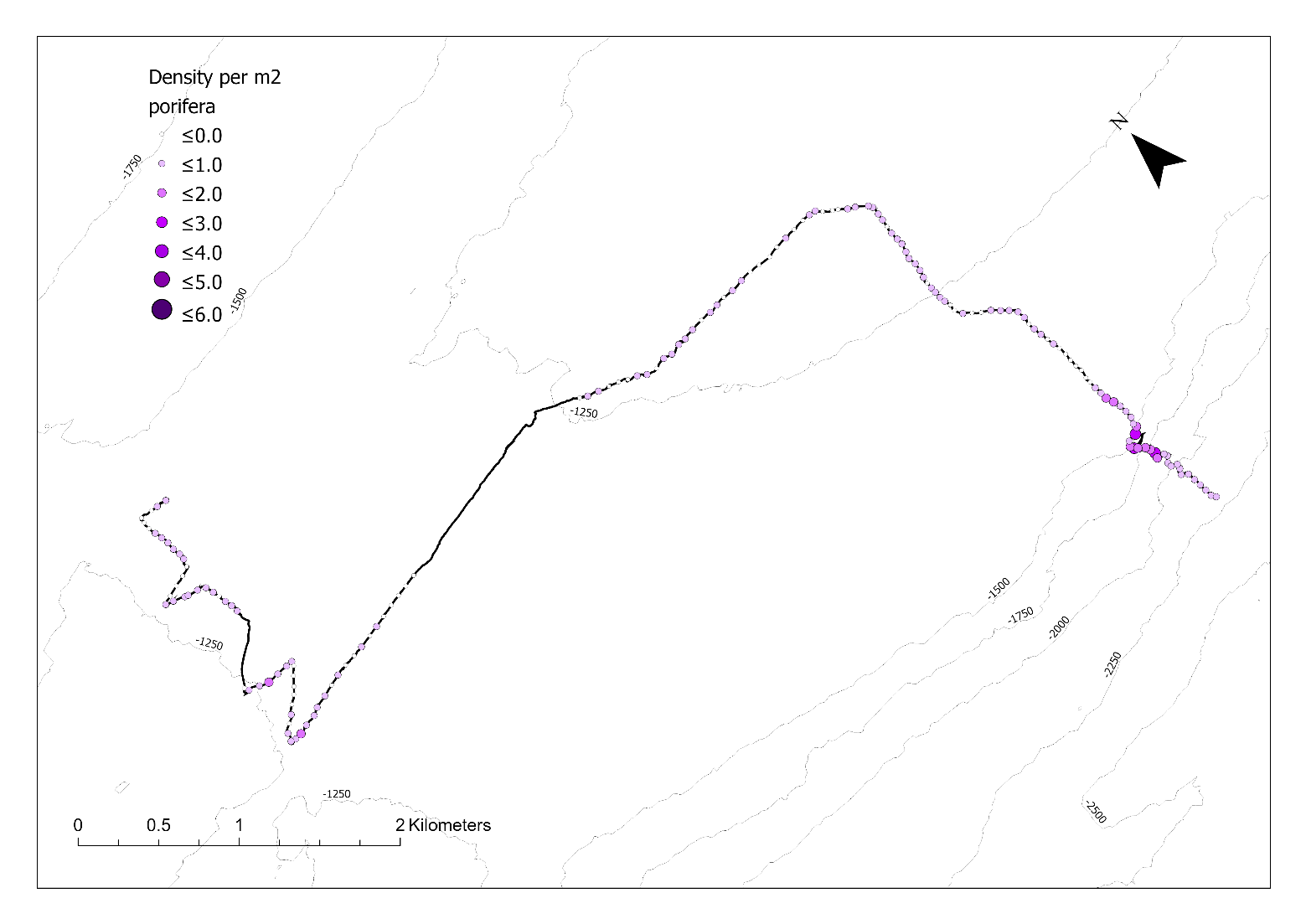


Figure 7. Dive 7 Porifera density.

**
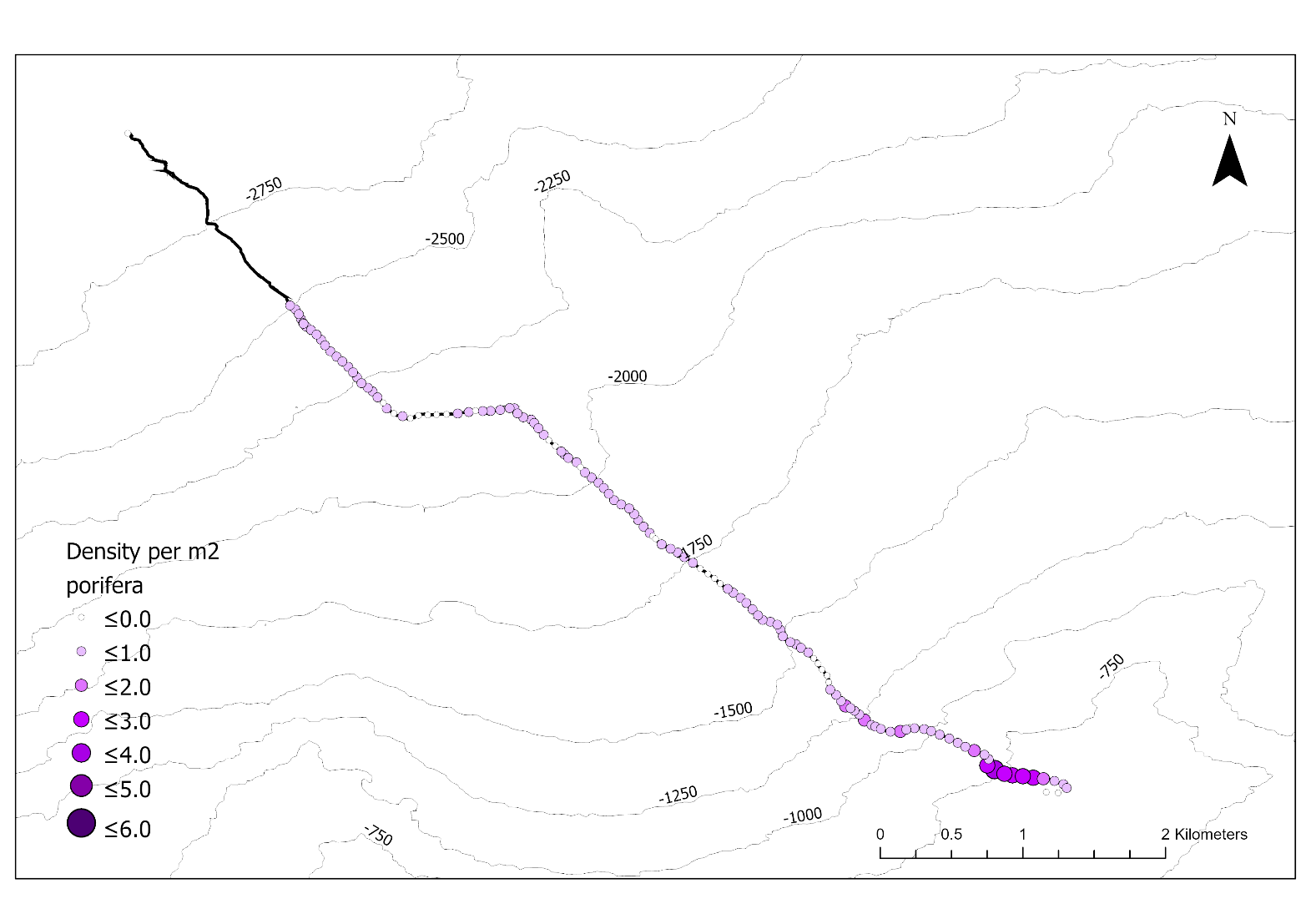
**

Figure 8. Dive 8 Porifera density.


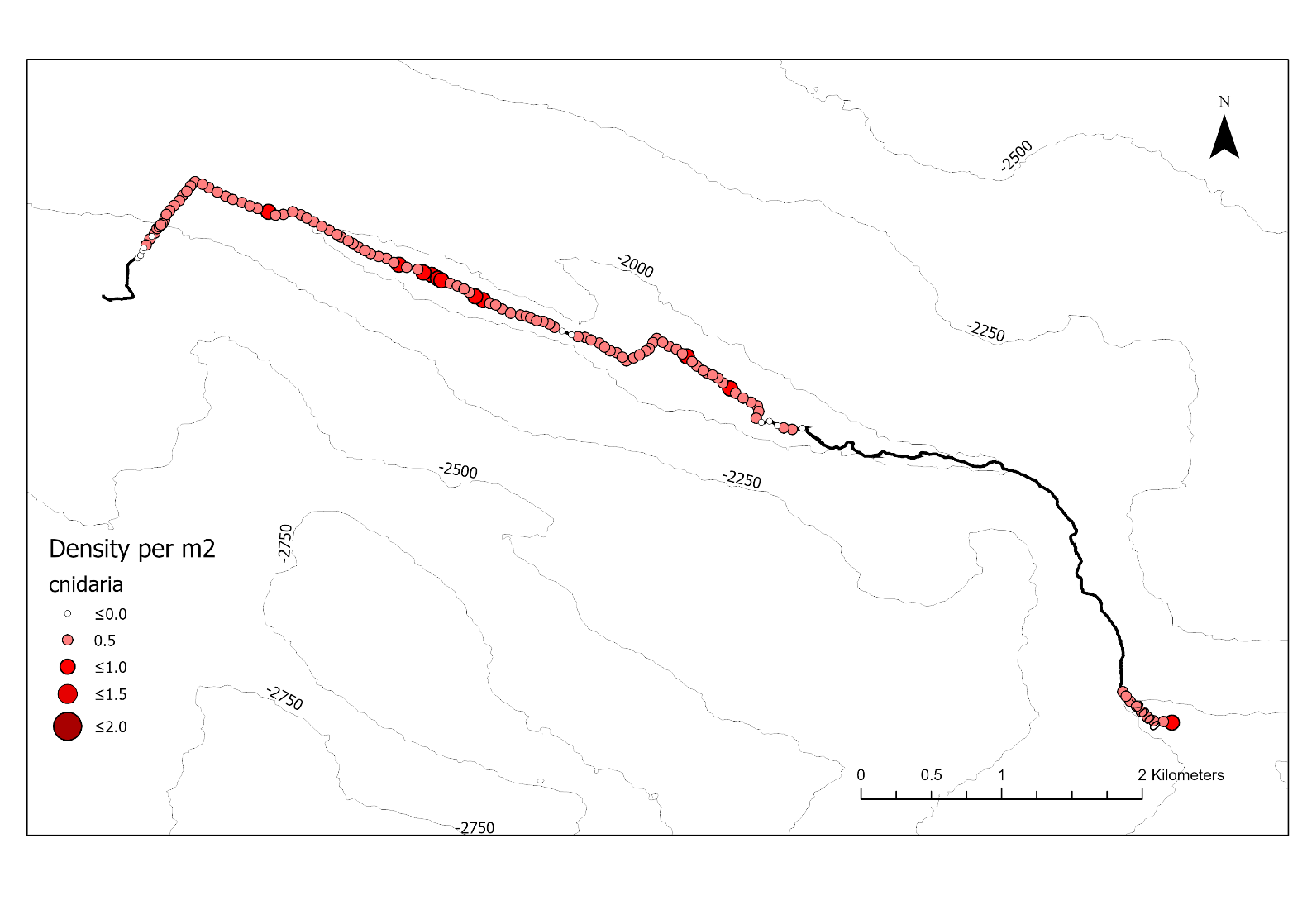


Figure 9. Dive 9 Cnidaria density.


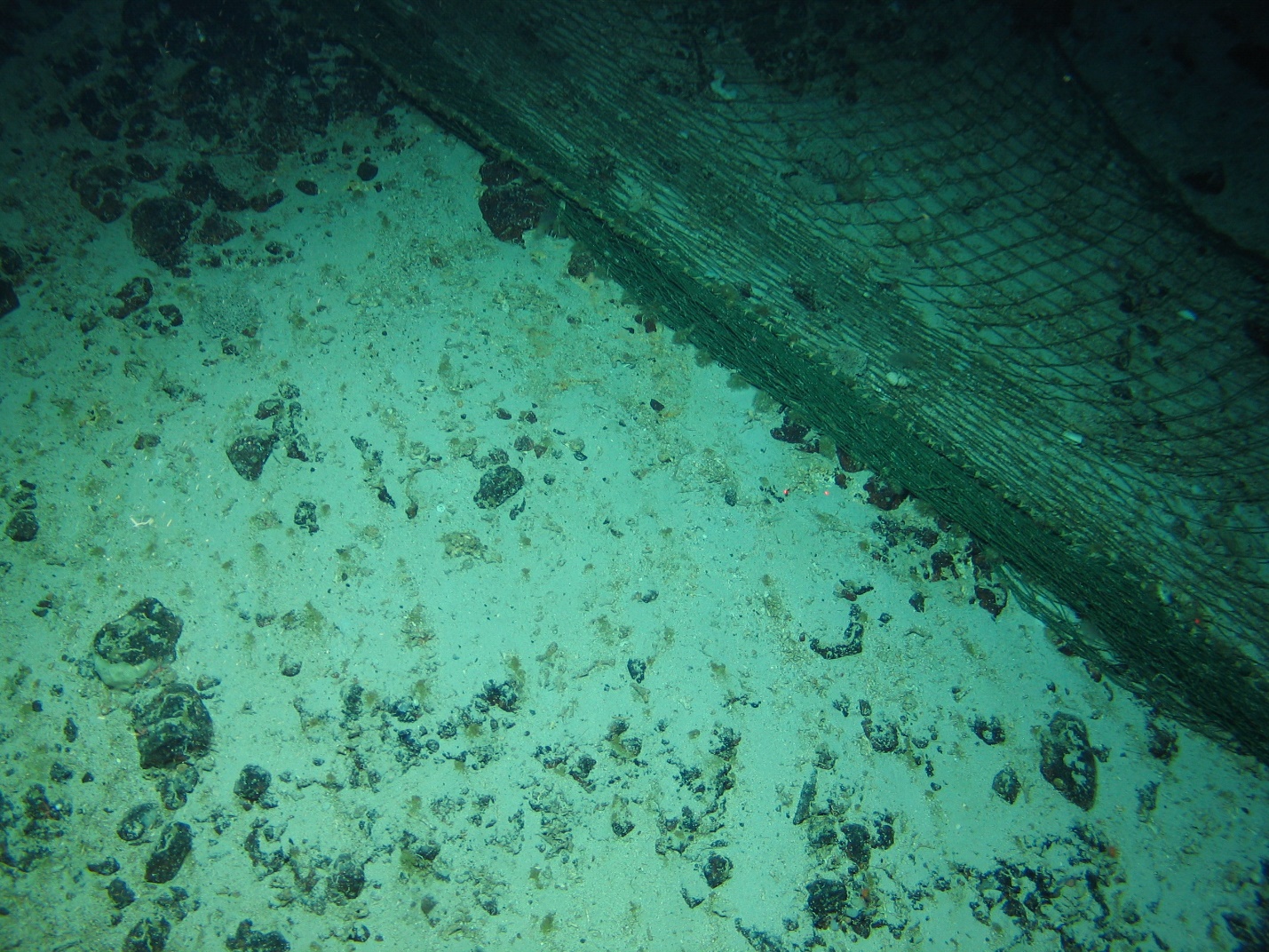


Figure 10. Trawl net on Hecate seamount (Dive 8).


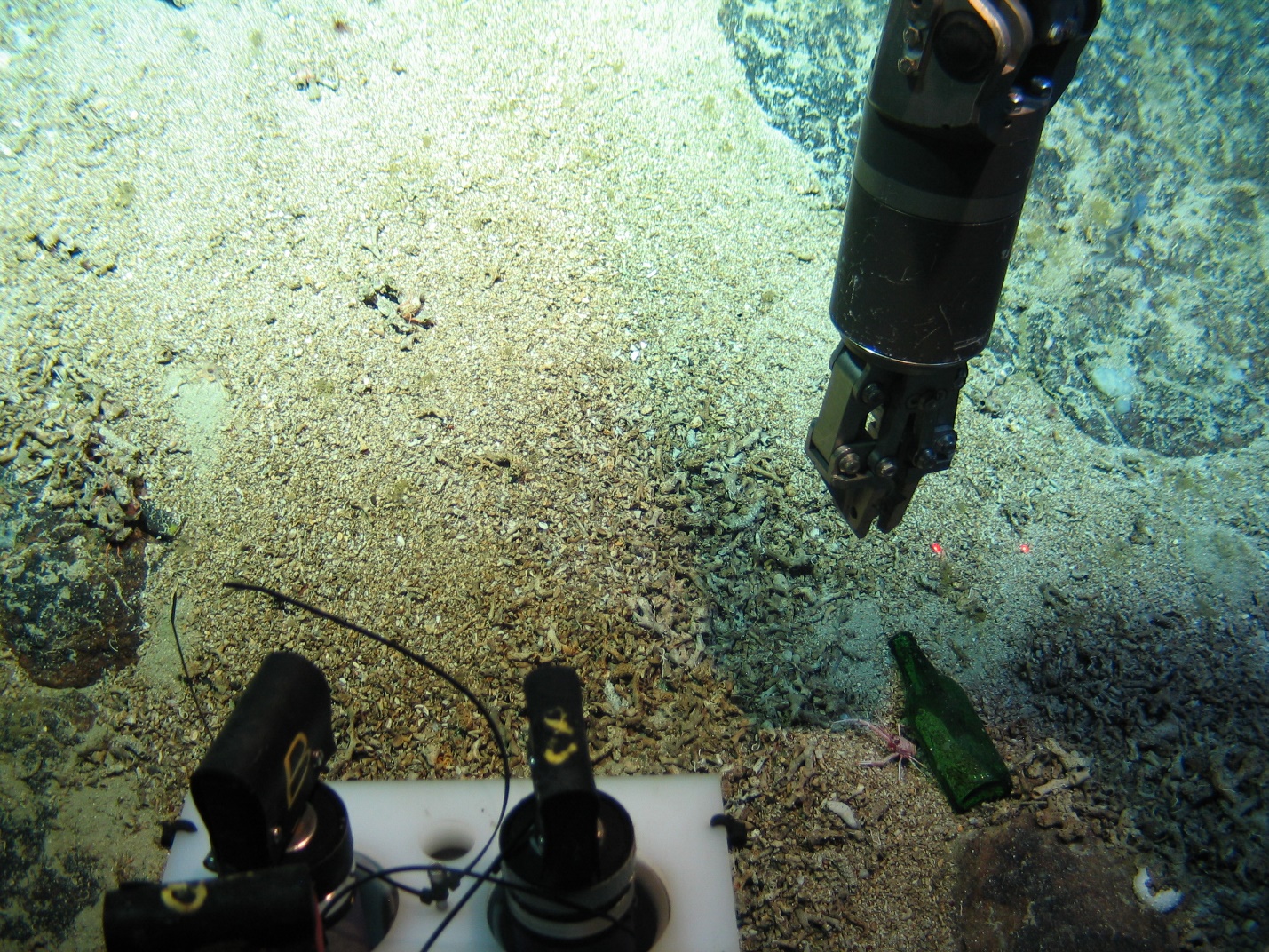


Figure 11. Bottle found on Hecate seamount (Dive 8)

**Species Catalogue – Charlie-Gibbs Fracture Zone**

In this catalogue, all observed morphospecies from the TOSCA (Tectonic Ocean Spreading at the Charlie-Gibbs Fracture Zone) survey are compiled as a reference guide. Information on the taxonomic status are given as well as the species names when applicable. Names of the taxonomic experts identifying the species are given for each species. All morphospecies were observed by the ROV *Holland I* video camera along five ROV transects (indicated by dive number in the catalogue), with a high-definition oblique-facing camera. No physical samples that were collected on the TOSCA survey were documented here, only morphospecies observed from the video.

| **TAXONOMY** | **NAME** | **HABITAT** | **NOTES** | **IMAGE** |
| --- | --- | --- | --- | --- |
| **Phylum:**  Porifera  **Class:**  Demospongiae  **Order:**  **Family:** | **Morphospecies code:** SPONGE2 | **Gear:**  ROV  **Photo by:**  *Holland I* 2018  **Identified by:**  J. Xavier  **Dive: 8** |  | 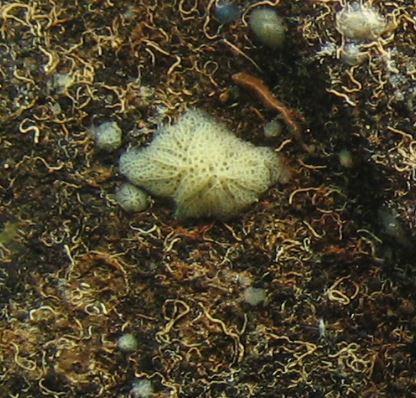 |
| **Phylum:**  Porifera  **Class:**  **Order:**  **Family:** | **Morphospecies code:** SPONGE5 | **Gear:**  ROV  **Photo by:**  *Holland I* 2018  **Identified by:**  J. Xavier  **Dive: 5** |  | 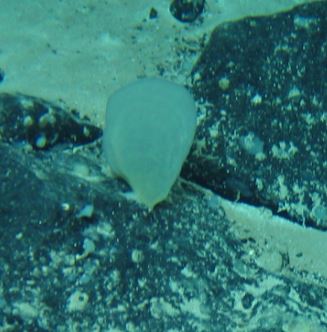 |
| **Phylum:**  Porifera  **Class:**  Demospongiae  **Order:**  **Family:** | **Morphospecies code:** SPONGE6 | **Gear:**  ROV  **Photo by:**  *Holland I* 2018  **Identified by:**  J. Xavier  **Dive: 5** |  | 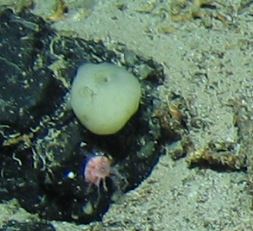 |
| **Phylum:**  Porifera  **Class:**  Demospongiae  **Order:**  **Family:** | **Morphospecies code:** SPONGE11 | **Gear:**  ROV  **Photo by:**  *Holland I* 2018  **Identified by:**  J. Xavier  **Dive: 7** | Possibly Cladorhizidae (J. Xavier) | 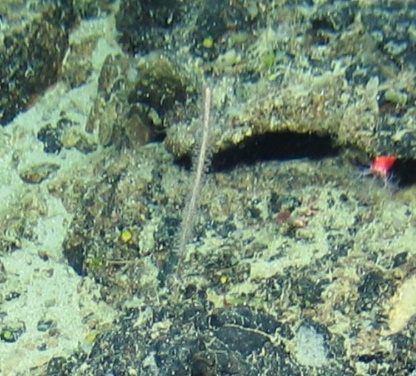 |
| **Phylum:**  Porifera  **Class:**  Demospongiae  **Order:**  **Family:** | **Morphospecies code:** SPONGE12 | **Gear:**  ROV  **Photo by:**  *Holland I* 2018  **Identified by:**  J. Xavier  **Dive: 5** |  | 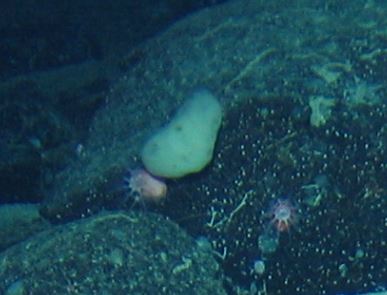 |
| **TAXONOMY** | **NAME** | **HABITAT** | **NOTES** | **IMAGE** |
| **Phylum:**  Porifera  **Class:**  Demospongiae  **Order:**  **Family:** | **Morphospecies code:** SPONGE13 | **Gear:**  ROV  **Photo by:**  *Holland I* 2018  **Identified by:**  J. Xavier  **Dive: 5** |  | **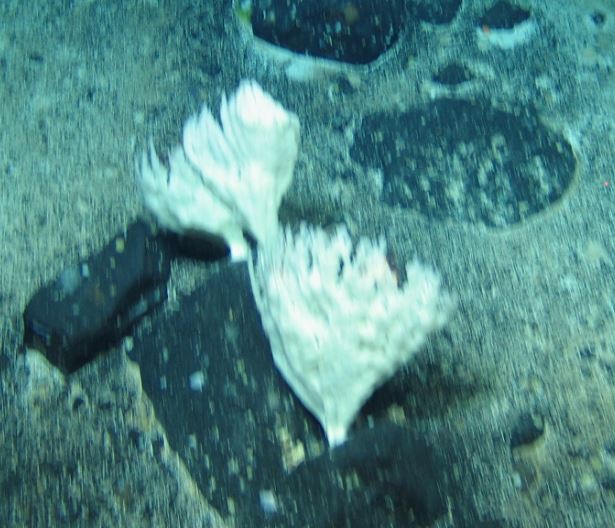** |
| **Phylum:**  Porifera  **Class:**  Demospongiae  **Order:**  **Family:** | **Morphospecies code:** SPONGE14 | **Gear:**  ROV  **Photo by:**  *Holland I* 2018  **Identified by:**  J. Xavier  **Dive: 7** |  | **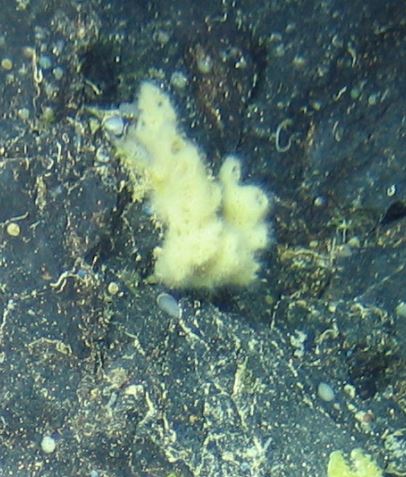** |
| **Phylum:**  Porifera  **Class:**  Demospongiae  **Order:**  **Family:** | **Morphospecies code:** SPONGE17 | **Gear:**  ROV  **Photo by:**  *Holland I* 2018  **Identified by:**  J. Xavier  **Dive: 5** |  | **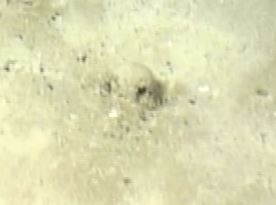** |
| **Phylum:**  Porifera  **Class:**  **Order:**  **Family:** | **Morphospecies code:** SPONGE18 | **Gear:**  ROV  **Photo by:**  *Holland I* 2018  **Identified by:**  **Dive: 8** |  | **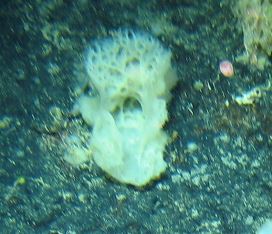** |
| **Phylum:**  Porifera  **Class:**  **Order:**  **Family:** | **Morphospecies code:** SPONGE20 | **Gear:**  ROV  **Photo by:**  *Holland I* 2018  **Identified by:**  **Dive: 6** |  | **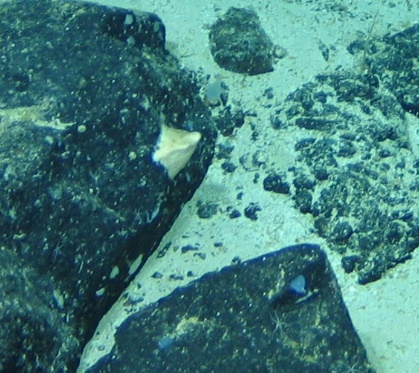** |
| **TAXONOMY** | **NAME** | **HABITAT** | **NOTES** | **IMAGE** |
| **Phylum:**  Porifera  **Class:**  **Order:**  **Family:** | **Morphospecies code:** SPONGE21 | **Gear:**  ROV  **Photo by:**  *Holland I* 2018  **Identified by:**  **Dive: 8** |  | **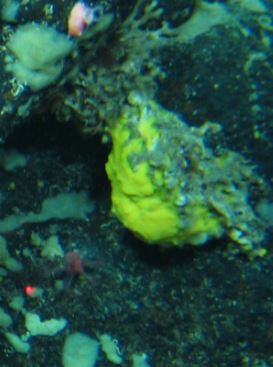** |
| **Phylum:**  Porifera  **Class:**  **Order:**  **Family:** | **Morphospecies code:** SPONGE22 | **Gear:**  ROV  **Photo by:**  *Holland I* 2018  **Identified by:**  **Dive: 9** |  | **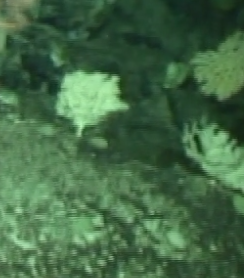** |
| **Phylum:**  Porifera  **Class:**  **Order:**  **Family:** | **Morphospecies code:** SPONGE23 | **Gear:**  ROV  **Photo by:**  *Holland I* 2018  **Identified by:**  **Dive: 6** |  | **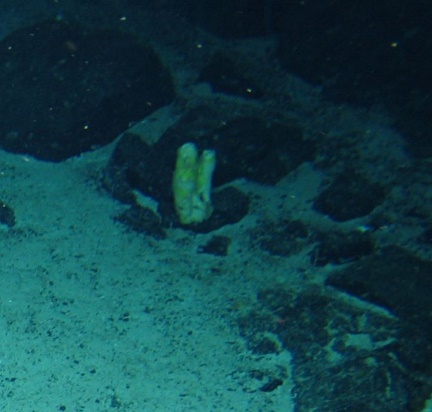** |
| **Phylum:**  Porifera  **Class:**  Demospongiae  **Order:**  **Family:** | **Morphospecies code:** ENCR1 | **Gear:**  ROV  **Photo by:**  *Holland I* 2018  **Identified by:**  J. Xavier  **Dive: 5** |  | **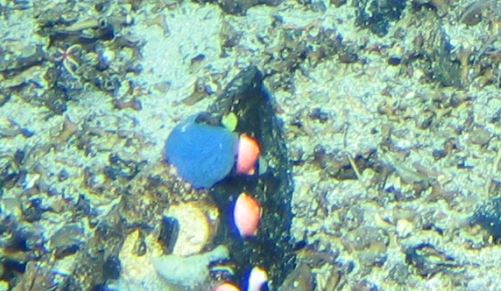** |
| **Phylum:**  Porifera  **Class:**  Demospongiae  **Order:**  **Family:** | **Morphospecies code:** ENCR3 | **Gear:**  ROV  **Photo by:**  *Holland I* 2018  **Identified by:**  J. Xavier  **Dive: 7** |  | **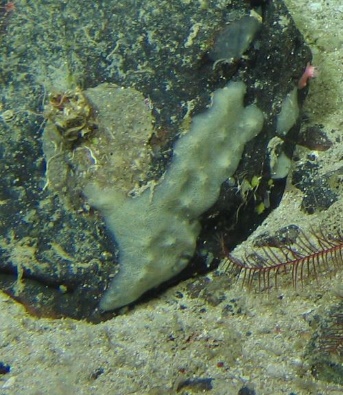** |
| **TAXONOMY** | **NAME** | **HABITAT** | **NOTES** | **IMAGE** |
| **Phylum:**  Porifera  **Class:**  Demospongiae  **Order:**  **Family:** | **Morphospecies code:** ENCR4 | **Gear:**  ROV  **Photo by:**  *Holland I* 2018  **Identified by:**  J. Xavier  **Dive: 7** |  | **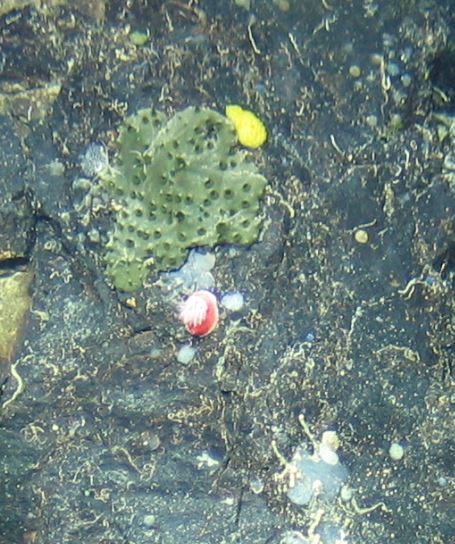** |
| **Phylum:**  Porifera  **Class:**  Demospongiae  **Order:**  **Family:** | **Morphospecies code:** ENCR5 | **Gear:**  ROV  **Photo by:**  *Holland I* 2018  **Identified by:**  J. Xavier  **Dive: 7** |  | **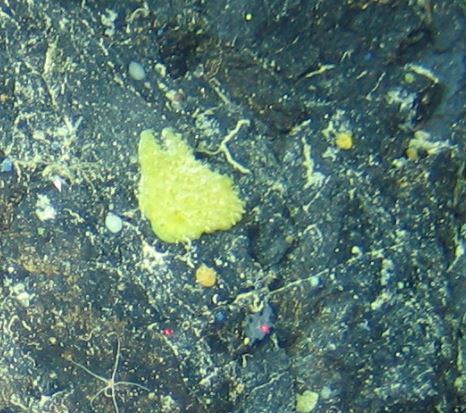** |
| **Phylum:**  Porifera  **Class:**  Demospongiae  **Order:**  **Family:** | **Morphospecies code:** ENCR6 | **Gear:**  ROV  **Photo by:**  *Holland I* 2018  **Identified by:**  J. Xavier  **Dive: 6** |  | **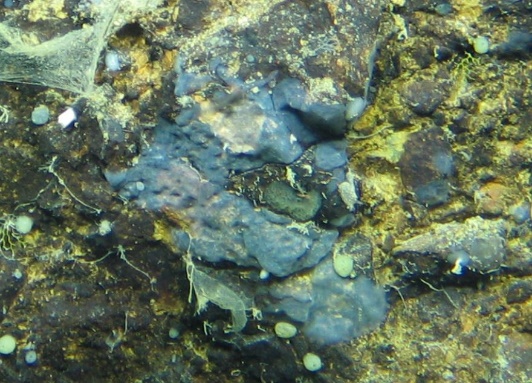** |
| **Phylum:**  Porifera  **Class:**  Demospongiae  **Order:**  **Family:** | **Morphospecies code:** ENCR8 | **Gear:**  ROV  **Photo by:**  *Holland I* 2018  **Identified by:**  J. Xavier  **Dive: 5** |  | **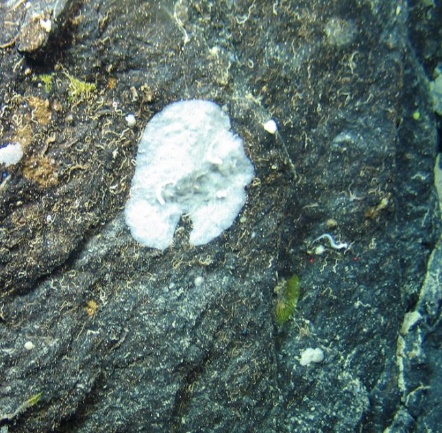** |
| **Phylum:**  Porifera  **Class:**  Demospongiae  **Order:**  **Family:** | **Morphospecies code:** ENCR9 | **Gear:**  ROV  **Photo by:**  *Holland I* 2018  **Identified by:**  J. Xavier  **Dive: 5** |  | **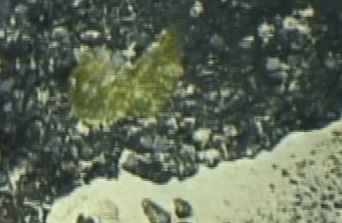** |
| **TAXONOMY** | **NAME** | **HABITAT** | **NOTES** | **IMAGE** |
| **Phylum:**  Porifera  **Class:**  Demospongiae  **Order:**  **Family:** | **Morphospecies code:** ENCR10 | **Gear:**  ROV  **Photo by:**  *Holland I* 2018  **Identified by:**  J. Xavier  **Dive: 8** |  | **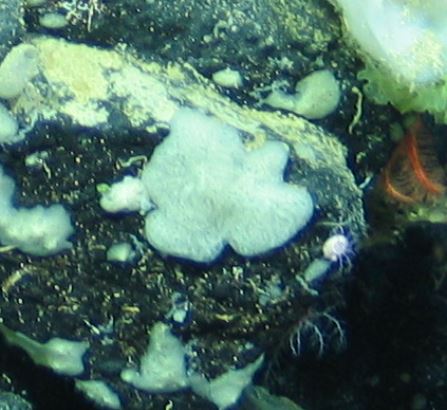** |
| **Phylum:**  Porifera  **Class:**  **Order:**  **Family:** | **Morphospecies code:** ENCR12 | **Gear:**  ROV  **Photo by:**  *Holland I* 2018  **Identified by:**  Poppy Keogh  **Dive: 5** |  | **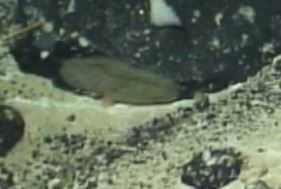** |
| **Phylum:**  Porifera  **Class:**  Demospongiae  **Order:**  **Family:** | **Morphospecies code:** DEMOS3 | **Gear:**  ROV  **Photo by:**  *Holland I* 2018  **Identified by:**  J. Xavier  **Dive: 6** | **Could be a Tetractinellida but difficult to say (J. Xavier)** | **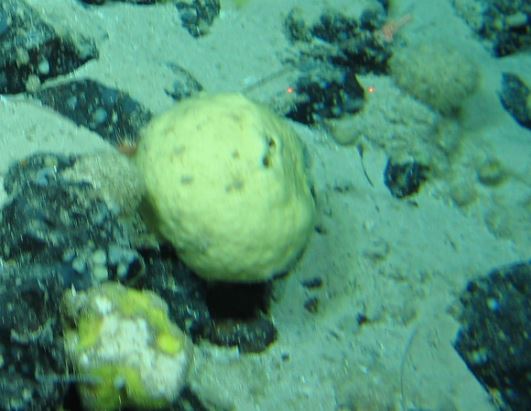** |
| **Phylum:**  Porifera  **Class:**  Demospongiae  **Order:**  **Family:**  **Genus:** | **Morphospecies code:** DEMOS5 | **Gear:**  ROV  **Photo by:**  *Holland I* 2018  **Identified by:**  J. Xavier  **Dive: 5** | Stelletta or Geodia sp. | **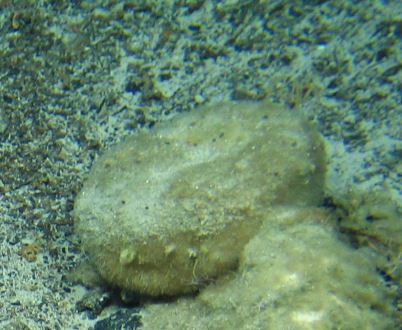** |
| **Phylum:**  Porifera  **Class:**  Demospongiae  **Order:**  **Family:**  **Genus:** | **Morphospecies code:** DEMOS6 | **Gear:**  ROV  **Photo by:**  *Holland I* 2018  **Identified by:**  J. Xavier  **Dive: 6** | Stelletta or Geodia sp. | **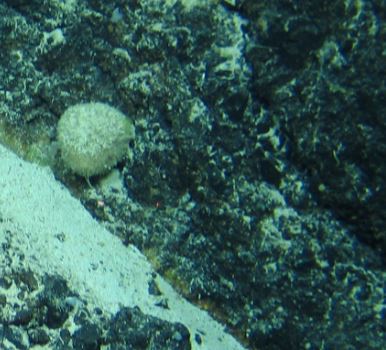** |
| **TAXONOMY** | **NAME** | **HABITAT** | **NOTES** | **IMAGE** |
| **Phylum:**  Porifera  **Class:**  Demospongiae  **Order:** Tetractinellida  **Family:** Geodiidae  **Genus:** *Geodia*  **Species:** *G. phlegraei* | **Morphospecies code:** DEMOS7  *Geodia phlegraei* | **Gear:**  ROV  **Photo by:**  *Holland I* 2018  **Identified by:**  J. Xavier  **Dive: 6** | (Sollas, 1880) | **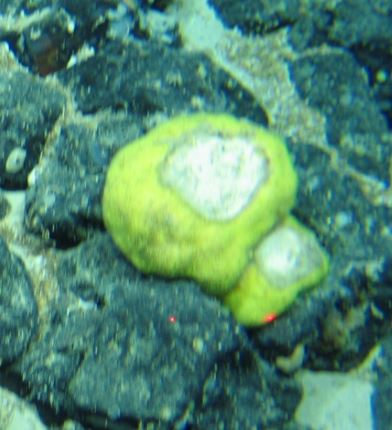** |
| **Phylum:**  Porifera  **Class:**  Demospongiae  **Order:** Tetractinellida  **Family:** Geodiidae  **Genus:** *Geodia*  **Species:** | **Morphospecies code:** DEMOS8  *Geodia* sp. | **Gear:**  ROV  **Photo by:**  *Holland I* 2018  **Identified by:**  J. Xavier  **Dive: 7** |  | **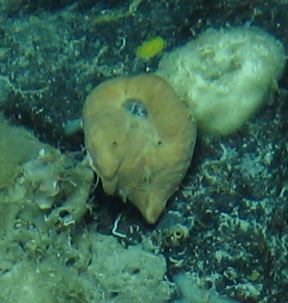** |
| **Phylum:**  Porifera  **Class:**  Demospongiae  **Order:**  **Family:**  **Genus:**  **Species:** | **Morphospecies code:** DEMOS9 | **Gear:**  ROV  **Photo by:**  *Holland I* 2018  **Identified by:**  J. Xavier  **Dive: 5** |  | **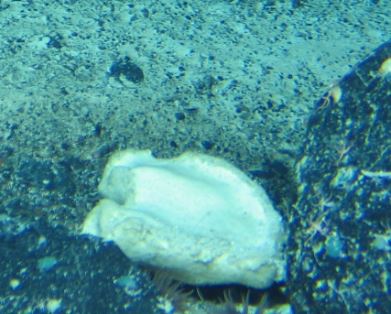** |
| **Phylum:**  Porifera  **Class:**  Demospongiae  **Order:**  **Family:**  **Genus:**  **Species:** | **Morphospecies code:** DEMOS10 | **Gear:**  ROV  **Photo by:**  *Holland I* 2018  **Identified by:**  J. Xavier  **Dive: 7** |  | **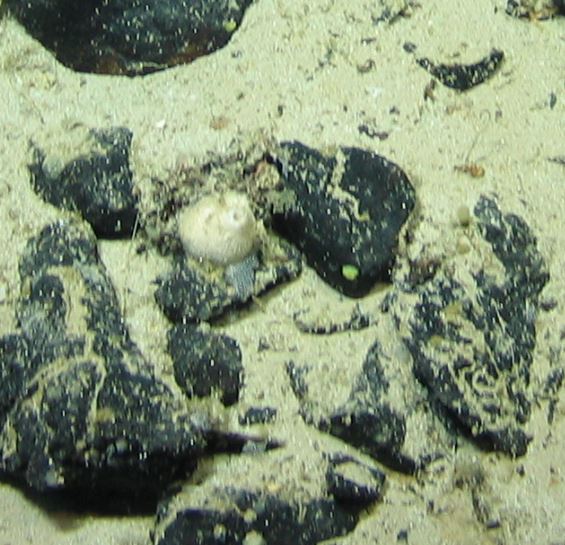** |
| **Phylum:**  Porifera  **Class:**  Demospongiae  **Order:**  **Family:**  **Genus:**  **Species:** | **Morphospecies code:** DEMOS11 | **Gear:**  ROV  **Photo by:**  *Holland I* 2018  **Identified by:**  J. Xavier  **Dive: 5** |  | **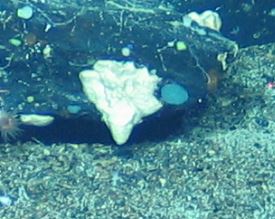** |

| **TAXONOMY** | **NAME** | **HABITAT** | **NOTES** | **IMAGE** |
| --- | --- | --- | --- | --- |
| **Phylum:**  Porifera  **Class:**  Demospongiae  **Order:** Haplosclerida  **Family:** Chalinidae  **Genus:** *Haliclona*  **Species:** *H.* *magna* | **Morphospecies code:** DEMOS12  cf. *Haliclona (Halichoclona) magna* | **Gear:**  ROV  **Photo by:**  *Holland I* 2018  **Identified by:**  J. Xavier  **Dive: 5** | (Vacelet, 1969) | **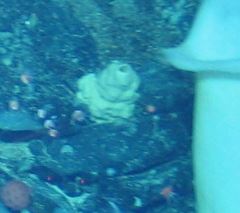** |
| **Phylum:**  Porifera  **Class:**  Demospongiae  **Order:** Tetractinellida  **Family:** Geodiidae  **Genus:** *Geodia*  **Species:** *G. macandrewii* | **Morphospecies code:** DEMOS13  cf. *Geodia macandrewii* | **Gear:**  ROV  **Photo by:**  *Holland I* 2018  **Identified by:**  J. Xavier  **Dive: 5** | (Bowebank, 1858) | **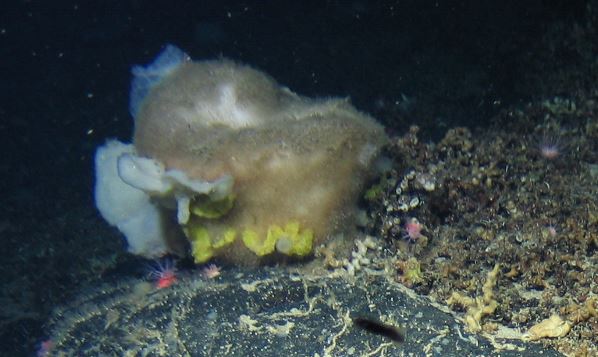** |
| **Phylum:**  Porifera  **Class:**  Demospongiae  **Order:**  **Family:**  **Genus:**  **Species:** | **Morphospecies code:** DEMOS15 | **Gear:**  ROV  **Photo by:**  *Holland I* 2018  **Identified by:**  J. Xavier  **Dive: 6** | Possibly within family Axinellidae but too difficult to say for sure | **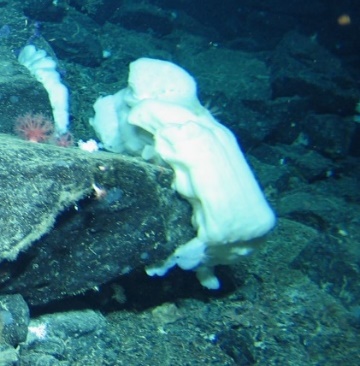** |
| **Phylum:**  Porifera  **Class:**  Demospongiae  **Order:**  **Family:**  **Genus:**  **Species:** | **Morphospecies code:** DEMOS17 | **Gear:**  ROV  **Photo by:**  *Holland I* 2018  **Identified by:**  **Dive: 5** |  | **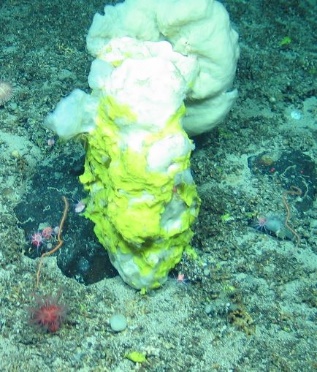** |
| **Phylum:**  Porifera  **Class:**  Demospongiae  **Order:**  **Family:**  **Genus:**  **Species:** | **Morphospecies code:** DEMOS18 | **Gear:**  ROV  **Photo by:**  *Holland I* 2018  **Identified by:**  **Dive: 5** |  | **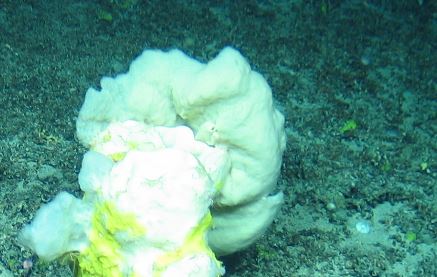** |

| **TAXONOMY** | **NAME** | **HABITAT** | **NOTES** | **IMAGE** |
| --- | --- | --- | --- | --- |
| **Phylum:**  Porifera  **Class:**  Demospongiae  **Order:**  **Family:**  **Genus:** *Geodia*  **Species:** *G. megastrella* | **Morphospecies code:** GEODIA1  *Geodia megastrella* | **Gear:**  ROV  **Photo by:**  *Holland I* 2018  **Identified by:**  J. Xavier  **Dive: 7** | (Carter, 1876) | **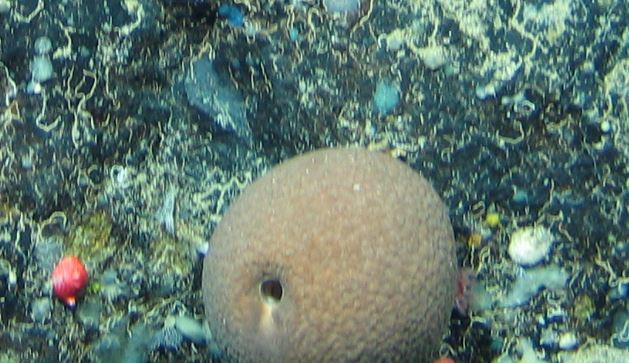** |
| **Phylum:**  Porifera  **Class:**  Demospongiae  **Order:**  **Family:**  **Genus:** *Geodia*  **Species:** *G. megastrella* | **Morphospecies code:** GEODIA2  *Geodia megastrella* | **Gear:**  ROV  **Photo by:**  *Holland I* 2018  **Identified by:**  J. Xavier  **Dive: 5** | (Carter, 1876) | **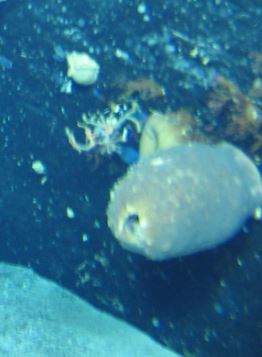** |
| **Phylum:**  Porifera  **Class:**  Demospongiae  **Order:**  **Family:**  **Genus:** *Geodia*  **Species:** | **Morphospecies code:** GEODIA3  *Geodia* sp. | **Gear:**  ROV  **Photo by:**  *Holland I* 2018  **Identified by:**  J. Xavier  **Dive: 5** |  | **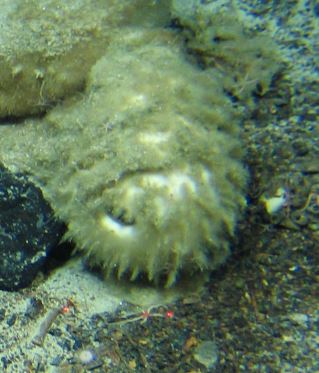** |
| **Phylum:**  Porifera  **Class:**  Demospongiae  **Order:**  **Family:**  **Genus:** *Geodia*  **Species:** *G. hentscheli* | **Morphospecies code:** GEODIA4  cf. *Geodia hentscheli* | **Gear:**  ROV  **Photo by:**  *Holland I* 2018  **Identified by:**  J. Xavier  **Dive: 6** | (Cárdenas, Rapp, Schander & Tendal, 2010) | **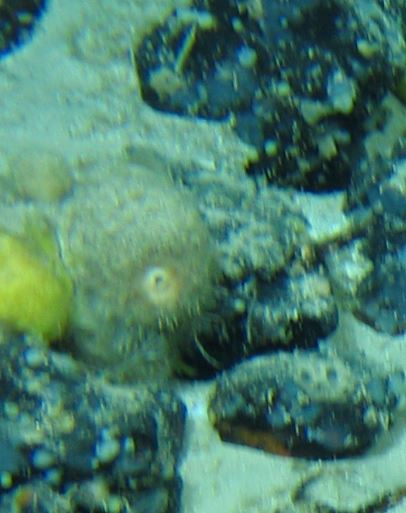** |
| **Phylum:**  Porifera  **Class:**  Demospongiae  **Order:**  **Family:**  **Genus:** *Polymastia*  **Species:** P. *corticata* | **Morphospecies code:** POLYM1  cf. *Polymastia corticata* | **Gear:**  ROV  **Photo by:**  *Holland I* 2018  **Identified by:**  J. Xavier  **Dive: 5** | (Ridley & Dendy, 1886) | **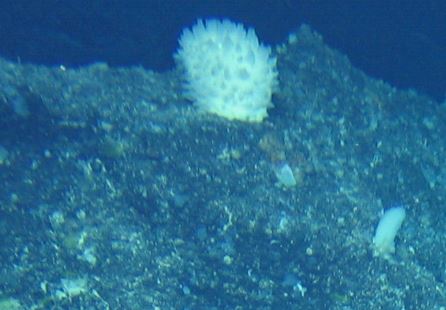** |
| **TAXONOMY** | **NAME** | **HABITAT** | **NOTES** | **IMAGE** |
| **Phylum:**  Porifera  **Class:** Hexactinellida  **Order:**  **Family:**  **Genus:**  **Species:** | **Morphospecies code:** HEXACT1 | **Gear:**  ROV  **Photo by:**  *Holland I* 2018  **Identified by:**  J. Xavier  **Dive: 6** |  | **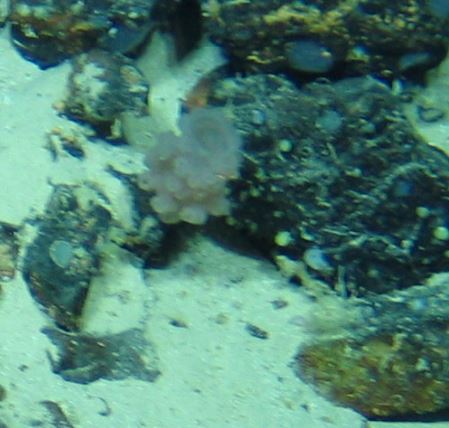** |
| **Phylum:**  Porifera  **Class:** Hexactinellida  **Order:**  **Family:**  **Genus:**  **Species:** | **Morphospecies code:** HEXACT2 | **Gear:**  ROV  **Photo by:**  *Holland I* 2018  **Identified by:**  J. Xavier  **Dive: 5** |  | **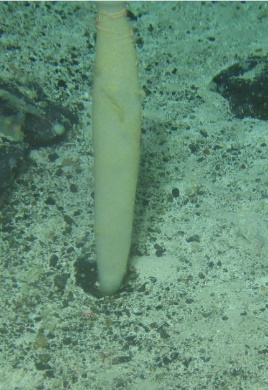** |
| **Phylum:**  Porifera  **Class:** Hexactinellida  **Order:**  **Family:**  **Genus:** *Hertwigia*  **Species:** H. *falcifera* | **Morphospecies code:** HEXACT3  *Hertwigia falcifera* | **Gear:**  ROV  **Photo by:**  *Holland I* 2018  **Identified by:**  J. Xavier  **Dive: 5** | yellow color morph (more common) (J. Xavier)  (Schmidt, 1880) | **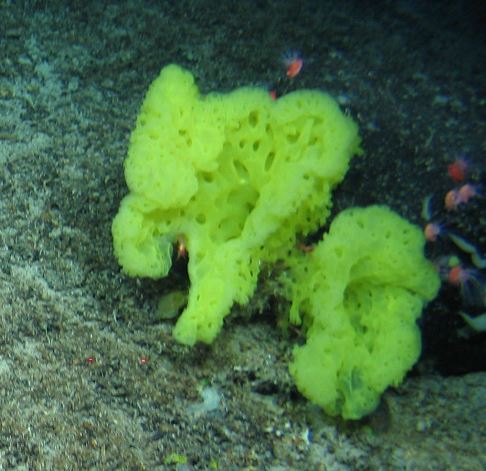** |
| **Phylum:**  Porifera  **Class:** Hexactinellida  **Order:**  **Family:**  **Genus:** *Hertwigia*  **Species:** H. *falcifera* | **Morphospecies code:** HEXACT4  cf. *Hertwigia falcifera* | **Gear:**  ROV  **Photo by:**  *Holland I* 2018  **Identified by:**  J. Xavier  **Dive: 8** | Probably the same as HEXACT3, just different growth form  (J. Xavier) | **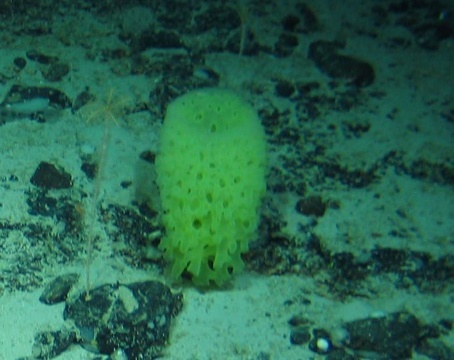** |
| **Phylum:**  Porifera  **Class:** Hexactinellida  **Order:**  **Family:**  **Genus:**  **Species:** | **Morphospecies code:** HEXACT5 | **Gear:**  ROV  **Photo by:**  *Holland I* 2018  **Identified by:**  J. Xavier  **Dive: 6** |  | **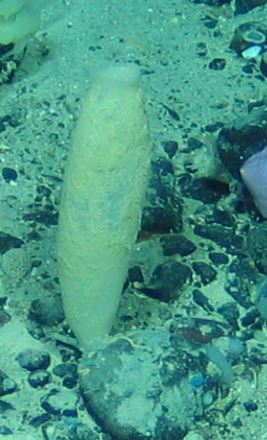** |
| **TAXONOMY** | **NAME** | **HABITAT** | **NOTES** | **IMAGE** |
| **Phylum:**  Porifera  **Class:** Hexactinellida  **Order:**  **Family:**  **Genus:** *Amphidiscella*  **Species:** | **Morphospecies code:** HEXACT6  *Amphidiscella* sp. | **Gear:**  ROV  **Photo by:**  *Holland I* 2018  **Identified by:**  J. Xavier  **Dive: 6** |  | **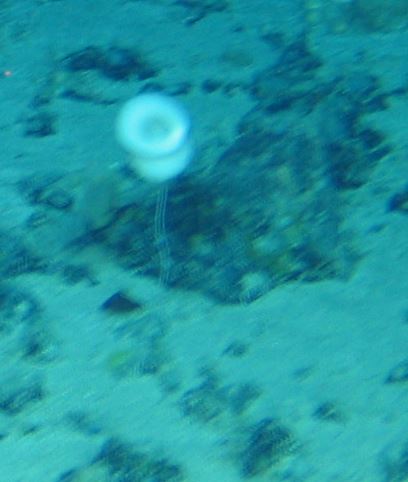** |
| **Phylum:**  Porifera  **Class:** Hexactinellida  **Order:**  **Family:**  **Genus:** *Asconema*  **Species:** | **Morphospecies code:** HEXACT7  *Asconema* sp. | **Gear:**  ROV  **Photo by:**  *Holland I* 2018  **Identified by:**  J. Xavier  **Dive: 7** |  | **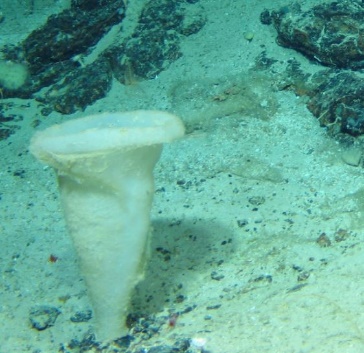** |
| **Phylum:**  Porifera  **Class:** Hexactinellida  **Order:**  **Family:**  **Genus:**  **Species:** | **Morphospecies code:** HEXACT8 | **Gear:**  ROV  **Photo by:**  *Holland I* 2018  **Identified by:**  J. Xavier  **Dive: 6** |  | **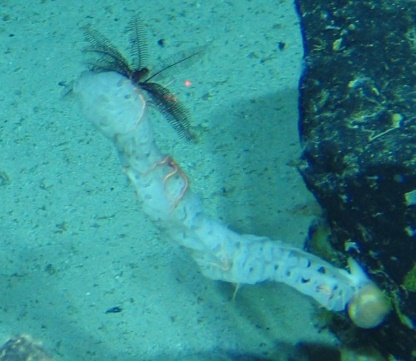** |
| **Phylum:**  Porifera  **Class:** Hexactinellida  **Order:**  **Family:**  **Genus:** *Hertwigia*  **Species:** *H.* *falcifera* | **Morphospecies code:** HEXACT9  *Hertwigia falcifera* | **Gear:**  ROV  **Photo by:**  *Holland I* 2018  **Identified by:**  J. Xavier  **Dive: 5** | (Schmidt, 1880)  white color morph | **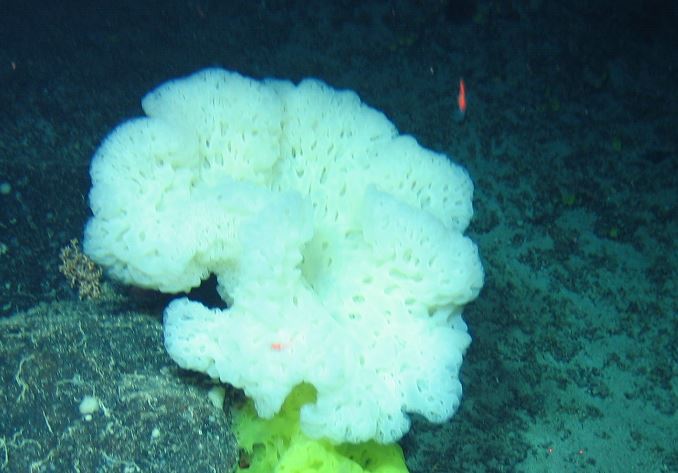** |
| **Phylum:**  Porifera  **Class:** Hexactinellida  **Order:**  **Family:**  **Genus:**  **Species:** | **Morphospecies code:** HEXACT10 | **Gear:**  ROV  **Photo by:**  *Holland I* 2018  **Identified by:**  J. Xavier  **Dive: 7** |  | **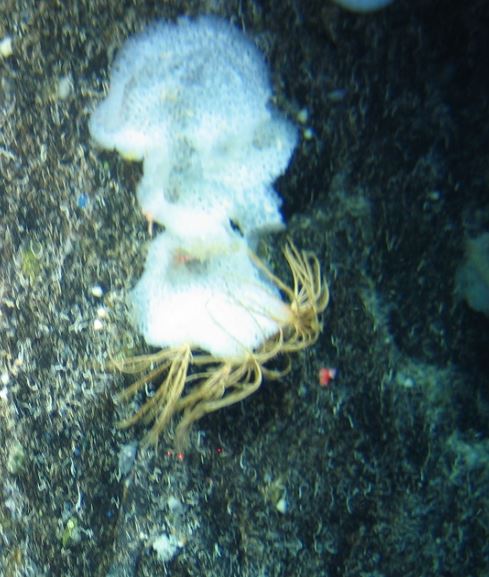** |
| **TAXONOMY** | **NAME** | **HABITAT** | **NOTES** | **IMAGE** |
| **Phylum:**  Porifera  **Class:** Hexactinellida  **Order:**  **Family:**  **Genus:**  **Species:** | **Morphospecies code:** HEXACT11 | **Gear:**  ROV  **Photo by:**  *Holland I* 2018  **Identified by:**  J. Xavier  **Dive: 6** |  | **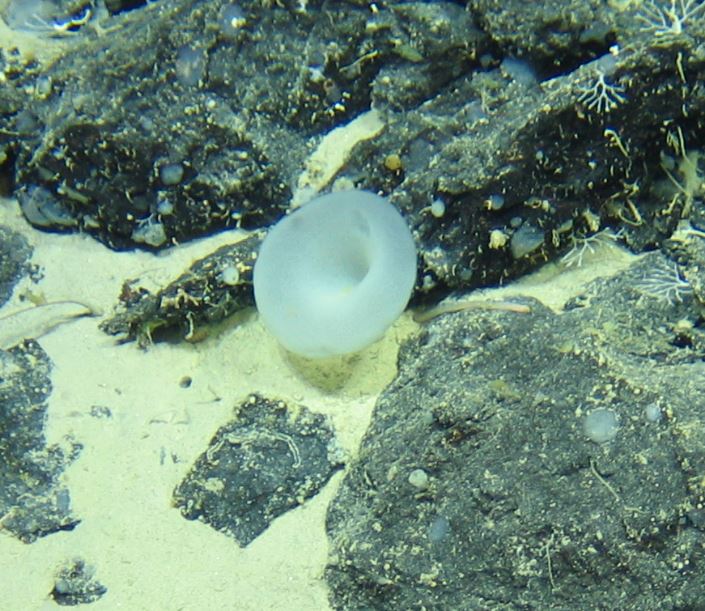** |
| **Phylum:**  Porifera  **Class:** Hexactinellida  **Order:**  **Family:**  **Genus:**  **Species:** | **Morphospecies code:** HEXACT13 | **Gear:**  ROV  **Photo by:**  *Holland I* 2018  **Identified by:**  **Dive: 5** |  | **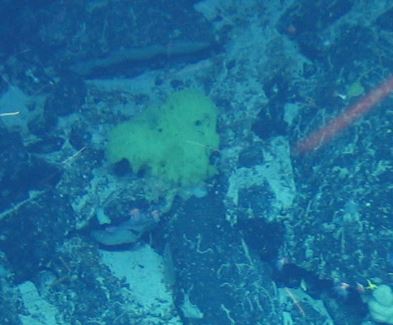** |
| **Phylum:**  Porifera  **Class:** Hexactinellida  **Order:**  **Family:**  **Genus:**  **Species:** | **Morphospecies code:** HEXACT14 | **Gear:**  ROV  **Photo by:**  *Holland I* 2018  **Identified by:**  **Dive: 5** | Not certain it is a Hexactinellida (J. Xavier) | **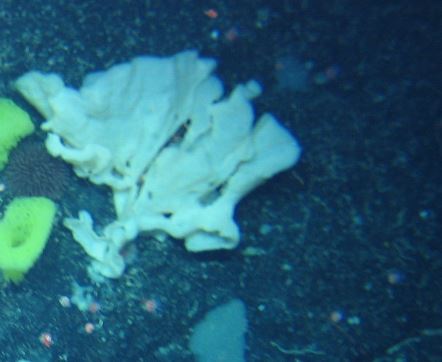** |
| **Phylum:**  Porifera  **Class:** Hexactinellida  **Order:**  **Family:**  **Genus:**  **Species:** | **Morphospecies code:** HEXACT15 | **Gear:**  ROV  **Photo by:**  *Holland I* 2018  **Identified by:**  **Dive: 5** | Not certain it is a Hexactinellida or Demospongiae  (J. Xavier) | **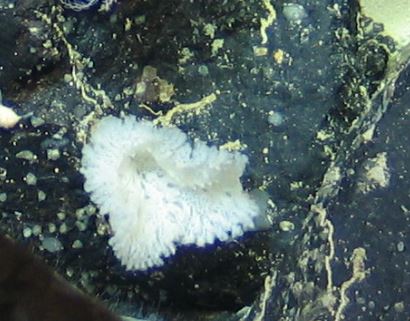** |
| **Phylum:**  Porifera  **Class:** Hexactinellida  **Order:**  **Family:**  **Genus:** *Farrea*  **Species:** | **Morphospecies code:** HEXACT16  *Farrea* sp. | **Gear:**  ROV  **Photo by:**  *Holland I* 2018  **Identified by:**  **Dive: 6** |  | **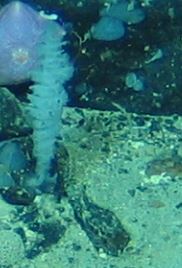** |
| **TAXONOMY** | **NAME** | **HABITAT** | **NOTES** | **IMAGE** |
| **Phylum:**  Porifera  **Class:** Hexactinellida  **Order:**  **Family:**  **Genus:**  **Species:** | **Morphospecies code:** HEXACT17 | **Gear:**  ROV  **Photo by:**  *Holland I* 2018  **Identified by:**  J. Xavier  **Dive: 7** | Possibly *Sympagella* or *Amphidiscella* (J. Xavier) | **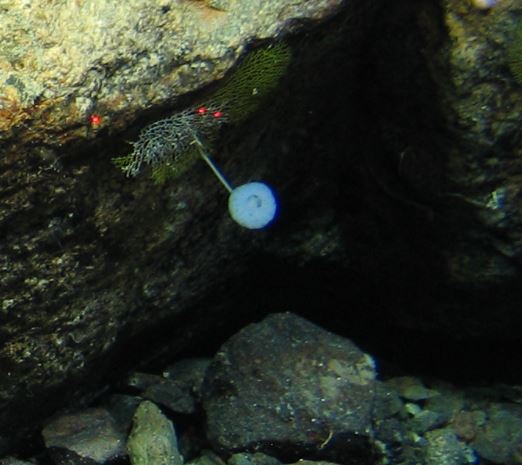** |
| **Phylum:**  Porifera  **Class:** Hexactinellida  **Order:**  **Family:**  **Genus:**  **Species:** | **Morphospecies code:** HEXACT18 | **Gear:**  ROV  **Photo by:**  *Holland I* 2018  **Identified by:**  J. Xavier  **Dive: 8** | Possibly *Hertwigia* sp. (J. Xavier) | **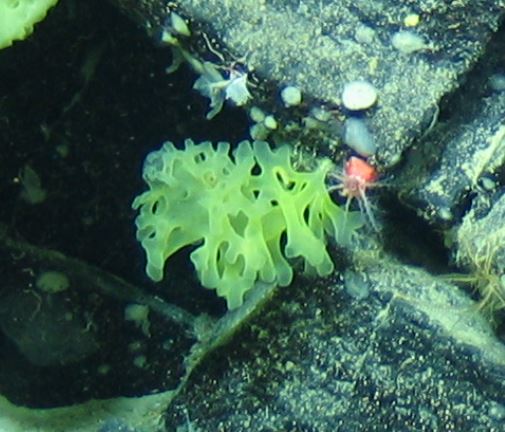** |
| **Phylum:**  Porifera  **Class:** Hexactinellida  **Order:**  **Family:**  **Genus:**  **Species:** | **Morphospecies code:** HEXACT20 | **Gear:**  ROV  **Photo by:**  *Holland I* 2018  **Identified by:**  **Dive: 5** |  | **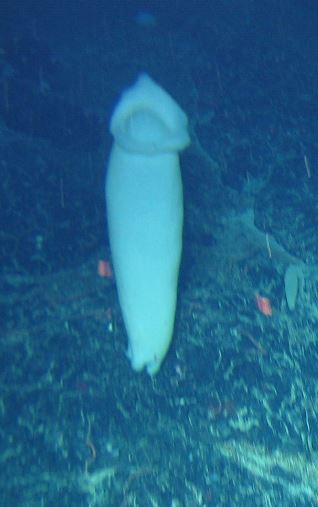** |
| **Phylum:**  Porifera  **Class:** Hexactinellida  **Order:**  **Family:**  **Genus:**  **Species:** | **Morphospecies code:** HEXACT21 | **Gear:**  ROV  **Photo by:**  *Holland I* 2018  **Identified by:**  **Dive: 7** |  | **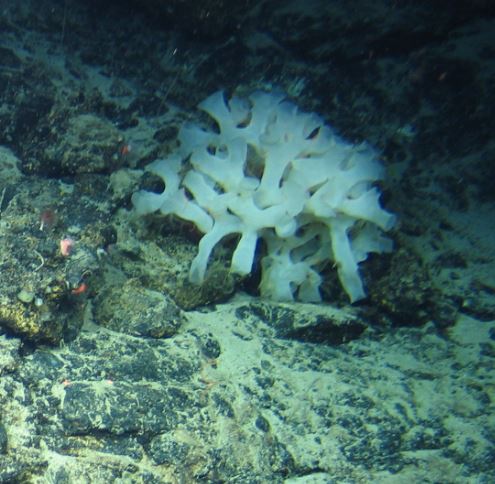** |
| **Phylum:**  Porifera  **Class:** Hexactinellida  **Order:**  **Family:**  **Genus:**  **Species:** | **Morphospecies code:** HEXACT22 | **Gear:**  ROV  **Photo by:**  *Holland I* 2018  **Identified by:**  **Dive: 8** |  | **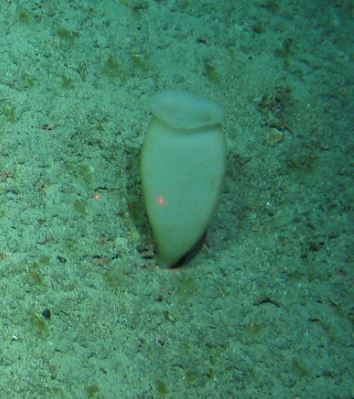** |
| **TAXONOMY** | **NAME** | **HABITAT** | **NOTES** | **IMAGE** |
| **Phylum:**  Porifera  **Class:** Hexactinellida  **Order:**  **Family:**  **Genus:**  **Species:** | **Morphospecies code:** HEXACT24 | **Gear:**  ROV  **Photo by:**  *Holland I* 2018  **Identified by:**  **Dive: 8** |  | **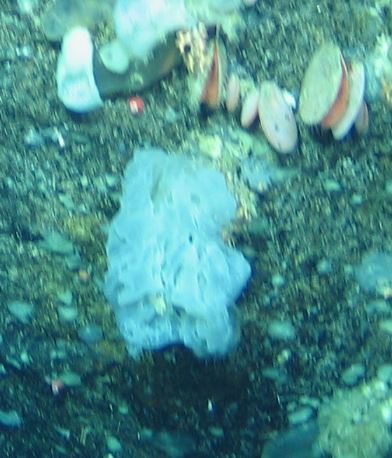** |
| **Phylum:**  Porifera  **Class:** Hexactinellida  **Order:**  **Family:**  **Genus:**  **Species:** | **Morphospecies code:** HEXACT25 | **Gear:**  ROV  **Photo by:**  *Holland I* 2018  **Identified by:**  **Dive: 7** |  | **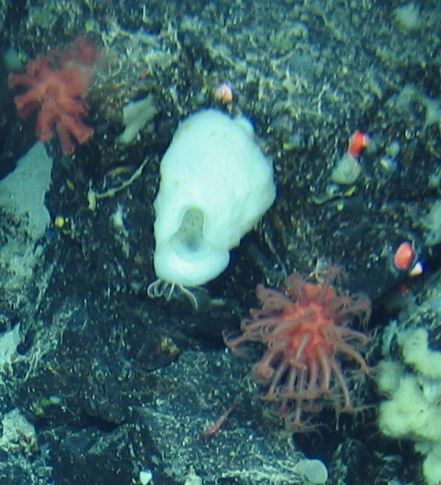** |
| **Phylum:**  Porifera  **Class:** Hexactinellida  **Order:**  **Family:**  **Genus:**  **Species:** | **Morphospecies code:** HEXACT26 | **Gear:**  ROV  **Photo by:**  *Holland I* 2018  **Identified by:**  **Dive: 9** |  | **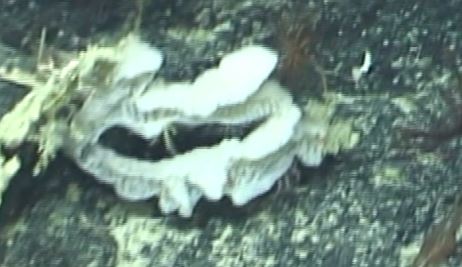** |
| **Phylum:**  Porifera  **Class:** Hexactinellida  **Order:**  **Family:**  **Genus:**  **Species:** | **Morphospecies code:** HEXACT27 | **Gear:**  ROV  **Photo by:**  *Holland I* 2018  **Identified by:**  **Dive: 6** |  | **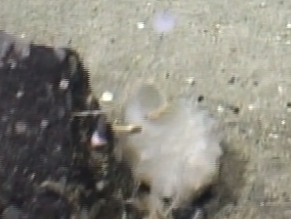** |
| **Phylum:**  Porifera  **Class:** Hexactinellida  **Order:** Lyssacinosida  **Family:** Euplectellida  **Genus:**  **Species:** | **Morphospecies code:** EUPL1 | **Gear:**  ROV  **Photo by:**  *Holland I* 2018  **Identified by:**  **Dive: 5** | This could be *Euplectella, Dictyaulus, Regadrella*  (J. Xavier) | **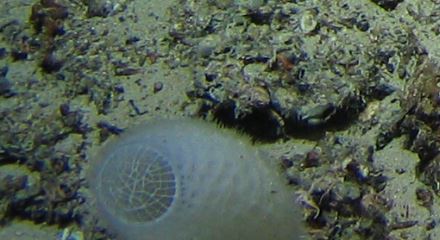** |
| **TAXONOMY** | **NAME** | **HABITAT** | **NOTES** | **IMAGE** |
| **Phylum:** Bryozoa  **Class:**  **Order:**  **Family:**  **Genus:**  **Species:** | **Morphospecies code:** BRYOZ1 | **Gear:**  ROV  **Photo by:**  *Holland I* 2018  **Identified by:**  **Dive: 6** |  | **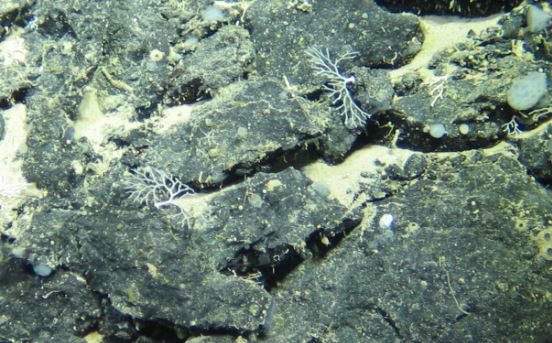** |
| **Phylum:** Bryozoa  **Class:**  **Order:**  **Family:**  **Genus:**  **Species:** | **Morphospecies code:** BRYOZ2 | **Gear:**  ROV  **Photo by:**  *Holland I* 2018  **Identified by:** Poppy Keogh  **Dive: 6** |  | **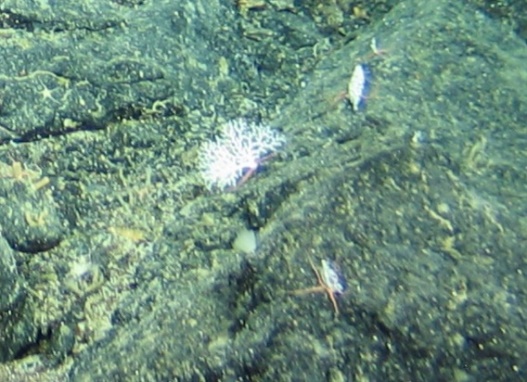** |
| **Phylum:** Bryozoa  **Class:** Gymnolaemata  **Order:**  **Family:**  **Genus:** *Canda*  **Species:** | **Morphospecies code:** CANDA  *Canda* sp. | **Gear:**  ROV  **Photo by:**  *Holland I* 2018  **Identified by:** Megan McCuller  **Dive: 6** |  | **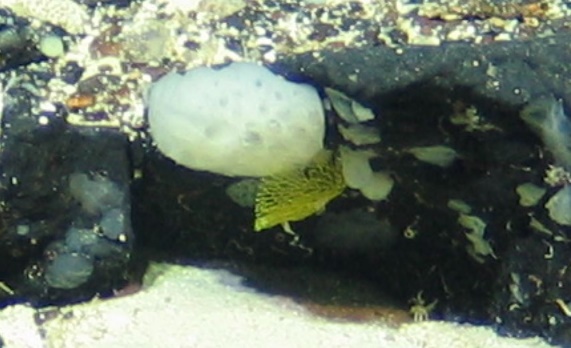** |
| **Phylum:** Cnidaria  **Class:** Anthozoa  **Subclass:**  Hexacorillia  **Order:** Antipatharia  **Family:**  **Genus:**  **Species:** | **Morphospecies code:** ANTIPATHARIA1 | **Gear:**  ROV  **Photo by:**  *Holland I* 2018  **Identified by:** Poppy Keogh  **Dive: 6** |  | **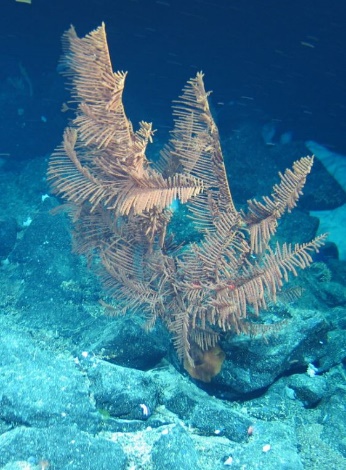** |
| **Phylum:** Cnidaria  **Class:** Anthozoa  **Subclass:**  Hexacorillia  **Order:** Antipatharia  **Family:**  **Genus:**  **Species:** | **Morphospecies code:** ANTIPATHARIA2 | **Gear:**  ROV  **Photo by:**  *Holland I* 2018  **Identified by:** Poppy Keogh  **Dive: 9** |  | **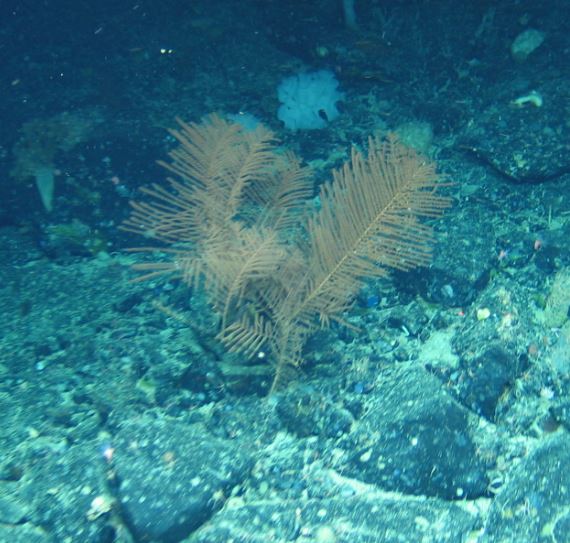** |

| **TAXONOMY** | **NAME** | **HABITAT** | **NOTES** | **IMAGE** |
| --- | --- | --- | --- | --- |
| **Phylum:** Cnidaria  **Class:** Anthozoa  **Subclass:**  Hexacorillia  **Order:** Antipatharia  **Family:**  **Genus:**  **Species:** | **Morphospecies code:** ANTIPATHARIA3 | **Gear:**  ROV  **Photo by:**  *Holland I* 2018  **Identified by:** Poppy Keogh  **Dive: 6** |  | **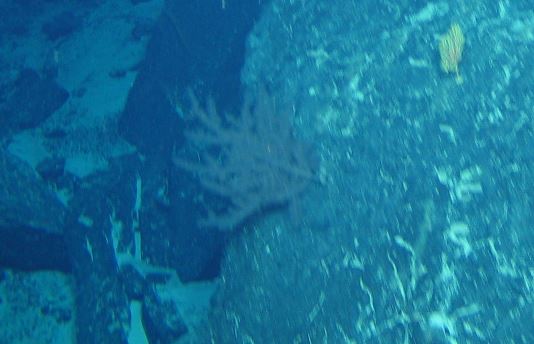** |
| **Phylum:** Cnidaria  **Class:** Anthozoa  **Subclass:**  Hexacorillia  **Order:** Antipatharia  **Family:**  **Genus:** *Stauropathes*  **Species:** *S.* *arctica* | **Morphospecies code:** ANTIPATHARIA4  cf. *Stauropathes arctica* | **Gear:**  ROV  **Photo by:**  *Holland I* 2018  **Identified by:** Tina Molodtsova  **Dive: 5** |  | **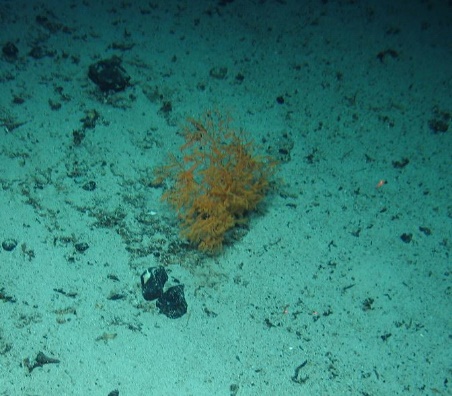** |
| **Phylum:** Cnidaria  **Class:** Anthozoa  **Subclass:**  Hexacorillia  **Order:** Antipatharia  **Family:**  **Genus:**  **Species:** | **Morphospecies code:** ANTIPATHARIA6 | **Gear:**  ROV  **Photo by:**  *Holland I* 2018  **Identified by:** Poppy Keogh  **Dive: 9** |  | **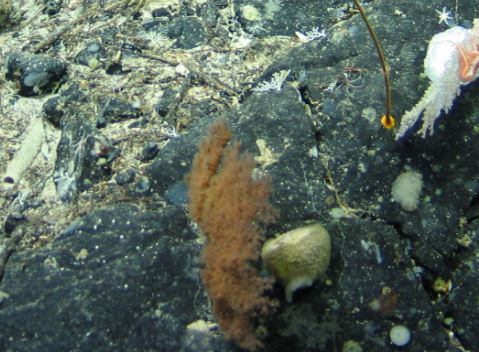** |
| **Phylum:** Cnidaria  **Class:** Anthozoa  **Subclass:**  Hexacorillia  **Order:** Antipatharia  **Family:**  **Genus:** *Stauropathes*  **Species:** *S.* *arctica* | **Morphospecies code:** ANTIPATHARIA7  cf. *Stauropathes arctica* | **Gear:**  ROV  **Photo by:**  *Holland I* 2018  **Identified by:** Tina Molodtsova  **Dive: 6** |  | **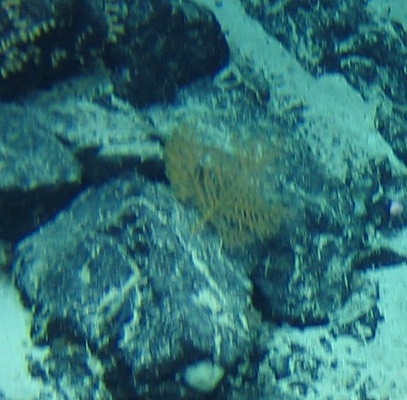** |
| **TAXONOMY** | **NAME** | **HABITAT** | **NOTES** | **IMAGE** |
| **Phylum:** Cnidaria  **Class:** Anthozoa  **Subclass:**  Hexacorillia  **Order:** Antipatharia  **Family:** Schizopathidae  **Genus:** *Parantipathes*  **Species:** | **Morphospecies code:** ANTIPATHARIA8  *Parantipathes* sp. | **Gear:**  ROV  **Photo by:**  *Holland I* 2018  **Identified by:** Tina Molodtsova  **Dive: 7** |  | **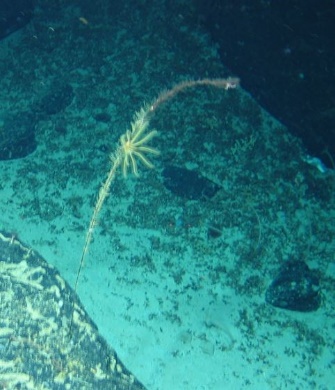** |
| **Phylum:** Cnidaria  **Class:** Anthozoa  **Subclass:**  Hexacorillia  **Order:** Antipatharia  **Family:**  **Genus:**  **Species:** | **Morphospecies code:** ANTIPATHARIA9 | **Gear:**  ROV  **Photo by:**  *Holland I* 2018  **Identified by:** Poppy Keogh  **Dive: 9** |  | **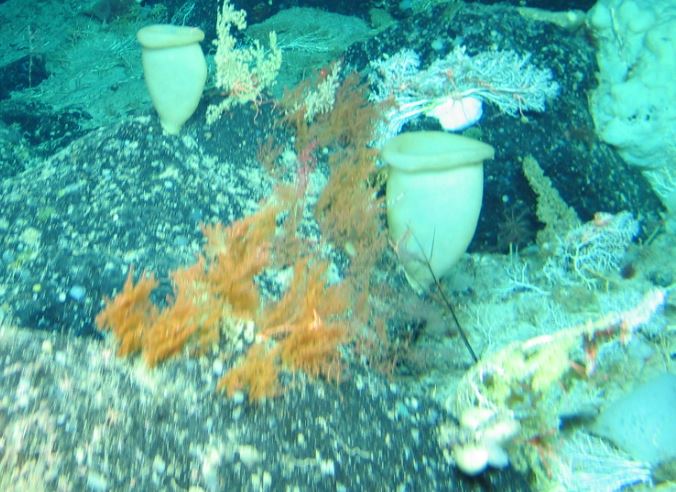** |
| **Phylum:** Cnidaria  **Class:** Anthozoa  **Subclass:**  Hexacorillia  **Order:** Antipatharia  **Family:**  **Genus:**  **Species:** | **Morphospecies code:** ANTIPATHARIA10 | **Gear:**  ROV  **Photo by:**  *Holland I* 2018  **Identified by:** Poppy Keogh  **Dive: 9** |  | **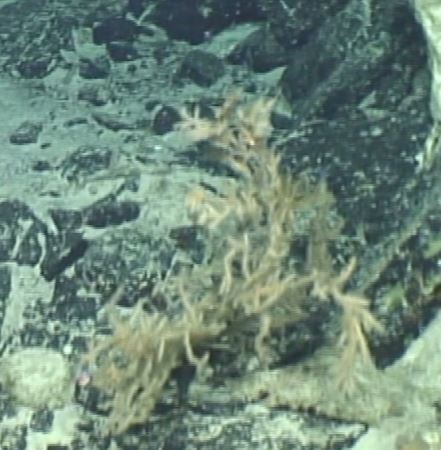** |
| **Phylum:** Cnidaria  **Class:** Anthozoa  **Subclass:**  Hexacorillia  **Order:** Antipatharia  **Family:**  **Genus:**  **Species:** | **Morphospecies code:** ANTIPATHARIA11 | **Gear:**  ROV  **Photo by:**  *Holland I* 2018  **Identified by:** Poppy Keogh  **Dive: 9** |  | **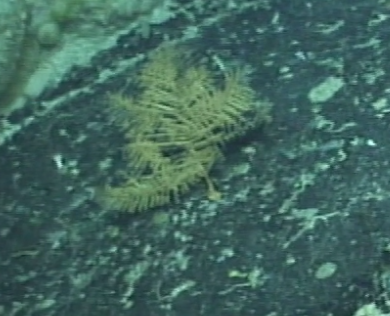** |
| **TAXONOMY** | **NAME** | **HABITAT** | **NOTES** | **IMAGE** |
| **Phylum:** Cnidaria  **Class:** Anthozoa  **Subclass:**  Hexacorillia  **Order:** Antipatharia  **Family:**  **Genus:**  **Species:** | **Morphospecies code:** ANTIPATHARIA12 | **Gear:**  ROV  **Photo by:**  *Holland I* 2018  **Identified by:** Poppy Keogh  **Dive: 6** |  | **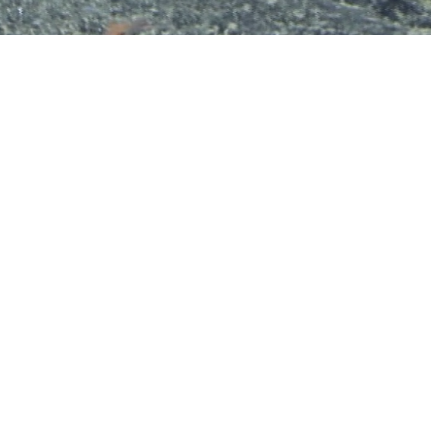** |
| **Phylum:** Cnidaria  **Class:** Anthozoa  **Subclass:**  Hexacorillia  **Order:** Antipatharia  **Family:**  **Genus:**  **Species:** | **Morphospecies code:** ANTIPATHARIA13 | **Gear:**  ROV  **Photo by:**  *Holland I* 2018  **Identified by:** Poppy Keogh  **Dive: 6** |  | **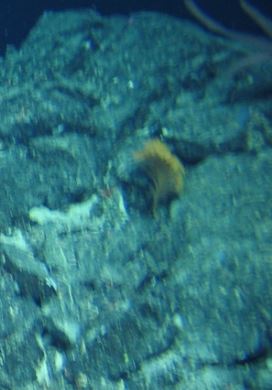** |
| **Phylum:** Cnidaria  **Class:** Anthozoa  **Subclass:**  Hexacorillia  **Order:** Antipatharia  **Family:** Schizopathidae  **Genus:** *Bathypathes*  **Species:** | **Morphospecies code:** BATHY1  *Bathypathes* n.sp. | **Gear:**  ROV  **Photo by:**  *Holland I* 2018  **Identified by:** Tina Molodtsova  **Dive: 9** | *Bathypathes* n.sp. under description (T. Molodtsova) | **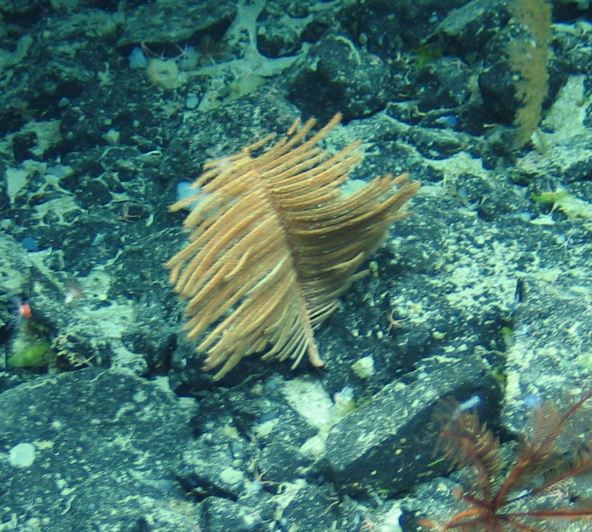** |
| **Phylum:** Cnidaria  **Class:** Anthozoa  **Subclass:**  Hexacorillia  **Order:** Antipatharia  **Family:** Schizopathidae  **Genus:** *Bathypathes*  **Species:** | **Morphospecies code:** BATHY2  *Bathypathes* sp. | **Gear:**  ROV  **Photo by:**  *Holland I* 2018  **Identified by:** Poppy Keogh  **Dive: 7** |  | **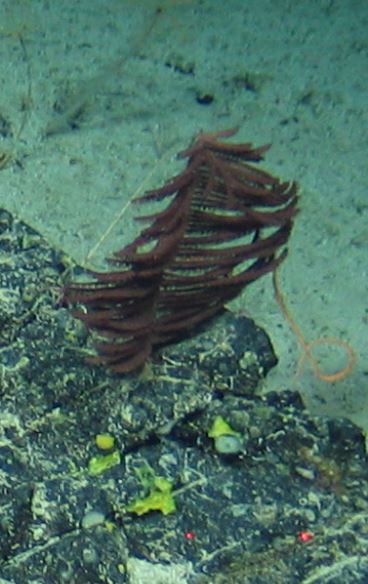** |
| **TAXONOMY** | **NAME** | **HABITAT** | **NOTES** | **IMAGE** |
| **Phylum:** Cnidaria  **Class:** Anthozoa  **Subclass:**  Hexacorillia  **Order:** Antipatharia  **Family:** Schizopathidae  **Genus:** *Bathypathes*  **Species:** | **Morphospecies code:** BATHY3  *Bathypathes* sp. | **Gear:**  ROV  **Photo by:**  *Holland I* 2018  **Identified by:** Poppy Keogh  **Dive: 8** |  | **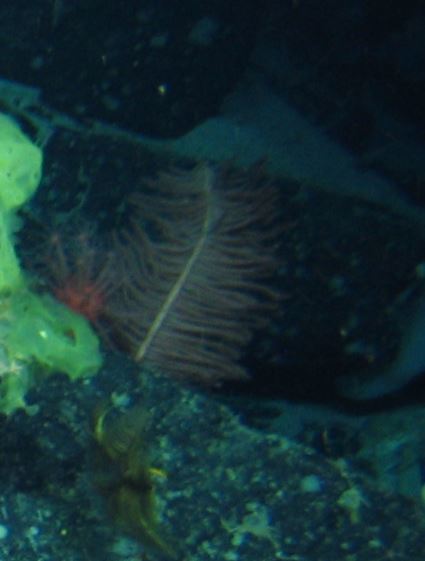** |
| **Phylum:** Cnidaria  **Class:** Anthozoa  **Subclass:**  Hexacorillia  **Order:** Antipatharia  **Family:** Schizopathidae  **Genus:** *Bathypathes*  **Species:** | **Morphospecies code:** BATHY4  *Bathypathes* sp. | **Gear:**  ROV  **Photo by:**  *Holland I* 2018  **Identified by:** Poppy Keogh  **Dive: 6** |  | **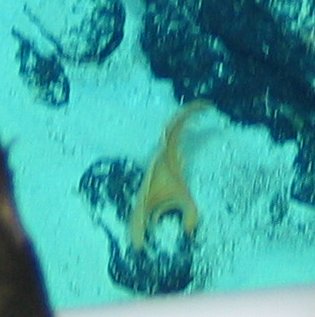** |
| **Phylum:** Cnidaria  **Class:** Anthozoa  **Subclass:**  Hexacorillia  **Order:** Antipatharia  **Family:** Schizopathidae  **Genus:** *Bathypathes*  **Species:** | **Morphospecies code:** BATHY5  *Bathypathes* sp. | **Gear:**  ROV  **Photo by:**  *Holland I* 2018  **Identified by:** Poppy Keogh  **Dive: 5** |  | **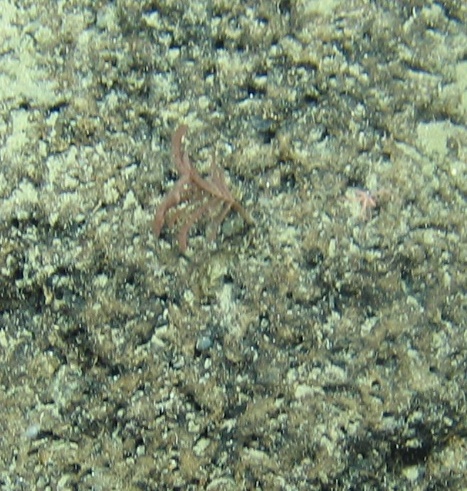** |
| **Phylum:** Cnidaria  **Class:** Anthozoa  **Subclass:**  Hexacorillia  **Order:** Antipatharia  **Family:** Schizopathidae  **Genus:** *Bathypathes*  **Species:** | **Morphospecies code:** BATHY6  *Bathypathes* sp. | **Gear:**  ROV  **Photo by:**  *Holland I* 2018  **Identified by:** Poppy Keogh  **Dive: 9** |  | **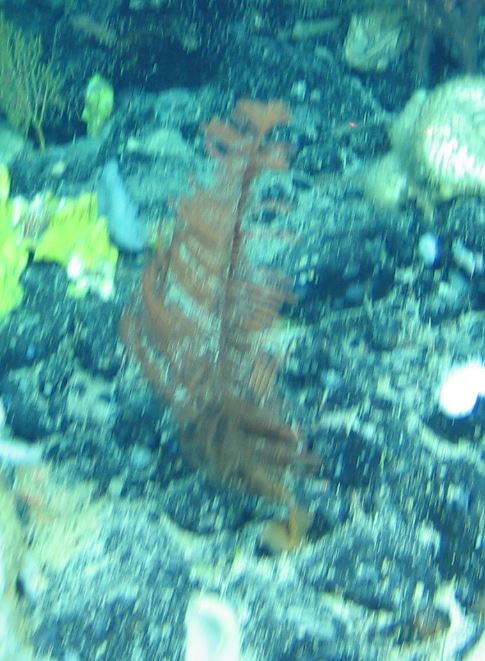** |
| **TAXONOMY** | **NAME** | **HABITAT** | **NOTES** | **IMAGE** |
| **Phylum:** Cnidaria  **Class:** Anthozoa  **Subclass:**  Hexacorillia  **Order:** Antipatharia  **Family:** Leiopathidae  **Genus:** *Leiopathes*  **Species:** | **Morphospecies code:** LEIOPAT  *Leiopathes* sp. | **Gear:**  ROV  **Photo by:**  *Holland I* 2018  **Identified by:** Tina Molodtsova  **Dive: 5** |  | **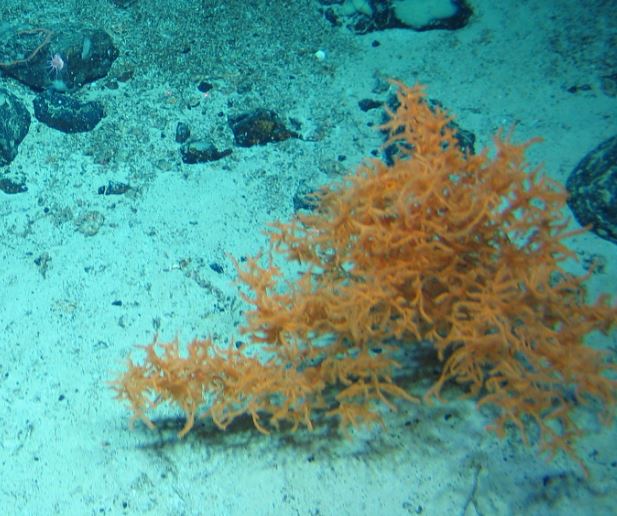** |
| **Phylum:** Cnidaria  **Class:** Anthozoa  **Subclass:**  Hexacorillia  **Order:** Antipatharia  **Family:** Schizopathidae  **Genus:** *Parantipathes*  **Species:** | **Morphospecies code:** PARAN1  *Parantipathes* sp. | **Gear:**  ROV  **Photo by:**  *Holland I* 2018  **Identified by:** Poppy Keogh  **Dive: 6** |  | **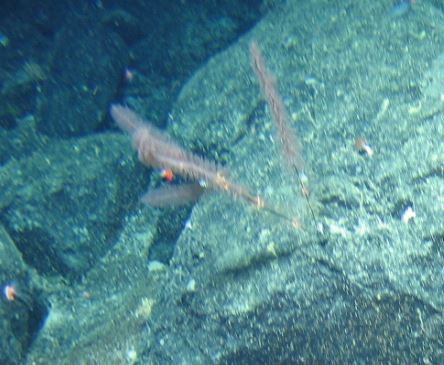** |
| **Phylum:** Cnidaria  **Class:** Anthozoa  **Subclass:**  Hexacorillia  **Order:** Antipatharia  **Family:** Schizopathidae  **Genus:** *Parantipathes*  **Species:** | **Morphospecies code:** PARAN2  *Parantipathes* sp. | **Gear:**  ROV  **Photo by:**  *Holland I* 2018  **Identified by:** Poppy Keogh  **Dive: 5** |  | **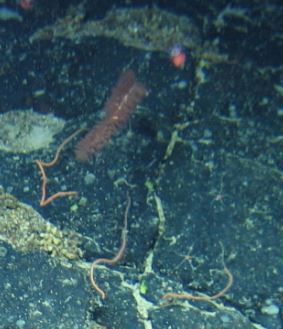** |
| **Phylum:** Cnidaria  **Class:** Anthozoa  **Subclass:**  Hexacorillia  **Order:** Antipatharia  **Family:** Schizopathidae  **Genus:** *Parantipathes*  **Species:** P. *hirondelle* | **Morphospecies code:** PARAN3  cf. *Parantipathes hirondelle* | **Gear:**  ROV  **Photo by:**  *Holland I* 2018  **Identified by:** Tina Molodtsova  **Dive: 8** |  |  |
| **TAXONOMY** | **NAME** | **HABITAT** | **NOTES** | **IMAGE** |
| **Phylum:** Cnidaria  **Class:** Anthozoa  **Subclass:**  Hexacorillia  **Order:** Antipatharia  **Family:** Schizopathidae  **Genus:** *Parantipathes*  **Species:** | **Morphospecies code:** PARAN4  *Parantipathes* sp. | **Gear:**  ROV  **Photo by:**  *Holland I* 2018  **Identified by:** Poppy Keogh  **Dive: 7** |  |  |
| **Phylum:** Cnidaria  **Class:** Anthozoa  **Subclass:**  Hexacorillia  **Order:** Antipatharia  **Family:** Schizopathidae  **Genus:** *Stauropathes*  **Species:** | **Morphospecies code:** STAURO  *Stauropathes* sp. | **Gear:**  ROV  **Photo by:**  *Holland I* 2018  **Identified by:** Poppy Keogh  **Dive: 8** |  |  |
| **Phylum:** Cnidaria  **Class:** Anthozoa  **Subclass:**  Hexacorillia  **Order:** Antipatharia  **Family:** Antipathidae  **Genus:** *Stichopathes*  **Species:** | **Morphospecies code:** STICHO1  *Stichopathes* sp. | **Gear:**  ROV  **Photo by:**  *Holland I* 2018  **Identified by:** Poppy Keogh  **Dive: 6** |  |  |
| **Phylum:** Cnidaria  **Class:** Anthozoa  **Subclass:**  Hexacorillia  **Order:** Antipatharia  **Family:** Schizopathidae  **Genus:** *Parantipathes*  **Species:** | **Morphospecies code:** TRISSO1  *Parantipathes* sp. | **Gear:**  ROV  **Photo by:**  *Holland I* 2018  **Identified by:** Tina Molodtsova  **Dive: 9** | Misidentified as a member of Trissopathes genus |  |
| **TAXONOMY** | **NAME** | **HABITAT** | **NOTES** | **IMAGE** |
| **Phylum:** Cnidaria  **Class:** Anthozoa  **Subclass:** Hexacorillia  **Order:** Actiniaria  **Family:**  **Genus:**  **Species:** | **Morphospecies code:** ANEMONE1 | **Gear:**  ROV  **Photo by:**  *Holland I* 2018  **Identified by:** Poppy Keogh  **Dive: 5** |  |  |
| **Phylum:** Cnidaria  **Class:** Anthozoa  **Subclass:** Hexacorillia  **Order:** Actiniaria  **Family:** Actinernidae  **Genus:** *Actinernus*  **Species:** | **Morphospecies code:** ANEMONE2  *Actinernus* sp. | **Gear:**  ROV  **Photo by:**  *Holland I* 2018  **Identified by:** Tina Molodtsova  **Dive: 5** |  |  |
| **Phylum:** Cnidaria  **Class:** Anthozoa  **Subclass:** Hexacorillia  **Order:** Actiniaria  **Family:** Liponematidae  **Genus:** *Liponema*  **Species:** | **Morphospecies code:** ANEMONE3  cf *Liponema* | **Gear:**  ROV  **Photo by:**  *Holland I* 2018  **Identified by:** Tina Molodtsova  **Dive: 5** |  |  |
| **Phylum:** Cnidaria  **Class:** Anthozoa  **Subclass:** Hexacorillia  **Order:** Actiniaria  **Family:**  **Genus:**  **Species:** | **Morphospecies code:** ANEMONE4 | **Gear:**  ROV  **Photo by:**  *Holland I* 2018  **Identified by:** Poppy Keogh  **Dive: 7** |  |  |
| **TAXONOMY** | **NAME** | **HABITAT** | **NOTES** | **IMAGE** |
| **Phylum:** Cnidaria  **Class:** Anthozoa  **Subclass:** Hexacorillia  **Order:** Actiniaria  **Family:**  **Genus:**  **Species:** | **Morphospecies code:** ANEMONE5 | **Gear:**  ROV  **Photo by:**  *Holland I* 2018  **Identified by:** Poppy Keogh  **Dive: 8** | Possibly Actinoscyphia sp. (P. Keogh) |  |
| **Phylum:** Cnidaria  **Class:** Anthozoa  **Subclass:** Hexacorillia  **Order:** Actiniaria  **Family:**  **Genus:**  **Species:** | **Morphospecies code:** ANEMONE6 | **Gear:**  ROV  **Photo by:**  *Holland I* 2018  **Identified by:** Poppy Keogh  **Dive: 7** | Actinostolidae sp.? (P Keogh) |  |
| **Phylum:** Cnidaria  **Class:** Anthozoa  **Subclass:** Hexacorillia  **Order:** Actiniaria  **Family:**  **Genus:**  **Species:** | **Morphospecies code:** ANEMONE7 | **Gear:**  ROV  **Photo by:**  *Holland I* 2018  **Identified by:** Poppy Keogh  **Dive: 8** |  |  |
| **Phylum:** Cnidaria  **Class:** Anthozoa  **Subclass:** Hexacorillia  **Order:** Actiniaria  **Family:**  **Genus:**  **Species:** | **Morphospecies code:** ANEMONE8 | **Gear:**  ROV  **Photo by:**  *Holland I* 2018  **Identified by:** Poppy Keogh  **Dive: 8** | Possibly *Bolocera tuediae* (P. Keogh) |  |
| **TAXONOMY** | **NAME** | **HABITAT** | **NOTES** | **IMAGE** |
| **Phylum:** Cnidaria  **Class:** Anthozoa  **Subclass:** Hexacorillia  **Order:** Corallimorpharia  **Family:** Corallimorphidae  **Genus:** *Corallimorphus*  **Species:** | **Morphospecies code:** ANEMONE9  *Corallimorphus* sp. | **Gear:**  ROV  **Photo by:**  *Holland I* 2018  **Identified by:** Tina Molodtsova  **Dive: 7** |  |  |
| **Phylum:** Cnidaria  **Class:** Anthozoa  **Subclass:** Hexacorillia  **Order:** Actiniaria  **Family:**  **Genus:**  **Species:** | **Morphospecies code:** ANEMONE10 | **Gear:**  ROV  **Photo by:**  *Holland I* 2018  **Identified by:** Poppy Keogh  **Dive: 5** |  |  |
| **Phylum:** Cnidaria  **Class:** Anthozoa  **Subclass:** Hexacorillia  **Order:** Actiniaria  **Family:**  **Genus:**  **Species:** | **Morphospecies code:** ANEMONE12 | **Gear:**  ROV  **Photo by:**  *Holland I* 2018  **Identified by:** Poppy Keogh  **Dive: 8** |  |  |
| **Phylum:** Cnidaria  **Class:** Anthozoa  **Subclass:** Hexacorillia  **Order:** Actiniaria  **Family:**  **Genus:**  **Species:** | **Morphospecies code:** ANEMONE13 | **Gear:**  ROV  **Photo by:**  *Holland I* 2018  **Identified by:** Poppy Keogh  **Dive: 6** | Possibly *Phelliactis hertwigii* (P. Keogh) |  |
| **TAXONOMY** | **NAME** | **HABITAT** | **NOTES** | **IMAGE** |
| **Phylum:** Cnidaria  **Class:** Anthozoa  **Subclass:** Ceriantharia  **Order:**  **Family:**  **Genus:**  **Species:** | **Morphospecies code:** ANEMONE14  ? Ceriantharia? | **Gear:**  ROV  **Photo by:**  *Holland I* 2018  **Identified by:** Tina Molodtsova  **Dive: 7** |  |  |
| **Phylum:** Cnidaria  **Class:** Anthozoa  **Subclass:** Ceriantharia  **Order:**  **Family:**  **Genus:**  **Species:** | **Morphospecies code:** ANEMONE15  ? Ceriantharia? | **Gear:**  ROV  **Photo by:**  *Holland I* 2018  **Identified by:** Tina Molodtsova  **Dive: 9** |  |  |
| **Phylum:** Cnidaria  **Class:** Anthozoa  **Subclass:** Hexacorillia  **Order:** Actiniaria  **Family:**  **Genus:**  **Species:** | **Morphospecies code:** ANEMONE18 | **Gear:**  ROV  **Photo by:**  *Holland I* 2018  **Identified by:** Poppy Keogh  **Dive: 5** |  |  |
| **Phylum:** Cnidaria  **Class:** Anthozoa  **Subclass:** Hexacorillia  **Order:** Scleractinia  **Family:**  **Genus:**  **Species:** | **Morphospecies code:** ANEMONE19  ?Scleractinia | **Gear:**  ROV  **Photo by:**  *Holland I* 2018  **Identified by:** Tina Molodtsova  **Dive: 8** | Probably *Flabellum* sp. (T. Molodtsova) |  |
| **Phylum:** Cnidaria  **Class:** Anthozoa  **Subclass:** Hexacorillia  **Order:** Actiniaria  **Family:**  **Genus:**  **Species:** | **Morphospecies code:** ANEMONE20 | **Gear:**  ROV  **Photo by:**  *Holland I* 2018  **Identified by:** Poppy Keogh  **Dive: 6** |  |  |
| **TAXONOMY** | **NAME** | **HABITAT** | **NOTES** | **IMAGE** |
| **Phylum:** Cnidaria  **Class:** Anthozoa  **Subclass:** Hexacorillia  **Order:** Scleractinia  **Family:**  **Genus:**  **Species:** | **Morphospecies code:** ANEMONE21  ? Scleractinia? | **Gear:**  ROV  **Photo by:**  *Holland I* 2018  **Identified by:** Tina Molodtsova  **Dive: 5** | looks like something flat, as Fungiocysthus (T. Molodtsova) |  |
| **Phylum:** Cnidaria  **Class:** Anthozoa  **Subclass:** Hexacorillia  **Order:** Actiniaria  **Family:** Hormathiidae  **Genus:**  **Species:** | **Morphospecies code:** ANEMONE22  Hormathiidae | **Gear:**  ROV  **Photo by:**  *Holland I* 2018  **Identified by:** Tina Molodtsova  **Dive: 8** |  |  |
| **Phylum:** Cnidaria  **Class:** Anthozoa  **Subclass:** Hexacorillia  **Order:** Actiniaria  **Family:**  **Genus:**  **Species:** | **Morphospecies code:** ANEMONE23 | **Gear:**  ROV  **Photo by:**  *Holland I* 2018  **Identified by:** Poppy Keogh  **Dive: 6** |  |  |
| **Phylum:** Cnidaria  **Class:** Anthozoa  **Subclass:** Hexacorillia  **Order:** Actiniaria  **Family:**  **Genus:**  **Species:** | **Morphospecies code:** ANEMONE24 | **Gear:**  ROV  **Photo by:**  *Holland I* 2018  **Identified by:** Poppy Keogh  **Dive: 6** |  |  |
| **TAXONOMY** | **NAME** | **HABITAT** | **NOTES** | **IMAGE** |
| **Phylum:** Cnidaria  **Class:** Anthozoa  **Subclass:** Ceriantharia  **Order:**  **Family:**  **Genus:**  **Species:** | **Morphospecies code:** CERANTID | **Gear:**  ROV  **Photo by:**  *Holland I* 2018  **Identified by:** Tina Molodtsova  **Dive: 8** |  |  |
| **Phylum:** Cnidaria  **Class:** Anthozoa  **Subclass:**  Hexacorillia  **Order:** Scleractinia  **Family:**  **Genus:**  **Species:** | **Morphospecies code:** CUP1 | **Gear:**  ROV  **Photo by:**  *Holland I* 2018  **Identified by:** Poppy Keogh  **Dive: 5** |  |  |
| **Phylum:** Cnidaria  **Class:** Anthozoa  **Subclass:**  Hexacorillia  **Order:** Scleractinia  **Family:**  **Genus:**  **Species:** | **Morphospecies code:** CUP2 | **Gear:**  ROV  **Photo by:**  *Holland I* 2018  **Identified by:** Poppy Keogh  **Dive: 7** |  |  |
| **Phylum:** Cnidaria  **Class:** Anthozoa  **Subclass:**  Hexacorillia  **Order:** Scleractinia  **Family:**  **Genus:**  **Species:** | **Morphospecies code:** CUP3 | **Gear:**  ROV  **Photo by:**  *Holland I* 2018  **Identified by:** Poppy Keogh  **Dive: 8** |  |  |
| **Phylum:** Cnidaria  **Class:** Anthozoa  **Subclass:**  Hexacorillia  **Order:** Scleractinia  **Family:**  **Genus:**  **Species:** | **Morphospecies code:** CUP4 | **Gear:**  ROV  **Photo by:**  *Holland I* 2018  **Identified by:** Poppy Keogh  **Dive: 7** |  |  |
| **TAXONOMY** | **NAME** | **HABITAT** | **NOTES** | **IMAGE** |
| **Phylum:** Cnidaria  **Class:** Anthozoa  **Subclass:**  Hexacorillia  **Order:** Scleractinia  **Family:**  **Genus:**  **Species:** | **Morphospecies code:** CUP5 | **Gear:**  ROV  **Photo by:**  *Holland I* 2018  **Identified by:** Poppy Keogh  **Dive: 9** |  |  |
| **Phylum:** Cnidaria  **Class:** Anthozoa  **Subclass:**  Hexacorillia  **Order:** Scleractinia  **Family:**  **Genus:**  **Species:** | **Morphospecies code:** CUP6 | **Gear:**  ROV  **Photo by:**  *Holland I* 2018  **Identified by:** Poppy Keogh  **Dive: 8** |  |  |
| **Phylum:** Cnidaria  **Class:** Anthozoa  **Subclass:**  Hexacorillia  **Order:** Scleractinia  **Family:**  **Genus:**  **Species:** | **Morphospecies code:** CUP7 | **Gear:**  ROV  **Photo by:**  *Holland I* 2018  **Identified by:** Poppy Keogh  **Dive: 6** |  |  |
| **Phylum:** Cnidaria  **Class:** Anthozoa  **Subclass:**  Hexacorillia  **Order:** Scleractinia  **Family:** Flabellidae  **Genus:** *Flabellum*  **Species:** | **Morphospecies code:** CUP8  *Flabellum* sp. | **Gear:**  ROV  **Photo by:**  *Holland I* 2018  **Identified by:** Tina Molodtsova  **Dive: 8** |  |  |
| **TAXONOMY** | **NAME** | **HABITAT** | **NOTES** | **IMAGE** |
| **Phylum:** Cnidaria  **Class:** Anthozoa  **Subclass:**  Hexacorillia  **Order:** Scleractinia  **Family:** Caryophylliidae  **Genus:** *Solenosmilia*  **Species:** | **Morphospecies code:** SCLERA1  *Solenosmilia* sp. | **Gear:**  ROV  **Photo by:**  *Holland I* 2018  **Identified by:** Rebecca Ross  **Dive: 8** |  |  |
| **Phylum:** Cnidaria  **Class:** Anthozoa  **Subclass:** Octocorallia  **Order:** Alcyonacea  **Family:** Coralliidae  **Genus:** *Corallium*  **Species:** | **Morphospecies code:** CORALI  *Corallium* sp. | **Gear:**  ROV  **Photo by:**  *Holland I* 2018  **Identified by:**  Renata Arantes  **Dive: 6** |  |  |
| **Phylum:** Cnidaria  **Class:** Anthozoa  **Subclass:** Octocorallia  **Order:**  **Family:**  **Genus:**  **Species:** | **Morphospecies code:** OCTOCORAL1 | **Gear:**  ROV  **Photo by:**  *Holland I* 2018  **Identified by:** Poppy Keogh  **Dive: 8** |  |  |
| **Phylum:** Cnidaria  **Class:** Anthozoa  **Subclass:** Octocorallia  **Order:**  **Family:**  **Genus:**  **Species:** | **Morphospecies code:** OCTOCORAL2 | **Gear:**  ROV  **Photo by:**  *Holland I* 2018  **Identified by:**  Poppy Keogh  **Dive: 9** |  |  |
| **TAXONOMY** | **NAME** | **HABITAT** | **NOTES** | **IMAGE** |
| **Phylum:** Cnidaria  **Class:** Anthozoa  **Subclass:** Octocorallia  **Order:** Alcyonacea  **Family:** Acanthogorgiidae  **Genus:** *Acanthogorgia*  **Species:** | **Morphospecies code:** ACANT1  *Acanthogorgia* sp. | **Gear:**  ROV  **Photo by:**  *Holland I* 2018  **Identified by:**  Renata Arantes  **Dive: 7** |  |  |
| **Phylum:** Cnidaria  **Class:** Anthozoa  **Subclass:** Octocorallia  **Order:** Alcyonacea  **Family:**  **Genus:**  **Species:** | **Morphospecies code:** ALCYONACEA1 | **Gear:**  ROV  **Photo by:**  *Holland I* 2018  **Identified by:**  Poppy Keogh  **Dive: 8** |  |  |
| **Phylum:** Cnidaria  **Class:** Anthozoa  **Subclass:** Octocorallia  **Order:** Alcyonacea  **Family:**  **Genus:**  **Species:** | **Morphospecies code:** ALCYONACEA2 | **Gear:**  ROV  **Photo by:**  *Holland I* 2018  **Identified by:**  Poppy Keogh  **Dive: 9** |  |  |
| **Phylum:** Cnidaria  **Class:** Anthozoa  **Subclass:** Octocorallia  **Order:** Alcyonacea  **Family:**  **Genus:**  **Species:** | **Morphospecies code:** ALCYONACEA3 | **Gear:**  ROV  **Photo by:**  *Holland I* 2018  **Identified by:**  Poppy Keogh  **Dive: 6** |  |  |
| **TAXONOMY** | **NAME** | **HABITAT** | **NOTES** | **IMAGE** |
| **Phylum:** Cnidaria  **Class:** Anthozoa  **Subclass:** Octocorallia  **Order:** Alcyonacea  **Family:** Alcyoniidae  **Genus:** *Anthomastus*  **Species:** | **Morphospecies code:** ANTHO1  *Anthomastus* sp. | **Gear:**  ROV  **Photo by:**  *Holland I* 2018  **Identified by:**  Poppy Keogh  **Dive: 7** |  |  |
| **Phylum:** Cnidaria  **Class:** Anthozoa  **Subclass:** Octocorallia  **Order:** Alcyonacea  **Family:** Alcyoniidae  **Genus:** *Anthomastus*  **Species:** | **Morphospecies code:** ANTHO2  *Anthomastus* sp. | **Gear:**  ROV  **Photo by:**  *Holland I* 2018  **Identified by:**  Poppy Keogh  **Dive: 7** |  |  |
| **Phylum:** Cnidaria  **Class:** Anthozoa  **Subclass:** Octocorallia  **Order:** Alcyonacea  **Family:** Alcyoniidae  **Genus:** *Anthomastus*  **Species:** | **Morphospecies code:** ANTHO3  *Anthomastus* sp. | **Gear:**  ROV  **Photo by:**  *Holland I* 2018  **Identified by:**  Poppy Keogh  **Dive: 7** |  |  |
| **Phylum:** Cnidaria  **Class:** Anthozoa  **Subclass:** Octocorallia  **Order:** Alcyonacea  **Family:** Chrysogorgiidae  **Genus:** *Chrysogorgia*  **Species:** | **Morphospecies code:**  CRYSOG1  *Chrysogorgia* sp | **Gear:**  ROV  **Photo by:**  *Holland I* 2018  **Identified by:**  Renata Arantes  **Dive: 9** |  |  |
| **TAXONOMY** | **NAME** | **HABITAT** | **NOTES** | **IMAGE** |
| **Phylum:** Cnidaria  **Class:** Anthozoa  **Subclass:** Octocorallia  **Order:** Alcyonacea  **Family:** Chrysogorgiidae  **Genus:** *Chrysogorgia*  **Species:** | **Morphospecies code:**  CRYSOG2  *Chrysogorgia* sp | **Gear:**  ROV  **Photo by:**  *Holland I* 2018  **Identified by:**  Renata Arantes  **Dive: 9** |  |  |
| **Phylum:** Cnidaria  **Class:** Anthozoa  **Subclass:** Octocorallia  **Order:** Alcyonacea  **Family:** Chrysogorgiidae  **Genus:** *Chrysogorgia*  **Species:** | **Morphospecies code:**  CRYSOG3  *Chrysogorgia* sp | **Gear:**  ROV  **Photo by:**  *Holland I* 2018  **Identified by:**  Renata Arantes  **Dive: 8** |  |  |
| **Phylum:** Cnidaria  **Class:** Anthozoa  **Subclass:** Octocorallia  **Order:** Alcyonacea  **Family:** Chrysogorgiidae  **Genus:** *Iridogorgia*  **Species:** | **Morphospecies code:**  IRIDOG1  *Iridogorgia* sp | **Gear:**  ROV  **Photo by:**  *Holland I* 2018  **Identified by:**  Renata Arantes  **Dive: 9** |  |  |
| **Phylum:** Cnidaria  **Class:** Anthozoa  **Subclass:** Octocorallia  **Order:** Alcyonacea  **Family:** Clavulariidae  **Genus:** *Clavularia*  **Species:** | **Morphospecies code:**  CLAVU1  *Clavularia* sp. | **Gear:**  ROV  **Photo by:**  *Holland I* 2018  **Identified by:**  Renata Arantes  **Dive: 6** |  |  |
| **TAXONOMY** | **NAME** | **HABITAT** | **NOTES** | **IMAGE** |
| **Phylum:** Cnidaria  **Class:** Anthozoa  **Subclass:** Octocorallia  **Order:** Alcyonacea  **Family:** Isididae  **Genus:** *Acanella*  **Species:** | **Morphospecies code:**  ACANEL1  *Acanella* sp. | **Gear:**  ROV  **Photo by:**  *Holland I* 2018  **Identified by:**  Renata Arantes  **Dive: 7** |  |  |
| **Phylum:** Cnidaria  **Class:** Anthozoa  **Subclass:** Octocorallia  **Order:** Alcyonacea  **Family:** Isididae  **Genus:**  **Species:** | **Morphospecies code:**  ISIDIDAE1 | **Gear:**  ROV  **Photo by:**  *Holland I* 2018  **Identified by:**  Poppy Keogh  **Dive: 6** |  |  |
| **Phylum:** Cnidaria  **Class:** Anthozoa  **Subclass:** Octocorallia  **Order:** Alcyonacea  **Family:** Isididae  **Genus:**  **Species:** | **Morphospecies code:**  ISIDIDAE2 | **Gear:**  ROV  **Photo by:**  *Holland I* 2018  **Identified by:**  Poppy Keogh  **Dive: 6** |  |  |
| **Phylum:** Cnidaria  **Class:** Anthozoa  **Subclass:** Octocorallia  **Order:** Alcyonacea  **Family:** Isididae  **Genus:**  **Species:** | **Morphospecies code:**  ISIDIDAE3 | **Gear:**  ROV  **Photo by:**  *Holland I* 2018  **Identified by:**  Poppy Keogh  **Dive: 6** |  |  |
| **TAXONOMY** | **NAME** | **HABITAT** | **NOTES** | **IMAGE** |
| **Phylum:** Cnidaria  **Class:** Anthozoa  **Subclass:** Octocorallia  **Order:** Alcyonacea  **Family:** Isididae  **Genus:**  **Species:** | **Morphospecies code:**  ISIDIDAE4 | **Gear:**  ROV  **Photo by:**  *Holland I* 2018  **Identified by:**  Poppy Keogh  **Dive: 5** |  |  |
| **Phylum:** Cnidaria  **Class:** Anthozoa  **Subclass:** Octocorallia  **Order:** Alcyonacea  **Family:** Isididae  **Genus:**  **Species:** | **Morphospecies code:**  ISIDIDAE5 | **Gear:**  ROV  **Photo by:**  *Holland I* 2018  **Identified by:**  Poppy Keogh  **Dive: 6** |  |  |
| **Phylum:** Cnidaria  **Class:** Anthozoa  **Subclass:** Octocorallia  **Order:** Alcyonacea  **Family:** Isididae  **Genus:**  **Species:** | **Morphospecies code:**  ISIDIDAE7 | **Gear:**  ROV  **Photo by:**  *Holland I* 2018  **Identified by:**  Poppy Keogh  **Dive: 7** |  |  |
| **Phylum:** Cnidaria  **Class:** Anthozoa  **Subclass:** Octocorallia  **Order:** Alcyonacea  **Family:** Isididae  **Genus:**  **Species:** | **Morphospecies code:**  ISIDIDAE9 | **Gear:**  ROV  **Photo by:**  *Holland I* 2018  **Identified by:**  Poppy Keogh  **Dive: 6** | Probably the same as ISIDIDAE5 (Poppy Keogh) |  |
| **TAXONOMY** | **NAME** | **HABITAT** | **NOTES** | **IMAGE** |
| **Phylum:** Cnidaria  **Class:** Anthozoa  **Subclass:** Octocorallia  **Order:** Alcyonacea  **Family:** Isididae  **Genus:**  **Species:** | **Morphospecies code:**  ISIDIDAE10 | **Gear:**  ROV  **Photo by:**  *Holland I* 2018  **Identified by:**  Poppy Keogh  **Dive: 5** | Probably the same as ISIDIDAE4 (Poppy Keogh) |  |
| **Phylum:** Cnidaria  **Class:** Anthozoa  **Subclass:** Octocorallia  **Order:** Alcyonacea  **Family:** Isididae  **Genus:**  **Species:** | **Morphospecies code:**  ISIDIDAE11 | **Gear:**  ROV  **Photo by:**  *Holland I* 2018  **Identified by:**  Poppy Keogh  **Dive: 7** |  |  |
| **Phylum:** Cnidaria  **Class:** Anthozoa  **Subclass:** Octocorallia  **Order:** Alcyonacea  **Family:** Paragorgiidae  **Genus:**  **Species:** | **Morphospecies code:** PARAGO1 | **Gear:**  ROV  **Photo by:**  *Holland I* 2018  **Identified by:**  Poppy Keogh  **Dive: 9** |  |  |
| **Phylum:** Cnidaria  **Class:** Anthozoa  **Subclass:** Octocorallia  **Order:** Alcyonacea  **Family:** Paragorgiidae  **Genus:**  **Species:** | **Morphospecies code:** PARAGO5 | **Gear:**  ROV  **Photo by:**  *Holland I* 2018  **Identified by:**  Poppy Keogh  **Dive: 6** |  |  |
| **TAXONOMY** | **NAME** | **HABITAT** | **NOTES** | **IMAGE** |
| **Phylum:** Cnidaria  **Class:** Anthozoa  **Subclass:** Octocorallia  **Order:** Alcyonacea  **Family:** Paragorgiidae  **Genus:**  **Species:** | **Morphospecies code:** PARAGO6 | **Gear:**  ROV  **Photo by:**  *Holland I* 2018  **Identified by:**  Poppy Keogh  **Dive: 7** |  |  |
| **Phylum:** Cnidaria  **Class:** Anthozoa  **Subclass:** Octocorallia  **Order:** Alcyonacea  **Family:** Paragorgiidae  **Genus:**  **Species:** | **Morphospecies code:** PARAGO7 | **Gear:**  ROV  **Photo by:**  *Holland I* 2018  **Identified by:**  Poppy Keogh  **Dive: 9** |  |  |
| **Phylum:** Cnidaria  **Class:** Anthozoa  **Subclass:** Octocorallia  **Order:** Alcyonacea  **Family:** Paragorgiidae  **Genus:**  **Species:** | **Morphospecies code:** PARAGO8 | **Gear:**  ROV  **Photo by:**  *Holland I* 2018  **Identified by:**  Poppy Keogh  **Dive: 8** |  |  |
| **Phylum:** Cnidaria  **Class:** Anthozoa  **Subclass:** Octocorallia  **Order:** Alcyonacea  **Family:** Plexauridae  **Genus:**  **Species:** | **Morphospecies code:** PLEXAURIDAE1 | **Gear:**  ROV  **Photo by:**  *Holland I* 2018  **Identified by:**  Poppy Keogh  **Dive: 6** |  |  |
| **TAXONOMY** | **NAME** | **HABITAT** | **NOTES** | **IMAGE** |
| **Phylum:** Cnidaria  **Class:** Anthozoa  **Subclass:** Octocorallia  **Order:** Alcyonacea  **Family:** Plexauridae  **Genus:**  **Species:** | **Morphospecies code:** PLEXAURIDAE2 | **Gear:**  ROV  **Photo by:**  *Holland I* 2018  **Identified by:**  Renata Arantes  **Dive: 9** |  |  |
| **Phylum:** Cnidaria  **Class:** Anthozoa  **Subclass:** Octocorallia  **Order:** Alcyonacea  **Family:** Primnoidae  **Genus:** *Calyptrophora*  **Species:** | **Morphospecies code:** CALYPT1  *Calyptrophora* sp. | **Gear:**  ROV  **Photo by:**  *Holland I* 2018  **Identified by:**  Renata Arantes  **Dive: 9** |  |  |
| **Phylum:** Cnidaria  **Class:** Anthozoa  **Subclass:** Octocorallia  **Order:** Alcyonacea  **Family:** Primnoidae  **Genus:**  **Species:** | **Morphospecies code:** PRIMNO1 | **Gear:**  ROV  **Photo by:**  *Holland I* 2018  **Identified by:**  Renata Arantes  **Dive: 9** |  |  |
| **Phylum:** Cnidaria  **Class:** Anthozoa  **Subclass:** Octocorallia  **Order:** Pennatulacea  **Family:**  **Genus:**  **Species:** | **Morphospecies code:** PENNATULA1 | **Gear:**  ROV  **Photo by:**  *Holland I* 2018  **Identified by:**  Poppy Keogh  **Dive: 5** |  |  |
| **TAXONOMY** | **NAME** | **HABITAT** | **NOTES** | **IMAGE** |
| **Phylum:** Cnidaria  **Class:** Anthozoa  **Subclass:** Octocorallia  **Order:** Pennatulacea  **Family:** Anthoptilidae  **Genus:** *Anthoptilum*  **Species:** *A. grandiflorum* | **Morphospecies code:** PENNATULA2  *Anthoptilum* cf. *grandiflorum* | **Gear:**  ROV  **Photo by:**  *Holland I* 2018  **Identified by:**  Raissa Hogan  **Dive: 7** |  |  |
| **Phylum:** Cnidaria  **Class:** Anthozoa  **Subclass:** Octocorallia  **Order:** Pennatulacea  **Family:** Anthoptilidae  **Genus:** *Anthoptilum*  **Species:** | **Morphospecies code:** PENNATULA3  *Anthoptilum* sp. | **Gear:**  ROV  **Photo by:**  *Holland I* 2018  **Identified by:**  Raissa Hogan  **Dive: 8** |  |  |
| **Phylum:** Cnidaria  **Class:** Anthozoa  **Subclass:** Octocorallia  **Order:** Pennatulacea  **Family:**  **Genus:**  **Species:** | **Morphospecies code:** PENNATULA4 | **Gear:**  ROV  **Photo by:**  *Holland I* 2018  **Identified by:**  Poppy Keogh  **Dive: 7** |  |  |
| **Phylum:** Cnidaria  **Class:** Anthozoa  **Subclass:** Octocorallia  **Order:** Pennatulacea  **Family:**  **Genus:**  **Species:** | **Morphospecies code:** PENNATULA6 | **Gear:**  ROV  **Photo by:**  *Holland I* 2018  **Identified by:**  Poppy Keogh  **Dive: 7** |  |  |
| **TAXONOMY** | **NAME** | **HABITAT** | **NOTES** | **IMAGE** |
| **Phylum:** Cnidaria  **Class:** Anthozoa  **Subclass:** Octocorallia  **Order:** Pennatulacea  **Family:** Anthoptilidae  **Genus:** *Anthoptilum*  **Species:** | **Morphospecies code:** PENNATULA8  *Anthoptilum* sp. | **Gear:**  ROV  **Photo by:**  *Holland I* 2018  **Identified by:**  Raissa Hogan  **Dive: 7** |  |  |
| **Phylum:** Cnidaria  **Class:** Anthozoa  **Subclass:** Octocorallia  **Order:** Pennatulacea  **Family:** Halipteridae  **Genus:** *Halipteris*  **Species:** *H. finmarchica* | **Morphospecies code:** PENNATULA9  *Halipteris* cf. *finmarchica* | **Gear:**  ROV  **Photo by:**  *Holland I* 2018  **Identified by:**  Raissa Hogan  **Dive: 7** |  |  |
| **Phylum:** Cnidaria  **Class:** Anthozoa  **Subclass:** Octocorallia  **Order:** Pennatulacea  **Family:**  **Genus:**  **Species:** | **Morphospecies code:** PENNATULA10  *Pennatula* sp. or *Ptilella* | **Gear:**  ROV  **Photo by:**  *Holland I* 2018  **Identified by:**  Raissa Hogan  **Dive: 5** |  |  |
| **Phylum:** Cnidaria  **Class:** Anthozoa  **Subclass:** Octocorallia  **Order:** Pennatulacea  **Family:**  **Genus:**  **Species:** | **Morphospecies code:** PENNATULA11 | **Gear:**  ROV  **Photo by:**  *Holland I* 2018  **Identified by:**  Poppy Keogh  **Dive: 7** |  |  |
| **TAXONOMY** | **NAME** | **HABITAT** | **NOTES** | **IMAGE** |
| **Phylum:** Cnidaria  **Class:** Anthozoa  **Subclass:** Octocorallia  **Order:** Pennatulacea  **Family:**  **Genus:**  **Species:** | **Morphospecies code:** PENNATULA12 | **Gear:**  ROV  **Photo by:**  *Holland I* 2018  **Identified by:**  Poppy Keogh  **Dive: 7** |  |  |
| **Phylum:** Cnidaria  **Class:** Anthozoa  **Subclass:** Octocorallia  **Order:** Pennatulacea  **Family:**  **Genus:**  **Species:** | **Morphospecies code:** PENNATULA16 | **Gear:**  ROV  **Photo by:**  *Holland I* 2018  **Identified by:**  Poppy Keogh  **Dive: 7** |  |  |
| **Phylum:** Cnidaria  **Class:** Anthozoa  **Subclass:** Octocorallia  **Order:** Pennatulacea  **Family:** Halipteridae  **Genus:** *Halipteris*  **Species:** *finmarchica* | **Morphospecies code:** PENNATULA17  *Halipteris* cf. *finmarchica* | **Gear:**  ROV  **Photo by:**  *Holland I* 2018  **Identified by:**  Raissa Hogan  **Dive: 7** | Probably the same as PENNATULA9 (P Keogh) |  |
| **Phylum:** Cnidaria  **Class:** Anthozoa  **Subclass:** Octocorallia  **Order:** Pennatulacea  **Family:** Halipteridae  **Genus:** *Halipteris*  **Species:** | **Morphospecies code:** PENNATULA18  *Halipteris* sp. | **Gear:**  ROV  **Photo by:**  *Holland I* 2018  **Identified by:**  Raissa Hogan  **Dive: 7** | Probably the same as PENNATULA9 (P Keogh) |  |
| **TAXONOMY** | **NAME** | **HABITAT** | **NOTES** | **IMAGE** |
| **Phylum:** Cnidaria  **Class:** Anthozoa  **Subclass:** Octocorallia  **Order:** Pennatulacea  **Family:**  **Genus:**  **Species:** | **Morphospecies code:** PENNATULA19 | **Gear:**  ROV  **Photo by:**  *Holland I* 2018  **Identified by:**  Poppy Keogh  **Dive: 9** |  |  |
| **Phylum:** Cnidaria  **Class:** Anthozoa  **Subclass:** Octocorallia  **Order:** Pennatulacea  **Family:** Umbellulidae  **Genus:** *Umbellula*  **Species:** | **Morphospecies code:** UMBELLULA1  *Umbellula* sp. | **Gear:**  ROV  **Photo by:**  *Holland I* 2018  **Identified by:**  Raissa Hogan  **Dive: 6** |  |  |
| **Phylum:** Cnidaria  **Class:**  **Subclass:**  **Order:**  **Family:**  **Genus:**  **Species:** | **Morphospecies code:** CNID1 | **Gear:**  ROV  **Photo by:**  *Holland I* 2018  **Identified by:**  Poppy Keogh  **Dive: 5** |  |  |
| **Phylum:** Cnidaria  **Class:**  **Subclass:**  **Order:**  **Family:**  **Genus:**  **Species:** | **Morphospecies code:** CNID3 | **Gear:**  ROV  **Photo by:**  *Holland I* 2018  **Identified by:**  Poppy Keogh  **Dive: 9** |  |  |
| **Phylum:** Cnidaria  **Class:**  **Subclass:**  **Order:**  **Family:**  **Genus:**  **Species:** | **Morphospecies code:** CNID7 | **Gear:**  ROV  **Photo by:**  *Holland I* 2018  **Identified by:**  Poppy Keogh  **Dive: 5** |  |  |
| **TAXONOMY** | **NAME** | **HABITAT** | **NOTES** | **IMAGE** |
| **Phylum:** Cnidaria  **Class:**  **Subclass:**  **Order:**  **Family:**  **Genus:**  **Species:** | **Morphospecies code:** CNID8 | **Gear:**  ROV  **Photo by:**  *Holland I* 2018  **Identified by:**  Poppy Keogh  **Dive: 5** |  |  |
| **Phylum:** Cnidaria  **Class:**  **Subclass:**  **Order:**  **Family:**  **Genus:**  **Species:** | **Morphospecies code:** CNID9 | **Gear:**  ROV  **Photo by:**  *Holland I* 2018  **Identified by:**  Poppy Keogh  **Dive: 9** |  |  |
| **Phylum:** Cnidaria  **Class:**  **Subclass:**  **Order:**  **Family:**  **Genus:**  **Species:** | **Morphospecies code:** CNID10 | **Gear:**  ROV  **Photo by:**  *Holland I* 2018  **Identified by:**  Poppy Keogh  **Dive: 9** |  |  |
| **Phylum:** Cnidaria  **Class:**  **Subclass:**  **Order:**  **Family:**  **Genus:**  **Species:** | **Morphospecies code:** CNID11 | **Gear:**  ROV  **Photo by:**  *Holland I* 2018  **Identified by:**  Poppy Keogh  **Dive: 9** |  |  |
| **Phylum:** Cnidaria  **Class:** Hydrozoa  **Subclass:**  **Order:**  **Family:**  **Genus:**  **Species:** | **Morphospecies code:** HYDRO1 | **Gear:**  ROV  **Photo by:**  *Holland I* 2018  **Identified by:**  Poppy Keogh  **Dive: 9** |  |  |
| **Phylum:** Cnidaria  **Class:** Hydrozoa  **Subclass:**  **Order:**  **Family:**  **Genus:**  **Species:** | **Morphospecies code:** HYDRO2 | **Gear:**  ROV  **Photo by:**  *Holland I* 2018  **Identified by:**  Poppy Keogh  **Dive: 8** | Probably the same as HYDRO1 |  |
| **TAXONOMY** | **NAME** | **HABITAT** | **NOTES** | **IMAGE** |
| **Phylum:** Echinodermata  **Class:** Asteroidea  **Subclass:**  **Order:**  **Family:**  **Genus:**  **Species:** | **Morphospecies code:** ASTERO1 | **Gear:**  ROV  **Photo by:**  *Holland I* 2018  **Identified by:**  Poppy Keogh  **Dive: 5** | Possibly a Brisingidae (*H. coronate* / *B. endecacnemos*) (P. Keogh) |  |
| **Phylum:** Echinodermata  **Class:** Asteroidea  **Subclass:**  **Order:**  **Family:**  **Genus:**  **Species:** | **Morphospecies code:** ASTERO2 | **Gear:**  ROV  **Photo by:**  *Holland I* 2018  **Identified by:**  Poppy Keogh  **Dive: 5** | Possibly *Henricia sanguinolenta* (P. Keogh) |  |
| **Phylum:** Echinodermata  **Class:** Asteroidea  **Subclass:**  **Order:**  **Family:**  **Genus:**  **Species:** | **Morphospecies code:** ASTERO3 | **Gear:**  ROV  **Photo by:**  *Holland I* 2018  **Identified by:**  Poppy Keogh  **Dive: 5** |  |  |
| **Phylum:** Echinodermata  **Class:** Asteroidea  **Subclass:**  **Order:**  **Family:**  **Genus:**  **Species:** | **Morphospecies code:** ASTERO4 | **Gear:**  ROV  **Photo by:**  *Holland I* 2018  **Identified by:**  Poppy Keogh  **Dive: 5** | Possibly *Porania pulvillis* (P. Keogh) |  |
| **Phylum:** Echinodermata  **Class:** Asteroidea  **Subclass:**  **Order:**  **Family:**  **Genus:**  **Species:** | **Morphospecies code:** ASTERO6 | **Gear:**  ROV  **Photo by:**  *Holland I* 2018  **Identified by:**  Poppy Keogh  **Dive: 6** | Possibly *Porania pulvillis* (P. Keogh) |  |
| **TAXONOMY** | **NAME** | **HABITAT** | **NOTES** | **IMAGE** |
| **Phylum:** Echinodermata  **Class:** Asteroidea  **Subclass:**  **Order:**  **Family:**  **Genus:**  **Species:** | **Morphospecies code:** ASTERO7 | **Gear:**  ROV  **Photo by:**  *Holland I* 2018  **Identified by:**  Poppy Keogh  **Dive: 7** |  |  |
| **Phylum:** Echinodermata  **Class:** Asteroidea  **Subclass:**  **Order:**  **Family:**  **Genus:**  **Species:** | **Morphospecies code:** ASTERO9 | **Gear:**  ROV  **Photo by:**  *Holland I* 2018  **Identified by:**  Poppy Keogh  **Dive: 6** | Possibly *Hymenaster* sp. (P. Keogh) |  |
| **Phylum:** Echinodermata  **Class:** Asteroidea  **Subclass:**  **Order:**  **Family:**  **Genus:**  **Species:** | **Morphospecies code:** ASTERO10 | **Gear:**  ROV  **Photo by:**  *Holland I* 2018  **Identified by:**  Poppy Keogh  **Dive: 7** |  |  |
| **Phylum:** Echinodermata  **Class:** Asteroidea  **Subclass:**  **Order:**  **Family:**  **Genus:**  **Species:** | **Morphospecies code:** ASTERO11 | **Gear:**  ROV  **Photo by:**  *Holland I* 2018  **Identified by:**  Poppy Keogh  **Dive: 6** |  |  |
| **Phylum:** Echinodermata  **Class:** Asteroidea  **Subclass:**  **Order:**  **Family:**  **Genus:**  **Species:** | **Morphospecies code:** ASTERO12 | **Gear:**  ROV  **Photo by:**  *Holland I* 2018  **Identified by:**  Poppy Keogh  **Dive: 6** |  |  |
| **TAXONOMY** | **NAME** | **HABITAT** | **NOTES** | **IMAGE** |
| **Phylum:** Echinodermata  **Class:** Asteroidea  **Subclass:**  **Order:**  **Family:**  **Genus:**  **Species:** | **Morphospecies code:** ASTERO13 | **Gear:**  ROV  **Photo by:**  *Holland I* 2018  **Identified by:**  Poppy Keogh  **Dive: 6** |  |  |
| **Phylum:** Echinodermata  **Class:** Asteroidea  **Subclass:**  **Order:**  **Family:**  **Genus:**  **Species:** | **Morphospecies code:** ASTERO14 | **Gear:**  ROV  **Photo by:**  *Holland I* 2018  **Identified by:**  Poppy Keogh  **Dive: 7** |  |  |
| **Phylum:** Echinodermata  **Class:** Asteroidea  **Subclass:**  **Order:**  **Family:**  **Genus:**  **Species:** | **Morphospecies code:** ASTERO18 | **Gear:**  ROV  **Photo by:**  *Holland I* 2018  **Identified by:**  Poppy Keogh  **Dive: 5** |  |  |
| **Phylum:** Echinodermata  **Class:** Asteroidea  **Subclass:**  **Order:**  **Family:**  **Genus:**  **Species:** | **Morphospecies code:** ASTERO19 | **Gear:**  ROV  **Photo by:**  *Holland I* 2018  **Identified by:**  Poppy Keogh  **Dive: 8** |  |  |
| **Phylum:** Echinodermata  **Class:** Asteroidea  **Subclass:**  **Order:**  **Family:**  **Genus:**  **Species:** | **Morphospecies code:** ASTERO20 | **Gear:**  ROV  **Photo by:**  *Holland I* 2018  **Identified by:**  Poppy Keogh  **Dive: 8** |  |  |
| **TAXONOMY** | **NAME** | **HABITAT** | **NOTES** | **IMAGE** |
| **Phylum:** Echinodermata  **Class:** Asteroidea  **Subclass:**  **Order:**  **Family:**  **Genus:**  **Species:** | **Morphospecies code:** ASTERO21 | **Gear:**  ROV  **Photo by:**  *Holland I* 2018  **Identified by:**  Poppy Keogh  **Dive: 8** |  |  |
| **Phylum:** Echinodermata  **Class:** Asteroidea  **Subclass:**  **Order:**  **Family:**  **Genus:**  **Species:** | **Morphospecies code:** ASTERO22 | **Gear:**  ROV  **Photo by:**  *Holland I* 2018  **Identified by:**  Poppy Keogh  **Dive: 8** |  |  |
| **Phylum:** Echinodermata  **Class:** Asteroidea  **Subclass:**  **Order:**  **Family:**  **Genus:**  **Species:** | **Morphospecies code:** ASTERO23 | **Gear:**  ROV  **Photo by:**  *Holland I* 2018  **Identified by:**  Poppy Keogh  **Dive: 9** |  |  |
| **Phylum:** Echinodermata  **Class:** Asteroidea  **Subclass:**  **Order:**  **Family:**  **Genus:**  **Species:** | **Morphospecies code:** ASTERO24 | **Gear:**  ROV  **Photo by:**  *Holland I* 2018  **Identified by:**  Poppy Keogh  **Dive: 7** |  |  |
| **Phylum:** Echinodermata  **Class:** Asteroidea  **Subclass:**  **Order:**  **Family:**  **Genus:**  **Species:** | **Morphospecies code:** ASTERO25 | **Gear:**  ROV  **Photo by:**  *Holland I* 2018  **Identified by:**  Poppy Keogh  **Dive:** |  |  |
| **TAXONOMY** | **NAME** | **HABITAT** | **NOTES** | **IMAGE** |
| **Phylum:** Echinodermata  **Class:** Asteroidea  **Subclass:**  **Order:** Brisingida  **Family:** Brisingidae  **Genus:**  **Species:** | **Morphospecies code:** BRISIN1 | **Gear:**  ROV  **Photo by:**  *Holland I* 2018  **Identified by:**  Poppy Keogh  **Dive: 8** | Possibly *Novodinia* sp. (P. Keogh) |  |
| **Phylum:** Echinodermata  **Class:** Crinoidea  **Subclass:**  **Order:** Hyocrinida  **Family:** Hyocrinidae  **Genus:** *Anachalypsicrinus*  **Species:** *A. nefertiti* | **Morphospecies code:** ANACH  *Anachalypsicrinus nefertiti* | **Gear:**  ROV  **Photo by:**  *Holland I* 2018  **Identified by:**  Poppy Keogh  **Dive: 6** |  |  |
| **Phylum:** Echinodermata  **Class:** Crinoidea  **Subclass:**  **Order:**  **Family:**  **Genus:**  **Species:** | **Morphospecies code:** CRINO3 | **Gear:**  ROV  **Photo by:**  *Holland I* 2018  **Identified by:**  Poppy Keogh  **Dive: 5** |  |  |
| **Phylum:** Echinodermata  **Class:** Crinoidea  **Subclass:**  **Order:**  **Family:**  **Genus:**  **Species:** | **Morphospecies code:** CRINO4 | **Gear:**  ROV  **Photo by:**  *Holland I* 2018  **Identified by:**  Poppy Keogh  **Dive: 5** |  |  |
| **TAXONOMY** | **NAME** | **HABITAT** | **NOTES** | **IMAGE** |
| **Phylum:** Echinodermata  **Class:** Crinoidea  **Subclass:**  **Order:** Comatulida  **Family:** Bathycrinidae  **Genus:** *Bathycrinidae*  **Species:** | **Morphospecies code:** CRINO6  *Bathycrinidae* sp. | **Gear:**  ROV  **Photo by:**  *Holland I* 2018  **Identified by:**  Poppy Keogh  **Dive: 7** |  |  |
| **Phylum:** Echinodermata  **Class:** Crinoidea  **Subclass:**  **Order:**  **Family:**  **Genus:**  **Species:** | **Morphospecies code:** CRINO9 | **Gear:**  ROV  **Photo by:**  *Holland I* 2018  **Identified by:**  Poppy Keogh  **Dive: 6** |  |  |
| **Phylum:** Echinodermata  **Class:** Crinoidea  **Subclass:**  **Order:**  **Family:**  **Genus:**  **Species:** | **Morphospecies code:** CRINO10 | **Gear:**  ROV  **Photo by:**  *Holland I* 2018  **Identified by:**  Poppy Keogh  **Dive: 6** |  |  |
| **Phylum:** Echinodermata  **Class:** Crinoidea  **Subclass:**  **Order:**  **Family:**  **Genus:**  **Species:** | **Morphospecies code:** CRINO11 | **Gear:**  ROV  **Photo by:**  *Holland I* 2018  **Identified by:**  Poppy Keogh  **Dive: 6** |  |  |
| **Phylum:** Echinodermata  **Class:** Crinoidea  **Subclass:**  **Order:**  **Family:**  **Genus:**  **Species:** | **Morphospecies code:** CRINO12 | **Gear:**  ROV  **Photo by:**  *Holland I* 2018  **Identified by:**  Poppy Keogh  **Dive: 6** |  |  |
| **TAXONOMY** | **NAME** | **HABITAT** | **NOTES** | **IMAGE** |
| **Phylum:** Echinodermata  **Class:** Crinoidea  **Subclass:**  **Order:**  **Family:**  **Genus:**  **Species:** | **Morphospecies code:** CRINO13 | **Gear:**  ROV  **Photo by:**  *Holland I* 2018  **Identified by:**  Poppy Keogh  **Dive: 6** |  |  |
| **Phylum:** Echinodermata  **Class:** Crinoidea  **Subclass:**  **Order:**  **Family:**  **Genus:**  **Species:** | **Morphospecies code:** CRINO14 | **Gear:**  ROV  **Photo by:**  *Holland I* 2018  **Identified by:**  Poppy Keogh  **Dive: 6** |  |  |
| **Phylum:** Echinodermata  **Class:** Crinoidea  **Subclass:**  **Order:**  **Family:**  **Genus:**  **Species:** | **Morphospecies code:** CRINO15 | **Gear:**  ROV  **Photo by:**  *Holland I* 2018  **Identified by:**  Poppy Keogh  **Dive: 6** |  |  |
| **Phylum:** Echinodermata  **Class:** Crinoidea  **Subclass:**  **Order:** Comatulida  **Family:** Pentametrocrinidae  **Genus:** *Pentametrocrinus*  **Species:** *P. atlanticus* | **Morphospecies code:** PENTA  *Pentametrocrinus atlanticus* | **Gear:**  ROV  **Photo by:**  *Holland I* 2018  **Identified by:**  Poppy Keogh  **Dive: 5** |  |  |
| **TAXONOMY** | **NAME** | **HABITAT** | **NOTES** | **IMAGE** |
| **Phylum:** Echinodermata  **Class:** Crinoidea  **Subclass:**  **Order:** Comatulida  **Family:** Septocrinidae  **Genus:** *Zeuctocrinus*  **Species:** *Z. gisleni* | **Morphospecies code: ZEUCT**  *Zeuctocrinus gisleni* | **Gear:**  ROV  **Photo by:**  *Holland I* 2018  **Identified by:**  Poppy Keogh  **Dive: 5** |  |  |
| **Phylum:** Echinodermata  **Class:** Echinoidea  **Subclass:**  **Order:**  **Family:**  **Genus:**  **Species:** | **Morphospecies code:** ECHINO1 | **Gear:**  ROV  **Photo by:**  *Holland I* 2018  **Identified by:**  Poppy Keogh  **Dive: 6** | Possibly *Echinothuroidea* sp. |  |
| **Phylum:** Echinodermata  **Class:** Echinoidea  **Subclass:**  **Order:**  **Family:**  **Genus:**  **Species:** | **Morphospecies code:** ECHINO2 | **Gear:**  ROV  **Photo by:**  *Holland I* 2018  **Identified by:**  Poppy Keogh  **Dive: 6** |  |  |
| **Phylum:** Echinodermata  **Class:** Echinoidea  **Subclass:**  **Order:**  **Family:**  **Genus:**  **Species:** | **Morphospecies code:** ECHINO3 | **Gear:**  ROV  **Photo by:**  *Holland I* 2018  **Identified by:**  Poppy Keogh  **Dive: 7** |  |  |
| **Phylum:** Echinodermata  **Class:** Echinoidea  **Subclass:**  **Order:**  **Family:**  **Genus:** *Echinus*  **Species:** | **Morphospecies code:** ECHINO4  cf. *Echinus* dp. | **Gear:**  ROV  **Photo by:**  *Holland I* 2018  **Identified by:**  Poppy Keogh  **Dive: 5** |  |  |
| **TAXONOMY** | **NAME** | **HABITAT** | **NOTES** | **IMAGE** |
| **Phylum:** Echinodermata  **Class:** Echinoidea  **Subclass:**  **Order:**  **Family:**  **Genus:**  **Species:** | **Morphospecies code:** ECHINO5 | **Gear:**  ROV  **Photo by:**  *Holland I* 2018  **Identified by:**  Poppy Keogh  **Dive: 7** |  |  |
| **Phylum:** Echinodermata  **Class:** Echinoidea  **Subclass:**  **Order:**  **Family:**  **Genus:**  **Species:** | **Morphospecies code:** ECHINO6 | **Gear:**  ROV  **Photo by:**  *Holland I* 2018  **Identified by:**  Poppy Keogh  **Dive: 8** |  |  |
| **Phylum:** Echinodermata  **Class:** Holothuroidea  **Subclass:** Actinopoda  **Order:** Synallactida  **Family:** Synallactidae  **Genus:** *Synallactes*  **Species:** | **Morphospecies code:** HOLO1  cf. *Synallactes* sp. | **Gear:**  ROV  **Photo by:**  *Holland I* 2018  **Identified by:**  Poppy Keogh  **Dive: 5** |  |  |
| **Phylum:** Echinodermata  **Class:** Holothuroidea  **Subclass:**  **Order:**  **Family:**  **Genus:** *Psolus*  **Species:** | **Morphospecies code:** HOLO2  *Psolus* sp. | **Gear:**  ROV  **Photo by:**  *Holland I* 2018  **Identified by:**  Poppy Keogh  **Dive: 8** |  |  |
| **Phylum:** Echinodermata  **Class:** Holothuroidea  **Subclass:**  **Order:**  **Family:**  **Genus:**  **Species:** | **Morphospecies code:** HOLO4 | **Gear:**  ROV  **Photo by:**  *Holland I* 2018  **Identified by:**  Poppy Keogh  **Dive: 6** |  |  |
| **TAXONOMY** | **NAME** | **HABITAT** | **NOTES** | **IMAGE** |
| **Phylum:** Echinodermata  **Class:** Holothuroidea  **Subclass:**  Actinopoda  **Order:** *Elasipodida*  **Family:** Laetmogonidae  **Genus:** *Benthogone*  **Species:** | **Morphospecies code:** HOLO5  cf. *Benthogone* sp. | **Gear:**  ROV  **Photo by:**  *Holland I* 2018  **Identified by:**  Poppy Keogh  **Dive: 7** |  |  |
| **Phylum:** Echinodermata  **Class:** Holothuroidea  **Subclass:** Actinopoda  **Order:** Elasipodida  **Family:** Elpidiidae  **Genus:** *Amperima*  **Species:** | **Morphospecies code:** HOLO6  cf. *Amperima* sp. | **Gear:**  ROV  **Photo by:**  *Holland I* 2018  **Identified by:**  Poppy Keogh  **Dive: 7** |  |  |
| **Phylum:** Echinodermata  **Class:** Holothuroidea  **Subclass:**  **Order:**  **Family:**  **Genus:**  **Species:** | **Morphospecies code:** HOLO7 | **Gear:**  ROV  **Photo by:**  *Holland I* 2018  **Identified by:**  Poppy Keogh  **Dive: 6** |  |  |
| **Phylum:** Echinodermata  **Class:** Holothuroidea  **Subclass:**  **Order:**  **Family:**  **Genus:**  **Species:** | **Morphospecies code:** HOLO8 | **Gear:**  ROV  **Photo by:**  *Holland I* 2018  **Identified by:**  Poppy Keogh  **Dive: 6** |  |  |
| **Phylum:** Echinodermata  **Class:** Holothuroidea  **Subclass:**  **Order:**  **Family:**  **Genus:**  **Species:** | **Morphospecies code:** HOLO10 | **Gear:**  ROV  **Photo by:**  *Holland I* 2018  **Identified by:**  Poppy Keogh  **Dive: 6** |  |  |
| **TAXONOMY** | **NAME** | **HABITAT** | **NOTES** | **IMAGE** |
| **Phylum:** Echinodermata  **Class:** Holothuroidea  **Subclass:**  **Order:**  **Family:**  **Genus:**  **Species:** | **Morphospecies code:** HOLO11 | **Gear:**  ROV  **Photo by:**  *Holland I* 2018  **Identified by:**  Poppy Keogh  **Dive: 8** |  |  |
| **Phylum:** Echinodermata  **Class:** Holothuroidea  **Subclass:**  **Order:**  **Family:**  **Genus:**  **Species:** | **Morphospecies code:** HOLO12 | **Gear:**  ROV  **Photo by:**  *Holland I* 2018  **Identified by:**  Poppy Keogh  **Dive: 8** |  |  |
| **Phylum:** Echinodermata  **Class:** Holothuroidea  **Subclass:** Actinopoda  **Order:** Holothuriida  **Family:** Mesothuriidae  **Genus:** *Mesothuria*  **Species:** *M. intestinalis* | **Morphospecies code:** HOLO13  cf. *Mesothuria intestinalis* | **Gear:**  ROV  **Photo by:**  *Holland I* 2018  **Identified by:**  Poppy Keogh  **Dive: 8** |  |  |
| **Phylum:** Echinodermata  **Class:** Ophiuroidea  **Subclass:**  **Order:**  **Family:**  **Genus:**  **Species:** | **Morphospecies code:** OPHIURO1 | **Gear:**  ROV  **Photo by:**  *Holland I* 2018  **Identified by:**  Poppy Keogh  **Dive: 5** |  |  |
| **Phylum:** Echinodermata  **Class:** Ophiuroidea  **Subclass:**  **Order:**  **Family:**  **Genus:**  **Species:** | **Morphospecies code:** OPHIURO2 | **Gear:**  ROV  **Photo by:**  *Holland I* 2018  **Identified by:**  Poppy Keogh  **Dive: 5** |  |  |
| **TAXONOMY** | **NAME** | **HABITAT** | **NOTES** | **IMAGE** |
| **Phylum:** Echinodermata  **Class:** Ophiuroidea  **Subclass:**  **Order:**  **Family:**  **Genus:**  **Species:** | **Morphospecies code:** OPHIURO3 | **Gear:**  ROV  **Photo by:**  *Holland I* 2018  **Identified by:**  Poppy Keogh  **Dive: 6** |  |  |
| **Phylum:** Echinodermata  **Class:** Ophiuroidea  **Subclass:**  **Order:**  **Family:**  **Genus:**  **Species:** | **Morphospecies code:** OPHIURO4 | **Gear:**  ROV  **Photo by:**  *Holland I* 2018  **Identified by:**  Poppy Keogh  **Dive: 6** |  |  |
| **Phylum:** Echinodermata  **Class:** Ophiuroidea  **Subclass:**  **Order:**  **Family:**  **Genus:**  **Species:** | **Morphospecies code:** OPHIURO5 | **Gear:**  ROV  **Photo by:**  *Holland I* 2018  **Identified by:**  Poppy Keogh  **Dive: 6** |  |  |
| **Phylum:** Echinodermata  **Class:** Ophiuroidea  **Subclass:**  **Order:**  **Family:**  **Genus:**  **Species:** | **Morphospecies code:** OPHIURO7 | **Gear:**  ROV  **Photo by:**  *Holland I* 2018  **Identified by:**  Poppy Keogh  **Dive: 6** |  |  |
| **Phylum:** Echinodermata  **Class:** Ophiuroidea  **Subclass:**  **Order:**  **Family:**  **Genus:**  **Species:** | **Morphospecies code:** OPHIURO8 | **Gear:**  ROV  **Photo by:**  *Holland I* 2018  **Identified by:**  Poppy Keogh  **Dive: 9** |  |  |
| **TAXONOMY** | **NAME** | **HABITAT** | **NOTES** | **IMAGE** |
| **Phylum:** Chordata  **Class:** Ascidiacea  **Order:**  **Family:**  **Genus:**  **Species:** | **Morphospecies code:** ASCID  Ascidiacea spp. | **Gear:**  ROV  **Photo by:**  *Holland I* 2018  **Identified by:** Poppy Keogh  **Dive: 6** |  |  |
| **Phylum:** Chordata  **Class:** Actinopterygii  **Order:**  **Family:**  **Genus:**  **Species:** | **Morphospecies code:** ACTP1 | **Gear:**  ROV  **Photo by:**  *Holland I* 2018  **Identified by:** Poppy Keogh  **Dive: 7** | Blue Hake (P. Keogh) |  |
| **Phylum:** Chordata  **Class:** Actinopterygii  **Order:**  **Family:**  **Genus:**  **Species:** | **Morphospecies code:** ACTP2 | **Gear:**  ROV  **Photo by:**  *Holland I* 2018  **Identified by:** Poppy Keogh  **Dive: 7** |  |  |
| **Phylum:** Chordata  **Class:** Actinopterygii  **Order:**  **Family:**  **Genus:**  **Species:** | **Morphospecies code:** ACTP3 | **Gear:**  ROV  **Photo by:**  *Holland I* 2018  **Identified by:** Poppy Keogh  **Dive: 6** |  |  |
| **Phylum:** Chordata  **Class:** Actinopterygii  **Order:**  **Family:**  **Genus:**  **Species:** | **Morphospecies code:** ACTP4 | **Gear:**  ROV  **Photo by:**  *Holland I* 2018  **Identified by:** Poppy Keogh  **Dive: 6** |  |  |
| **TAXONOMY** | **NAME** | **HABITAT** | **NOTES** | **IMAGE** |
| **Phylum:** Chordata  **Class:** Actinopterygii  **Order:**  **Family:**  **Genus:**  **Species:** | **Morphospecies code:** ACTP5 | **Gear:**  ROV  **Photo by:**  *Holland I* 2018  **Identified by:** Poppy Keogh  **Dive: 5** |  |  |
| **Phylum:** Chordata  **Class:** Actinopterygii  **Order:**  **Family:**  **Genus:**  **Species:** | **Morphospecies code:** ACTP6 | **Gear:**  ROV  **Photo by:**  *Holland I* 2018  **Identified by:** Poppy Keogh  **Dive: 5** |  |  |
| **Phylum:** Chordata  **Class:** Actinopterygii  **Order:**  **Family:**  **Genus:**  **Species:** | **Morphospecies code:** ACTP7 | **Gear:**  ROV  **Photo by:**  *Holland I* 2018  **Identified by:** Poppy Keogh  **Dive: 6** |  |  |
| **Phylum:** Chordata  **Class:** Actinopterygii  **Order:**  **Family:**  **Genus:**  **Species:** | **Morphospecies code:** ACTP8 | **Gear:**  ROV  **Photo by:**  *Holland I* 2018  **Identified by:** Poppy Keogh  **Dive: 7** |  |  |
| **Phylum:** Chordata  **Class:** Actinopterygii  **Order:**  **Family:**  **Genus:**  **Species:** | **Morphospecies code:** ACTP9 | **Gear:**  ROV  **Photo by:**  *Holland I* 2018  **Identified by:** Poppy Keogh  **Dive: 6** |  |  |
| **TAXONOMY** | **NAME** | **HABITAT** | **NOTES** | **IMAGE** |
| **Phylum:** Chordata  **Class:** Actinopterygii  **Order:**  **Family:**  **Genus:**  **Species:** | **Morphospecies code:** ACTP10 | **Gear:**  ROV  **Photo by:**  *Holland I* 2018  **Identified by:** Poppy Keogh  **Dive: 7** |  |  |
| **Phylum:** Chordata  **Class:** Actinopterygii  **Order:**  **Family:**  **Genus:**  **Species:** | **Morphospecies code:** ACTP11 | **Gear:**  ROV  **Photo by:**  *Holland I* 2018  **Identified by:** Poppy Keogh  **Dive: 7** |  |  |
| **Phylum:** Chordata  **Class:** Actinopterygii  **Order:**  **Family:**  **Genus:**  **Species:** | **Morphospecies code:** ACTP14 | **Gear:**  ROV  **Photo by:**  *Holland I* 2018  **Identified by:** Poppy Keogh  **Dive: 8** | Possibly *Sebastes mentella* (P. Keogh) |  |
| **Phylum:** Chordata  **Class:** Actinopterygii  **Order:**  **Family:**  **Genus:**  **Species:** | **Morphospecies code:** ACTP15 | **Gear:**  ROV  **Photo by:**  *Holland I* 2018  **Identified by:** Poppy Keogh  **Dive: 7** |  |  |
| **Phylum:** Chordata  **Class:** Actinopterygii  **Order:**  **Family:**  **Genus:**  **Species:** | **Morphospecies code:** ACTP16 | **Gear:**  ROV  **Photo by:**  *Holland I* 2018  **Identified by:** Poppy Keogh  **Dive: 8** |  |  |
| **TAXONOMY** | **NAME** | **HABITAT** | **NOTES** | **IMAGE** |
| **Phylum:** Chordata  **Class:** Actinopterygii  **Order:**  **Family:**  **Genus:**  **Species:** | **Morphospecies code:** ACTP17 | **Gear:**  ROV  **Photo by:**  *Holland I* 2018  **Identified by:** Poppy Keogh  **Dive: 5** |  |  |
| **Phylum:** Chordata  **Class:** Actinopterygii  **Order:**  **Family:**  **Genus:**  **Species:** | **Morphospecies code:** ACTP18 | **Gear:**  ROV  **Photo by:**  *Holland I* 2018  **Identified by:** Poppy Keogh  **Dive: 8** |  |  |
| **Phylum:** Chordata  **Class:** Actinopterygii  **Order:**  **Family:**  **Genus:**  **Species:** | **Morphospecies code:** ACTP19 | **Gear:**  ROV  **Photo by:**  *Holland I* 2018  **Identified by:** Poppy Keogh  **Dive: 8** |  |  |
| **Phylum:** Chordata  **Class:** Actinopterygii  **Order:**  **Family:**  **Genus:**  **Species:** | **Morphospecies code:** ACTP20 | **Gear:**  ROV  **Photo by:**  *Holland I* 2018  **Identified by:** Poppy Keogh  **Dive: 8** |  |  |
| **Phylum:** Chordata  **Class:** Actinopterygii  **Order:** Trachichthyiformes  **Family:** Trachichthyidae  **Genus:** *Hoplostethus*  **Species:** *H. atlanticus* | **Morphospecies code:** ACTP21  *cf. Hoplostethus atlanticus* | **Gear:**  ROV  **Photo by:**  *Holland I* 2018  **Identified by:** Poppy Keogh  **Dive: 8** | Orange Roughy (P. Keogh) |  |
| **TAXONOMY** | **NAME** | **HABITAT** | **NOTES** | **IMAGE** |
| **Phylum:** Chordata  **Class:** Actinopterygii  **Order:**  **Family:**  **Genus:**  **Species:** | **Morphospecies code:** ACTP22 | **Gear:**  ROV  **Photo by:**  *Holland I* 2018  **Identified by:** Poppy Keogh  **Dive: 8** | Possibly the same as ACTP21, Orange Roughy (P. Keogh) |  |
| **Phylum:** Chordata  **Class:** Actinopterygii  **Order:**  **Family:**  **Genus:**  **Species:** | **Morphospecies code:** ACTP23 | **Gear:**  ROV  **Photo by:**  *Holland I* 2018  **Identified by:** Poppy Keogh  **Dive: 9** |  |  |
| **Phylum:** Chordata  **Class:** Actinopterygii  **Order:**  **Family:**  **Genus:**  **Species:** | **Morphospecis code:** ACTP24 | **Gear:**  ROV  **Photo by:**  *Holland I* 2018  **Identified by:** Poppy Keogh  **Dive: 9** |  |  |
| **Phylum:** Chordata  **Class:** Actinopterygii  **Order:** Gadiformes  **Family:** Macrouridae  **Genus:**  **Species:** | **Morphospecies code:** GRENADIER | **Gear:**  ROV  **Photo by:**  *Holland I* 2018  **Identified by:** Poppy Keogh  **Dive: 7** |  |  |
| **Phylum:** Chordata  **Class:** Actinopterygii  **Order:** Notacanthiformes  **Family:** Halosauridae  **Genus:**  **Species:** | **Morphospecies code:** HALOSAURIDAE  Halosauridae sp. | **Gear:**  ROV  **Photo by:**  *Holland I* 2018  **Identified by:** Poppy Keogh  **Dive: 5** |  |  |
| **TAXONOMY** | **NAME** | **HABITAT** | **NOTES** | **IMAGE** |
| **Phylum:** Chordata  **Class:** Actinopterygii  **Order:** Gadiformes  **Family:** Moridae  **Genus:** *Lepidion*  **Species:** | **Morphospecies code:** LEPIDION  *Lepidion* sp. | **Gear:**  ROV  **Photo by:**  *Holland I* 2018  **Identified by:** Poppy Keogh  **Dive: 7** |  |  |
| **Phylum:** Chordata  **Class:** Actinopterygii  **Order:** Zeiformes  **Family:** Oreosomatidae  **Genus:** *Neocyttus*  **Species:** *N. helgae* | **Morphospecies code:** OREO  *Neocyttus helgae* | **Gear:**  ROV  **Photo by:**  *Holland I* 2018  **Identified by:** Poppy Keogh  **Dive: 7** | Common name is Oreo (P. Keogh) |  |
| **Phylum:** Foraminifera  **Class:** Xenophyophorea  **Order:**  **Family:**  **Genus:**  **Species:** | **Morphospecies code:** FORAM | **Gear:**  ROV  **Photo by:**  *Holland I* 2018  **Identified by:** Poppy Keogh  **Dive: 8** |  |  |
| **Phylum:** Annelida  **Class:** Polychaeta  **Order:** Sabellida  **Family:** Sabellidae  **Genus:**  **Species:** | **Morphospecies code:** SABELLI | **Gear:**  ROV  **Photo by:**  *Holland I* 2018  **Identified by:** Poppy Keogh  **Dive: 6** |  |  |
| **TAXONOMY** | **NAME** | **HABITAT** | **NOTES** | **IMAGE** |
| **Phylum:** Arthropoda  **Class:** Pycnogonida  **Order:** Pantopoda  **Family:** Colossendeidae  **Genus:** *Colossendeis*  **Species:** | **Morphospecies code:** COLOS  *Colossendeis* sp. | **Gear:**  ROV  **Photo by:**  *Holland I* 2018  **Identified by:** Jamie Maxwell  **Dive: 6** |  |  |
| **Phylum:** Arthropoda  **Class:** Malacostraca  **Order:** Decapoda  **Family:** Chirostylidae  **Genus:** *Chirostylidae*  **Species:** | **Morphospecies code:** CRAB1  *Chirostylidae* sp. | **Gear:**  ROV  **Photo by:**  *Holland I* 2018  **Identified by:** Poppy Keogh  **Dive: 5** |  |  |
| **Phylum:** Arthropoda  **Class:** Malacostraca  **Order:** Decapoda  **Family:** Munidopsidae  **Genus:** *Munidopsis*  **Species:** | **Morphospecies code:** CRAB2  *Munidopsis* sp. | **Gear:**  ROV  **Photo by:**  *Holland I* 2018  **Identified by:** Poppy Keogh  **Dive: 5** |  |  |
| **Phylum:** Arthropoda  **Class:** Malacostraca  **Order:** Decapoda  **Family:**  **Genus:**  **Species:** | **Morphospecies code:** CRAB3 | **Gear:**  ROV  **Photo by:**  *Holland I* 2018  **Identified by:** Poppy Keogh  **Dive: 5** |  |  |
| **Phylum:** Arthropoda  **Class:** Malacostraca  **Order:** Decapoda  **Family:** Paguridae  **Genus:**  **Species:** | **Morphospecies code:** CRAB4 | **Gear:**  ROV  **Photo by:**  *Holland I* 2018  **Identified by:** Poppy Keogh  **Dive: 6** |  |  |
| **TAXONOMY** | **NAME** | **HABITAT** | **NOTES** | **IMAGE** |
| **Phylum:** Arthropoda  **Class:** Malacostraca  **Order:** Decapoda  **Family:** Munididae  **Genus:** *Munida*  **Species:** | **Morphospecies code:** CRAB5  *Munida* sp | **Gear:**  ROV  **Photo by:**  *Holland I* 2018  **Identified by:** Poppy Keogh  **Dive: 8** |  |  |
| **Phylum:** Arthropoda  **Class:** Malacostraca  **Order:** Decapoda  **Family:**  **Genus:**  **Species:** | **Morphospecies code:** CRAB6 | **Gear:**  ROV  **Photo by:**  *Holland I* 2018  **Identified by:** Poppy Keogh  **Dive: 9** |  |  |
| **Phylum:** Arthropoda  **Class:** Malacostraca  **Order:** Isopoda  **Family:**  **Genus:**  **Species:** | **Morphospecies code:** ISOPOD1 | **Gear:**  ROV  **Photo by:**  *Holland I* 2018  **Identified by:** Poppy Keogh  **Dive: 6** |  |  |
| **Phylum:** Arthropoda  **Class:** Malacostraca  **Order:** Decapoda  **Family:** Lithodidae  **Genus:** *Neolithodes*  **Species:** | **Morphospecies code:** NEOLI  *Neolithodes* sp. | **Gear:**  ROV  **Photo by:**  *Holland I* 2018  **Identified by:** Poppy Keogh  **Dive: 5** |  |  |
| **Phylum:** Arthropoda  **Class:** Malacostraca  **Order:** Decapoda  **Family:**  **Genus:**  **Species:** | **Morphospecies code:** SHRIMP1 | **Gear:**  ROV  **Photo by:**  *Holland I* 2018  **Identified by:** Poppy Keogh  **Dive: 6** |  |  |
| **TAXONOMY** | **NAME** | **HABITAT** | **NOTES** | **IMAGE** |
| **Phylum:** Arthropoda  **Class:** Malacostraca  **Order:** Decapoda  **Family:**  **Genus:**  **Species:** | **Morphospecies code:** SHRIMP2 | **Gear:**  ROV  **Photo by:**  *Holland I* 2018  **Identified by:** Poppy Keogh  **Dive: 7** |  |  |
| **Phylum:** Mollusca  **Class:** Bivalvia  **Order:**  **Family:**  **Genus:**  **Species:** | **Morphospecies code:** BIVALV1 | **Gear:**  ROV  **Photo by:**  *Holland I* 2018  **Identified by:** Poppy Keogh  **Dive: 8** |  |  |
| **Phylum:** Mollusca  **Class:** Cephalopoda  **Order:** Octopoda  **Family:** Stauroteuthidae  **Genus:** *Stauroteuthis*  **Species:** | **Morphospecies code:** OCTO1  *Stauroteuthis* sp. | **Gear:**  ROV  **Photo by:**  *Holland I* 2018  **Identified by:** Poppy Keogh  **Dive: 5** |  |  |
| **Phylum:** Mollusca  **Class:** Cephalopoda  **Order:** Octopoda  **Family:**  **Genus:**  **Species:** | **Morphospecies code:** OCTO2 | **Gear:**  ROV  **Photo by:**  *Holland I* 2018  **Identified by:** Poppy Keogh  **Dive: 8** |  |  |
| **Phylum:** Mollusca  **Class:** Cephalopoda  **Order:** Octopoda  **Family:**  **Genus:**  **Species:** | **Morphospecies code:** OCTO3 | **Gear:**  ROV  **Photo by:**  *Holland I* 2018  **Identified by:** Poppy Keogh  **Dive: 8** |  |  |
| **TAXONOMY** | **NAME** | **HABITAT** | **NOTES** | **IMAGE** |
| **Phylum:** Mollusca  **Class:** Cephalopoda  **Order:**  **Family:**  **Genus:**  **Species:** | **Morphospecies code:** SQUID1 | **Gear:**  ROV  **Photo by:**  *Holland I* 2018  **Identified by:** Poppy Keogh  **Dive: 8** |  |  |
| **Phylum:** Mollusca  **Class:** Cephalopoda  **Order:**  **Family:**  **Genus:**  **Species:** | **Morphospecies code:** SQUID2 | **Gear:**  ROV  **Photo by:**  *Holland I* 2018  **Identified by:** Poppy Keogh  **Dive: 7** | Not a squid, probably and Octopod (P. Keogh) |  |
| **Phylum:** Hemichordata  **Class:** Enteropneusta  **Order:** Enteropneusta (temporary name)  **Family:** Torquaratoridae  **Genus:** *Yoda*  **Species:** *Y. purpurata* | **Morphospecies code:** YODA  *Yoda purpurata* | **Gear:**  ROV  **Photo by:**  *Holland I* 2018  **Identified by:** Poppy Keogh  **Dive: 8** |  |  |
| **Phylum:**  **Class:**  **Order:**  **Family:**  **Genus:**  **Species:** | **Morphospecies code:** ANIMAL1 | **Gear:**  ROV  **Photo by:**  *Holland I* 2018  **Identified by:** Poppy Keogh  **Dive: 6** |  |  |
